# A mechanistic pan-cancer pathway model informed by multi-omics data interprets stochastic cell fate responses to drugs and mitogens

**DOI:** 10.1371/journal.pcbi.1005985

**Published:** 2018-03-26

**Authors:** Mehdi Bouhaddou, Anne Marie Barrette, Alan D. Stern, Rick J. Koch, Matthew S. DiStefano, Eric A. Riesel, Luis C. Santos, Annie L. Tan, Alex E. Mertz, Marc R. Birtwistle

**Affiliations:** 1 Department of Pharmacological Sciences, Icahn School of Medicine at Mount Sinai, New York, NY, United States of America; 2 Department of Chemical and Biomolecular Engineering, Clemson University, Clemson, SC, United States of America; Johns Hopkins University, UNITED STATES

## Abstract

Most cancer cells harbor multiple drivers whose epistasis and interactions with expression context clouds drug and drug combination sensitivity prediction. We constructed a mechanistic computational model that is context-tailored by omics data to capture regulation of stochastic proliferation and death by pan-cancer driver pathways. Simulations and experiments explore how the coordinated dynamics of RAF/MEK/ERK and PI-3K/AKT kinase activities in response to synergistic mitogen or drug combinations control cell fate in a specific cellular context. In this MCF10A cell context, simulations suggest that synergistic ERK and AKT inhibitor-induced death is likely mediated by BIM rather than BAD, which is supported by prior experimental studies. AKT dynamics explain S-phase entry synergy between EGF and insulin, but simulations suggest that stochastic ERK, and not AKT, dynamics seem to drive cell-to-cell proliferation variability, which in simulations is predictable from pre-stimulus fluctuations in C-Raf/B-Raf levels. Simulations suggest MEK alteration negligibly influences transformation, consistent with clinical data. Tailoring the model to an alternate cell expression and mutation context, a glioma cell line, allows prediction of increased sensitivity of cell death to AKT inhibition. Our model mechanistically interprets context-specific landscapes between driver pathways and cell fates, providing a framework for designing more rational cancer combination therapy.

## Author summary

Cancer is a complex and diverse disease. Two people with the same cancer type often respond differently to the same treatment. These differences are primarily driven by the fact that two type-matched tumors can possess distinct sets of mutations and gene expression profiles, provoking differential sensitivity to drugs. Over the past few decades, we have seen a shift away from more broadly cytotoxic drugs to more targeted molecules therapies; but how to match a patient with a specific drug or drug cocktail remains a difficult problem. Here, we build a mechanistic ordinary differential equation model describing the interactions between commonly mutated pan-cancer signaling pathways—receptor tyrosine kinases, Ras/RAF/ERK, PI3K/AKT, mTOR, cell cycle, DNA damage, and apoptosis. We develop methods for how to tailor the model to multi-omics data from a specific biological context, devise a novel stochastic algorithm to induce non-genetic cell-to-cell fluctuations in mRNA and protein quantities over time, and train the model against a wealth of biochemical and cell fate data to gain insight into the systems-level, context-specific control of proliferation and death. One day, we hope models of this kind could be tailored to patient-derived tumor mRNA sequencing data and used to prioritize patient-specific drug regimens.

## Introduction

Oncogene-targeted small molecule kinase inhibitors, like imatinib for BCR-ABL [1], have transformed chemotherapy. However, such precision medicine approaches are not always efficacious. In some cases, mutation-matched patients do not respond to the drug [2], or alternatively, resistance stochastically develops [3]. Monotherapy can even activate the target pathway, depending on cellular context [4]. Combination therapy is a logical and clinically-proven path forward [5], but rationalizing even just the choice of combinations from among the at least 28 FDA-approved [6] targeted small molecule kinase inhibitors, notwithstanding important questions related to the dozens of traditional chemotherapeutics, monoclonal antibodies, dose, timing and sequence, remains challenging for basic and clinical research. Approaches are needed that consider quantitative, dynamic, and stochastic properties of cancer cells given a context.

Computational modeling can help fulfill this need, since simulations often are much quicker and less expensive than the explosion of experimental conditions one would need to assay the drug combination space. Some of the first approaches define transcriptomic signatures that specify tumor subtypes and suggest drug vulnerabilities [7]. Big data and bioinformatic statistical approaches are at the forefront of predicting drug sensitivity, with penalized regression and other machine learning methods linking mutation or expression biomarkers to drug sensitivity [8,9].

Such statistical modeling approaches largely cannot extrapolate predictions into untrained regimes with confidence. However, this is often a major task-of-interest; for example, drug combination predictions usually only have data for drugs used alone. They also usually cannot account for dose and dynamics, central to combinations. Alternatively, mechanistic computational models based on physicochemical representations of cell signaling have an inherent ability to account for dose and dynamics. They also have greater potential for extrapolation, since they represent physical processes. Multiple mechanistic models exist for almost every major pan-cancer driver pathway identified by The Cancer Genome Atlas (TCGA): receptor tyrosine kinases (RTKs), Ras/ERK, PI3K/AKT, Rb/CDK, and p53/MDM2 [10]. An integrative model that accounts a multi-driver context has not yet been built, but will likely be needed to address drug combination prediction problems with such modeling approaches.

Here, we constructed the first mechanistic mathematical model integrating these commonly mutated signaling pathways. We use the MCF10A cell line–a non-transformed mammalian cell line with predictable phenotypic behaviors. We tailor the model to genomic, transcriptomic, and proteomic data from MCF10A cells and train the model using a wealth of literature resources as well as our own microwestern blot and flow cytometry experiments to refine biochemical parameters and phenotypic predictions. We use the model to explore stochastic proliferation and death responses to pro- and anti-cancer perturbations. This mechanistic, biological context-tailored mathematical model depicting several major cancer signaling pathways allows us to probe the mechanisms that underlie how noisy signaling dynamics drive stochastic proliferation and death fates in response to various perturbations, and gain insight into their biochemical mechanisms.

## Results

### Rationale and workflow

**Fig 1:**
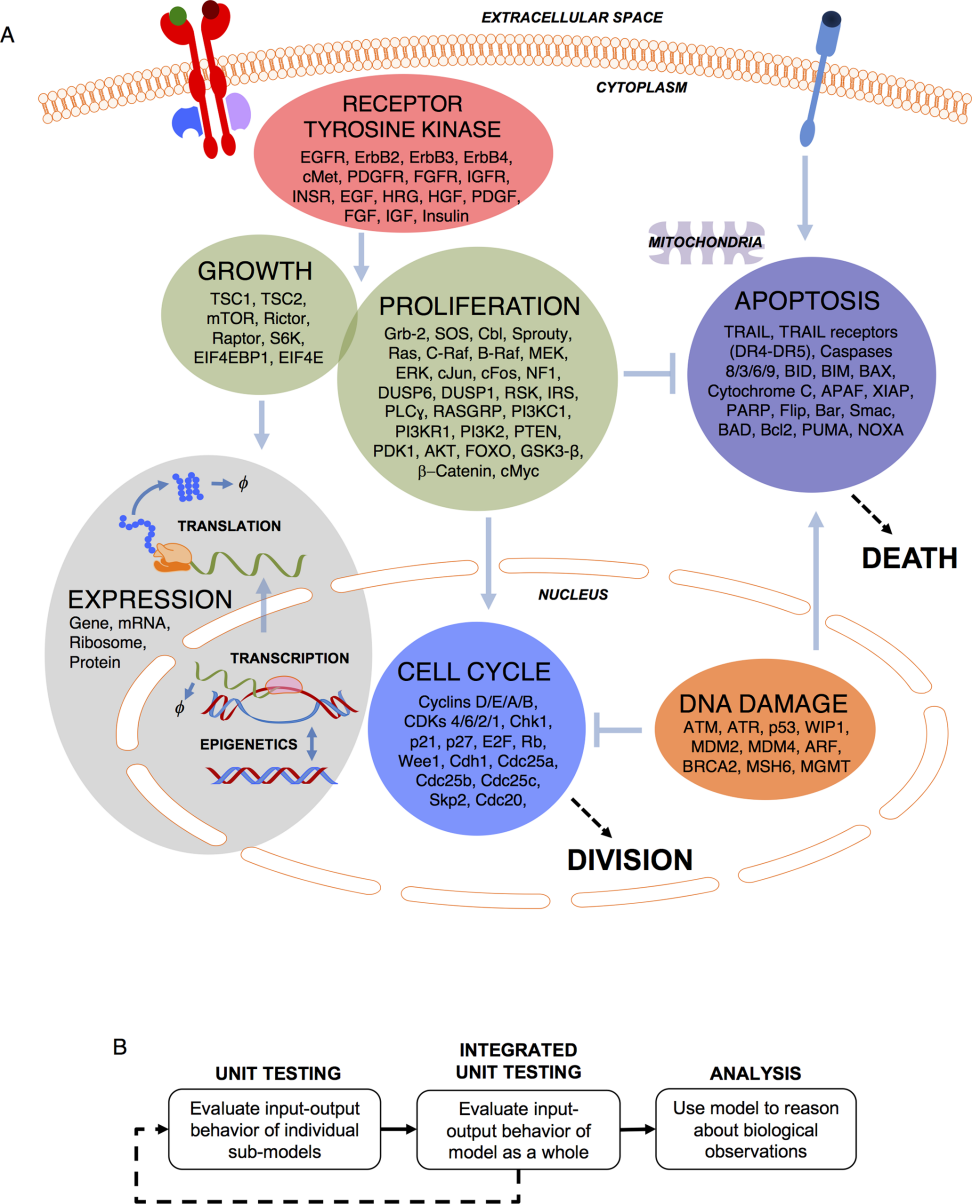
Model overview. A) Cartoon schematic of the single-cell pan-cancer driver network model. RTK, proliferation and growth, cell cycle, apoptosis, DNA damage, and gene expression submodels, with genes, compartments (capitalized italic) and general interactions indicated (light blue lines). Detailed kinetic mechanisms are in S1 Fig). B) We first evaluate individual submodel behavior (unit testing), followed by integrated unit testing of all submodels together. We then use the model to reason about biological observations.

An overarching goal of this work is a foundation that integrates canonical biochemical knowledge of cancer driver pathways with cell-specific context to make quantitative predictions about how drugs, microenvironment signals, or their varied combinations influence cell proliferation and death (Figs 1A and S1; S1 and S2 Tables). Three main considerations guided us. First is scope. TCGA identified pan-cancer driver pathways [10]: multiple RTKs; proliferation and growth (Ras/Raf/MEK/ERK; PI-3K/PTEN/AKT/mTOR); cell cycle; DNA damage. These interface with expression and apoptosis. See Fig 1A for an overview of the model (see S1 Fig for a detailed kinetic scheme) and lists of proteins included in each submodel. Second is inter- and intra-tumoral heterogeneity. Experimental accessibility defines each, which predominantly consists of copy number variation, mRNA expression and mutations, which the foundation integrates. Intra-tumoral heterogeneity is genetic [11] and non-genetic [12], so we require accounting for such noise. Third is formalism. Signaling, cell fate, and drug action are all inherently quantitative, stochastic and dynamic phenomena. Predictions for a multitude of contexts and drugs, many of which would not have yet been calibrated for, is facilitated by mechanistic as opposed to empirical models [13], so we take a mechanistic chemical kinetics approach.

We present the model as follows (Fig 1B). In “unit testing” we adapt and develop submodels for individual pathways (see Supplement) in isolation, requiring that each satisfies a set of experimentally observed biological behavior. We focus on a widely-studied non-transformed cell line, MCF10A [14] to leverage literature data and establish a baseline for “normal” cell behavior before describing transformed contexts with genetic instability and multiple poorly understood mutations. We then connect these submodels together via established signaling mechanisms to perform “integrated unit testing”, which is iterative. Finally, we analyze the model to reason about emergent signaling mechanisms underlying biological observations.

### Unit testing—Expression

#### Biology

The expression submodel depicts gene switching between an active and inactive state, transcription of the active gene to create mRNA, and translation of the mRNA to create protein. Both protein and mRNA are degraded with first-order kinetics. Although a single average gene switching rate is used for all genes [15], mRNA and protein degradation rates are informed by gene-specific half-lives taken from the literature (see S1 Table). Gene-specific transcription and translation rates are calculated from omics-data (see below). These gene-specific attributes capture dynamical behaviors that may play important roles in constraining protein function and phenotypic outcomes. Some genes possess explicitly encoded transcriptional activators and/or repressors, which can affect their transcription rate, whereas a global extrinsic effect on the translation rate of all genes is regulated by EIF4E levels (see S1 Supplemental Methods for modeling details).

#### Computationally efficient simulation of stochastic gene expression with expected cell-to-cell variability

**Fig 2:**
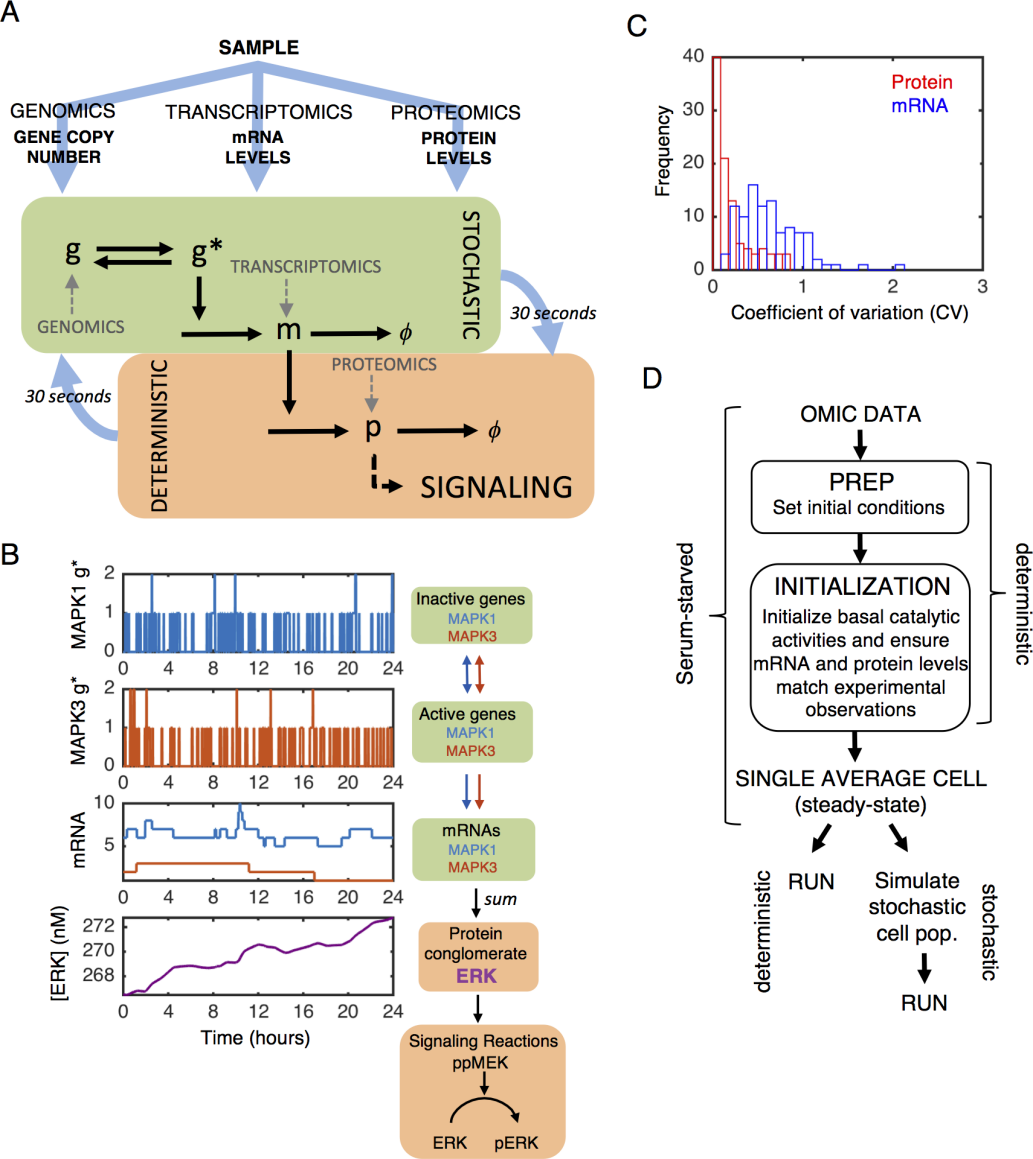
Context tailoring and stochastic gene expression. A) Gene copy number, mRNA levels, and protein levels inform initial conditions and expression submodel parameters. Gene switching, mRNA synthesis, and mRNA degradation are stochastic, whereas all other processes are deterministic, with a 30 second update interval. B) Example simulations for ERK. Two ERK isoform genes (*MAPK1*-blue and *MAPK3*-red; top two plots) switch between active and inactive states, leading to transcription noise (third plot down). Transcripts are summed into the signaling conglomerate ERK (fourth plot down). C) Coefficient of variation (CV) of protein (red) and mRNA (blue) levels across a population of 100 stochastically simulated cells after 24-hour serum-starvation. D) Overview of tailoring and running model. Multi-omic data from serum-starved cells sets initial conditions (“PREP”). Then, a deterministically-simulated initialization procedure maintains consistency with experimental observations in the presence of basal signaling activities resulting in a serum-starved single average cell at steady-state. From there, we run either deterministic or stochastic simulations. Prior to simulating any stimuli for stochastic simulations, we create a population of *N* number of cells by running the model for a 24-hour period in the serum-starved state an *N* number of times.

A major driver of cell-to-cell variability in phenotype and drug response, and therefore a component of intra-tumoral heterogeneity, is stochastic gene expression arising from low gene copy and mRNA number [12]. We derived a hybrid stochastic-deterministic model that captures random gene switching and mRNA synthesis/degradation, but treats as deterministic protein synthesis/degradation and the associated protein-level biochemistry since they involve much higher molecule number (Figs 2A and S2A and S2B). This model framework introduces natural noise into gene expression over time (Fig 2B). This expression submodel produces typical coefficient of variation (CV) of mRNA and protein levels [16–19] across a simulated population of cells (Fig 2C).

#### Tailoring to omic-data in the MCF10A context

Gene copy numbers in MCF10A (predominantly diploid) have been previously reported [21]. We serum- and growth factor-starved MCF10A cells for 24 hours and then quantified molecules per cell of mRNA and protein levels with mRNA-seq and proteomics, which show some agreement with prior studies [20] (S2F Fig). These measurements, with prior protein and mRNA stability estimates (see above and Methods), allow calculation of context- and gene-specific transcription and translation rates during the “PREP” phase of the modeling pipeline (Fig 2D). Many signaling activities consist of groups of genes (e.g. ERK1/2); we lump such expressed proteins into relevant “conglomerates” (Fig 2B and S1 Table).

#### Control of translation and ribosome dynamics

When cell growth pathways are activated, translation rates (through release of EIF4E) and ribosome synthesis (through p70S6K) increase. We derived a rate law that incorporates the effect of free EIF4E on global translation rates, which cascade into translation rates for each gene product (Methods and S2C Fig). Ribosome number must be approximately doubled within the course of the cell cycle, which constrains the rate of ribosome synthesis, and also leads to cell growth which we model as increases in volume (S2D Fig). Simulations reproduce observed positive correlations in cell-to-cell variability for protein pairs (S2E Fig) [16].

#### Protein degradation

As mentioned above, protein degradation is simulated deterministically, so fluctuations from low abundance proteins are missed, but we expect these to be a small component of overall protein level variability. There are protein-specific degradation rate constants constrained from the literature (S1 Table). Sensitivity analysis involving these parameters revealed that MEK and AKT inhibitor-induced death responses can be controlled by protein degradation rates (S6B Fig). We have not yet incorporated detailed mechanisms of protein degradation involving, for example, the ubiquitin/proteasome system. Fluctuations in the levels of proteins in these systems would contribute to extrinsic noise that is shared among proteins in a single cell, but might vary across a cell population. Incorporating such detail will be a focus of future work.

### Unit testing—Receptor tyrosine kinases

#### Biology

The receptor tyrosine kinase (RTK) submodel, inspired by several prior models [22,23], depicts ligand-binding and dimerization reactions for many receptors that are frequently overexpressed, mutated or implicated in cancer, including the ErbB family (HER1-4), the hepatocyte growth factor receptor (cMet), the platelet-derived growth factor receptor (PDGFR), the fibroblast growth factor receptor (FGFR), the insulin-like growth factor receptor (IGFR), and the insulin receptor (INSR). Ligand stimulation, for most receptors, induces receptor dimerization and phosphorylation of intracellular tyrosine residues, which recruits adaptor proteins (Grb2/SOS, PI3K, and PLCγ) to mediate downstream signaling (see S1 Supplemental Methods for more details and S2 Table for rate laws and constants).

#### Ligand-receptor cooperativity

**Fig 3:**
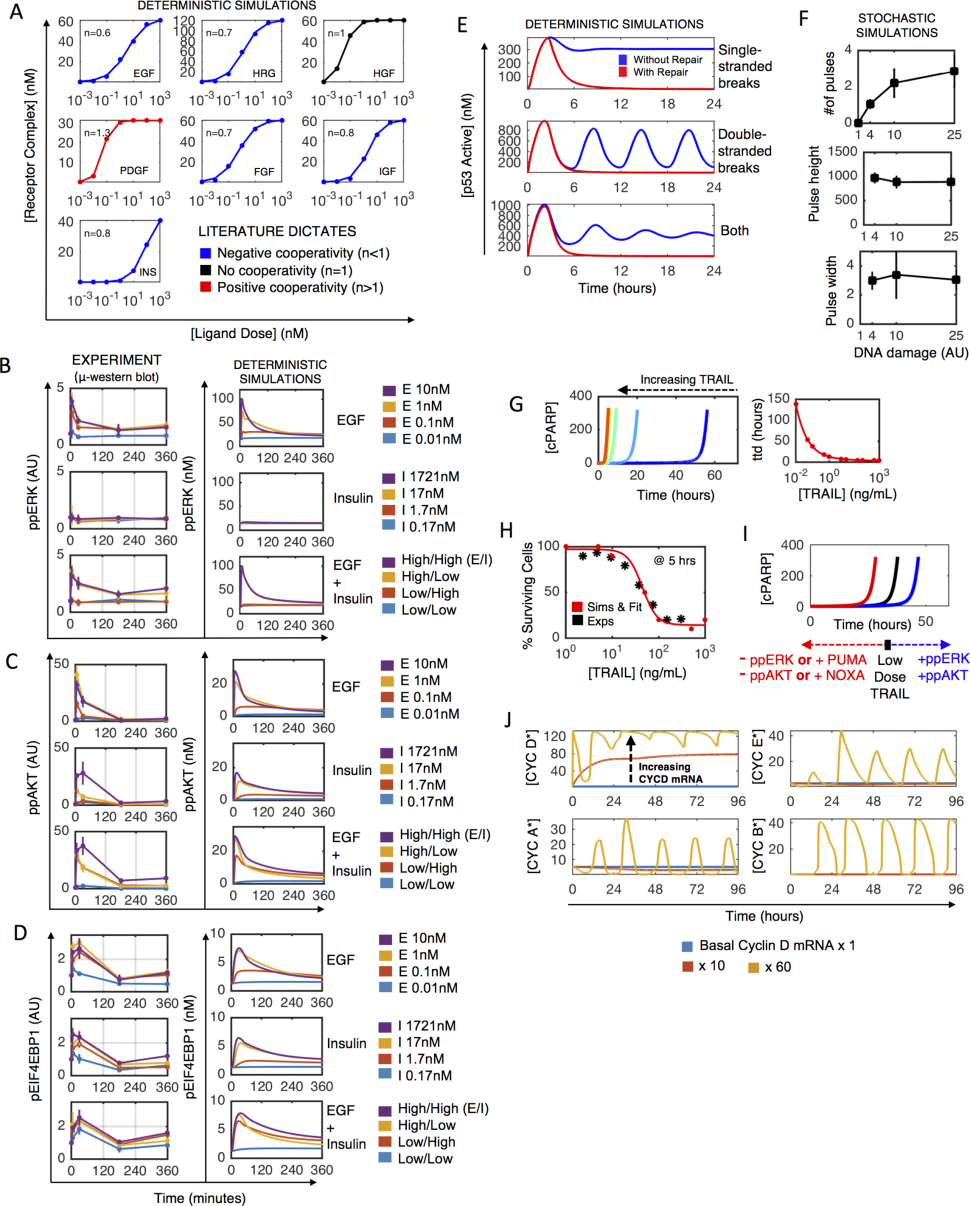
Unit testing. A) Ligand-receptor cooperativity for RTK submodel (dots-deterministic simulations; lines- hill equation fit) based on ligand-matched receptor expression (each receptor expressed in isolation and at appreciable levels). B-D) μ-Western blot data (left column) used to train simulated (right column) ERK (B), AKT (C), and mTOR (pEIF4E-BP1) (D) signaling dynamics in the proliferation and growth submodel. Serum and growth factor starved MCF10A cells were stimulated with indicated doses of EGF, insulin, and combinations (High/Low EGF 10/0.01 nM, High/Low Insulin 1721/0.17 nM) for 5, 30, 180, or 360 minutes. E) DNA damage-induced p53 responses to single-stranded breaks (top), double-stranded breaks (middle), and both (bottom), with (red) and without (blue) simulated repair. F) Stochastic simulations for how the number of p53 pulses (top), pulse height (middle) and width (bottom) depend on DNA damage level. G) Increasing amounts of TRAIL (increasingly warmer colors) decreases time to death (as indicated by cPARP spike) exponentially (right panel: red dots-simulation; line-exponential fit). H) Survival after 5 hours TRAIL treatment. Red dots-simulation; red line-sigmoid fit; black dots-flow cytometry data. I) Effects of PUMA, NOXA, ppERK and ppAKT on time-to-death in deterministic simulations. J) Simulated cell cycle initiation by increasing cyclin D mRNA levels in serum-starved cells indicates proper oscillatory behavior of cyclin-CDK complexes.

Simulated receptor-ligand binding has reasonable agreement with experimentally reported cooperativity from previous literature (Figs 3A and S3A—see references in Table SI2 in S1 Supplemental Methods). Here, we simulate the case of ligand-match receptor expression, such that only the receptors that directly bind to each ligand are present and at appreciable levels (Fig 3A); we do this to ensure the system can reproduce the traditional cooperativity behavior for each receptor, unhampered by low receptor expression or expression of binding partners. When comparing the ligand-matched receptor expression to the native MCF10A expression context (S3A Fig), one discrepancy is HRG, showing no cooperativity here but was reported to have negative cooperativity [24]. HRG binds to ErbB3 and ErbB4 receptors which heterodimerize with ErbB2, all of which have context-specific expression. ErbB2 does not bind ligand and is therefore predicted to make cooperativity less negative for this class of homo and heterodimerizing receptors (including ErbB1/EGFR).

#### Receptor activation and trafficking dynamics

Large ligand doses activate (i.e. receptor or adapter phosphorylation) receptor pools within ~min time scales that are coincident with receptor-specific trafficking dynamics occurring on the order of 5 minutes to 1 hour (S3B Fig) [26,27]. Modeled RTKs were consistent with experimental observations for surface vs. internalized receptors and previous models [28], such as the transient nature of EGF-induced signaling relative to other ligands (S3C and S3D Fig).

### Unit testing—Proliferation and growth

#### Biology

The proliferation and growth submodel, designed as an amalgamation of several prior models [23,29,30] in addition to new mechanisms, depicts the binding of adaptor proteins to phosphorylated receptor tyrosine residues leading to the activation of the Ras/ERK and PI-3K/AKT signaling pathways. Receptor activation leads to internalization, signaling to the ERK pathway from early endosomes, and receptor degradation with some receptors being recycled back to the cell surface. The activation of the ERK and AKT pathways leads to the upregulation of cyclin D through the production of AP1 and cMYC, respectively. AKT activation can lead to mTOR activity, which exerts global control over translation efficiency via regulation of EIF4E. Negative feedback exists in both in the ERK and AKT pathways at the level of Raf and PI3K, respectively. Finally, both ERK and AKT mediate negative regulation of apoptosis signaling through BIM and BAD.

#### Receptor pathway preferences

We incorporated receptor/adapter affinity and avidity patterns based on prior experimental literature and computational models (S1 Supplemental Methods). For example, ErbB3, IGF1-R and Ins-R all favor PI-3K/AKT signaling heavily as compared to Ras/ERK signaling, which is recapitulated by our model (S3C Fig). Because our model is tailored to the MCF10A context, certain ligand simulations (FGF, PDGF) do not induce appreciable activation of the ERK or AKT pathways (S3C Fig), likely due to low receptor expression. This observation is consistent with public data available in LINCS (S3D Fig) [25], with the exception of IGF, which has greater ERK activation than predicted.

#### Basal activity levels

The model describes futile cycles through the Ras and PI-3K pathways (GTP bound Ras and PI(3,4,5)P), and thus accounts for the natural thresholding and negative feedback that prevents such basal activity from influencing phenotype, regulates cellular homeostasis and provides robustness to noise. This causes basal ERK and AKT kinase activity, which constrains phenotype predictions during integrated unit testing (below).

#### Dose and dynamic responses

We collected dose-dependent time course data using the microwestern array (meso-scale quantitative western blot) for the ERK (pERK), AKT (pAKT), and mTOR (pEIF4E-BP1) signaling pathways in serum-starved cells treated with to EGF, insulin or the combination (Figs 3B–3D and S4). Due to the varied and dose-dependent pathway preferences of these ligands, we reasoned this dataset would provide constraints on the downstream fluxes. By varying relative fluxes through activated receptors to Ras, PI-3K, ERK, AKT and mTOR, simulations agree reasonably well with this entire cohort of data simultaneously (Fig 3B–3D). These data show the strong influence of insulin on AKT but not ERK signaling, and also transient EGF-induced signaling as reported in multiple previous studies [31].

#### Cell-to-cell heterogeneity in signaling response

In order to evaluate the extent to which the model can capture cell-to-cell heterogeneity in signaling response, we compared ERK activity between simulations and experiments in response to pathway activators. We collected single-cell microscopy data of MCF10A cells stably expressing a live-cell reporter of ERK activity [32]. This kinase translocation reporter (KTR) moves from the nucleus to the cytoplasm upon phosphorylation by ERK. The cells were serum-starved then stimulated with EGF plus insulin. Qualitatively, there are two features that the model recapitulates from the imaging data (S5A and S5B Fig). First is approach to a steady-state that is slightly above baseline signaling levels. Second is the uniformity and unimodality of the responses. Other studies have reported binary on-off type switching in different cell systems [33] or in MCF10A cells but in a state of chronic growth medium treatment is asynchronous cells [34]. However, in this phase of acute response to growth factors, ERK activity seems to have uniform and unimodal response, which the model captures. Quantitatively, this imaging data is discrepant from the microwestern data. Because the microwestern assay was quantitatively validated, we do not require fold-change agreement with this imaging data.

### Unit testing—DNA damage

#### Biology

The DNA damage submodel, derived primarily from Batchelor et al. [35], depicts the activation of ATR and/or ATM by single/point damage and/or double strand breaks, which results in oscillatory or sustained p53 dynamics, respectively. The activation of p53 leads to MDM2 and WIP1 production, which serve as negative feedback regulators on p53 activity. Here, we model etoposide as the principal stimulus of DNA damage, which induces damage primarily when cells are cycling (see S1 Supplemental Methods). We incorporated DNA repair to either single/point damage (MSH6, MGMT) or double (BRCA2) strand breaks (Fig 3E). We required that levels of damage significant enough to induce p53 response should be repaired within the hours-day time scale.

#### Stochastic regulation of p53 pulsing amplitudes and dynamics

Double strand breaks cause p53 pulses of roughly equal amplitude and timing, but increase in number as DNA damage dose increases, with this number varying across a cell population [36]. We specified ultrasensitive activation of ATM and ATR by DNA damage and tuned DNA repair activities (properties hypothesized by [36]) to ensure our model recapitulated these features (Figs 3F and S3E).

### Unit testing—Apoptosis

#### Biology

The apoptosis submodel, derived primarily from Albeck et al. [37], depicts the activation of initiator caspases (caspase 8 and 10) by TRAIL through the death receptors (DR4/5), which leads to the activation of executioner caspases (caspases 3 and 7), leading to cell death. The activation of caspase 8 can also lead to the activation of Bax via tBid activity. Bax oligomerization drives pore formation in the mitochondria and the release of cytochrome C, which provokes apoptosome formation, the activation of caspase 3, and subsequently, the cleavage of PARP (cPARP), a common readout for apoptosis [38]. In addition to extrinsic apoptosis, there are also intrinsic (intracellular) modes of apoptosis signaling, which act through the regulation of pro- (e.g. BAD, Bim) and anti- (e.g. Bcl2) apoptotic proteins by pro-survival (active ERK and AKT) or pro-death (PUMA and NOXA, upregulated in response to DNA damage) proteins. This regulation acts through the mitochondrial-dependent pathway to promote or prevent cell death.

#### Extrinsic pathway dose and dynamic response

The time-to-death of a cell decreases with increasing TRAIL dose [37], which simulations reproduce (Fig 3G), like the source model. Stochastic simulations match our percent death data for the TRAIL dose response (Fig 3H), similar to previous work [39].

#### Survival signaling and intrinsic pathway

DNA damage causes p53-mediated upregulation of pro-apoptotic PUMA and NOXA, but survival signaling activity of the ERK or AKT pathways can counteract this stimulus. The model captures these general trends of PUMA/NOXA and ERK/AKT activities on time-to-death (Fig 3I).

### Unit testing—Cell cycle

#### Biology

The cell cycle submodel, derived primarily from Gérard and Goldbeter [40], is initiated by the upregulation of cyclin D mRNA by AP1 and cMyc in G0 phase. Active Cyclin D (bound to Cdk4 or Cdk6), phosphorylates Rb to de-repress E2F transcription. E2F upregulates cyclin E and cyclin A, which bind to Cdk2 to become active, marking the beginning of S-phase and lasting into G2 phase. Finally, the activation of cyclin B/Cdk1 marks the beginning of mitosis, which, when complete, returns the cells to G1 phase.

#### Expected order and timing of cyclin/cdk complex activities by Cyclin D induction

Simulated Cyclin D transcriptional activation initiates the cell cycle (Fig 3J). As in the original model, the order and timing of Cyclin expression is maintained [40], and these orders correspond to distinct phases of the cell cycle (Fig 3J). However, stochastic gene expression caused cell cycle deregulation, as found previously [41], suggesting as-yet undiscovered mechanisms of cell cycle robustness. We could not rectify this so cell cycle species do not couple to stochastic gene expression.

### Integrated unit testing—Model initialization in a serum-starved state

Each submodel unit is now constrained by a variety of biological observations and data, so we move to integrated unit testing. We do not imply that submodels are “correct”. Rather, the presented characterization simply increases confidence that submodels reproduce expected cellular behavior, which of course is predicated upon the considered data and assumptions. We first evaluate integrated behavior in the reference state—serum starvation.

#### Adjusting translation due to protein dispersion

After translation, proteins undergo post-translational modifications (PTM) and/or are recruited into complexes, which can change degradation rates (e.g. GSK-3β destabilizes β-Catenin). Thus, in the integrated model, total protein levels no longer agree with proteomics data. We therefore iteratively adjust effective translation rate constants to retain such agreement; this occurs during the initialization procedure (Fig 2D and S1 Supplemental Methods).

#### Cell fate dynamics

**Fig 4:**
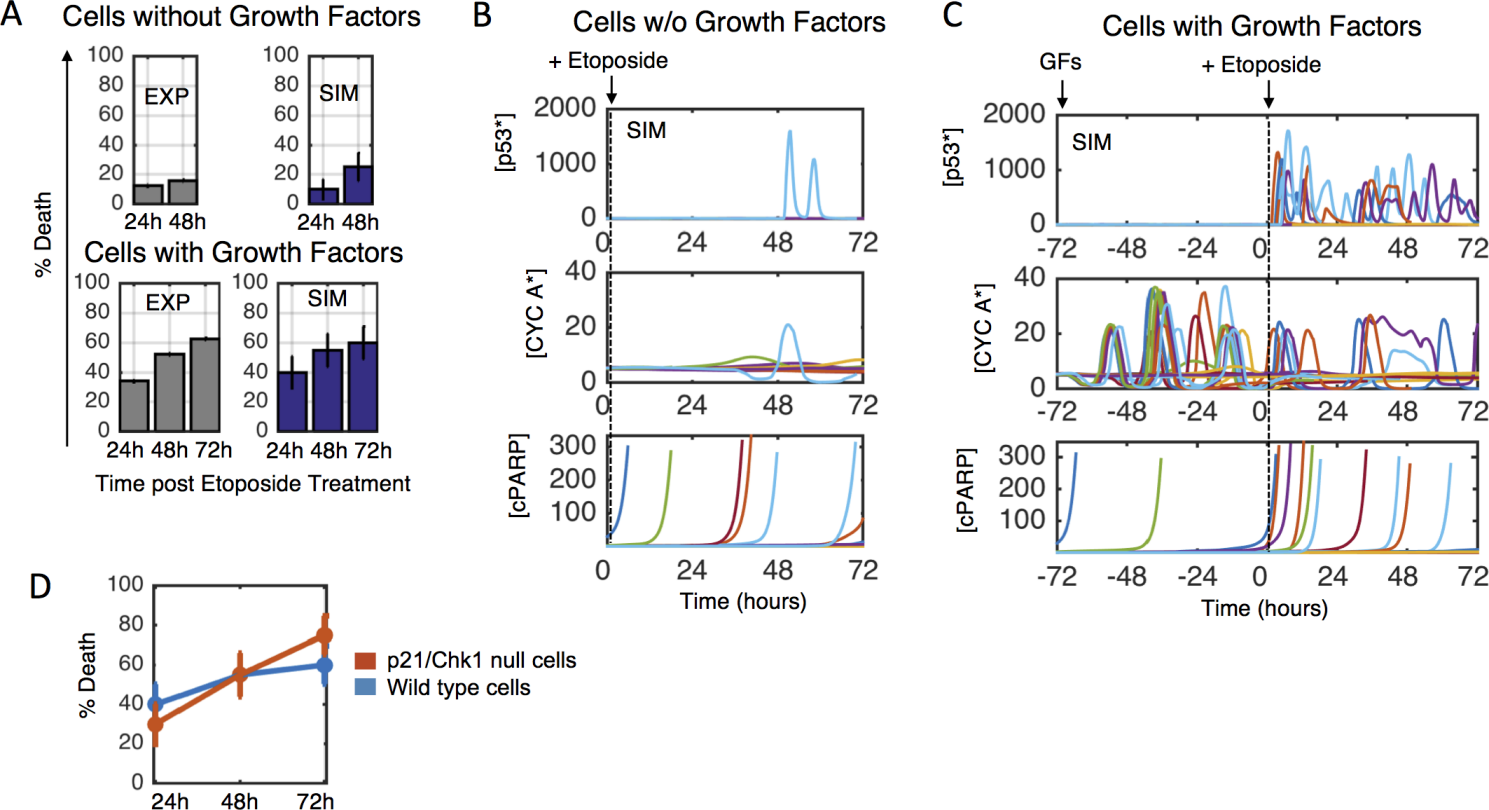
Etoposide response dynamics in dividing and non-dividing cells. A) Experiments (left, grey, flow cytometry) and simulations (right, blue) for MCF10A cells treated with etoposide (100μM) for 24, 48, and 72 hours in the presence of no growth factors (top) or EGF (20ng/mL) + insulin (10μg/mL) (bottom). B-C) Stochastic simulations for (B) serum-starved or (C) EGF + insulin with 72 hour etoposide treatment. D) Quantification of simulation results from (C) for wild type (blue) or p21/Chk1 loss cells (red).

Flow cytometry showed that in a serum- and growth factor-starved cell state, approximately 10% more cells die every 24 hours (Fig 4A), and active cell cycling is negligible. These data constrained the effects of proliferation and growth submodel outputs (primarily ERK and AKT activities) on cyclin D expression (cell cycle) and BAD/BIM (apoptosis), and thus the effects of basal signaling activities on phenotype.

### Integrated unit testing and analysis—Cell cycle-dependence of chemotherapy

The chemotherapeutic etoposide induces DNA damage. We treated serum-starved MCF10A cells with etoposide for 24 or 48 hours, but found negligible induction of cell death over control (Fig 4A-top). This is consistent with etoposide-induced cell death being amplified by DNA replication [42], which we also observed (EGF + insulin, Fig 4A-bottom). We postulated a pharmacodynamic model where etoposide-induced DNA damage is strongly dependent on S-phase cyclins (see S1 Supplemental Methods) that reproduced the observed cell death results (Fig 4A–4C). Very few simulated cells without growth factors show a p53 response or active cycling (Cyclin A), and therefore very little cell death (cleaved PARP). With EGF and insulin most simulated cells show a robust and sustained p53 pulsing response, which leads to cell cycle arrest (through p21 and Chk1) and cell death.

We noticed that etoposide-induced cell death in the presence of EGF and insulin plateaus over time in experiments and simulations at around 60%, as opposed to increased cell killing over time. We reasoned that this was due to p21/Chk1-induced cycle arrest. In simulations where p53 has limited ability to upregulate p21, etoposide is predicted to cause greater cell death (Fig 4D). This result is supported by studies showing that cells with mutated p21 are more sensitive to DNA damaging agents [43–45]. Furthermore, several cancer cell lines from the Genomics of Drug Sensitivity in Cancer Project with p21 mutations exhibit increased sensitivity to etoposide [9]. Thus, our model recapitulates the complex dynamic response to a common chemotherapy and predicts features of its context-dependent efficacy.

### Integrated unit testing and analysis—Synergistic inhibitors for stochastic apoptosis

**Fig 5:**
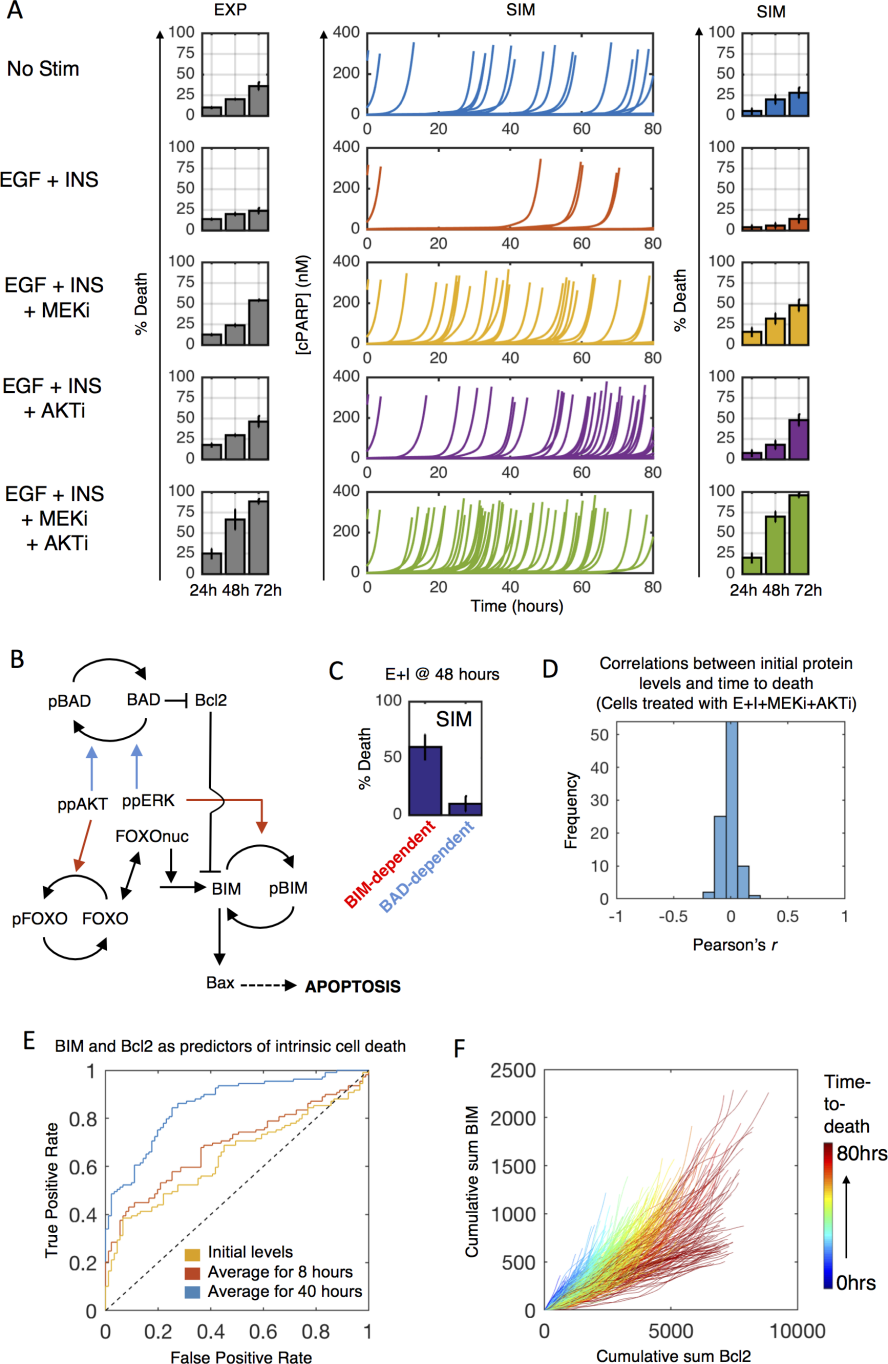
Intracellular regulation of stochastic apoptosis. A) Serum-starved MCF10A cells were treated as indicated (EGF-20ng/mL, insulin-10μg/mL, MEKi-10μM, AKTi-10μM) and assayed for cell death by flow cytometry (left, grey bars). Single cell simulations (middle, each line) show time to death (indicated by cPARP spike), quantified by colored bars (right). Colors represent treatment condition. B) Model structure for ppERK and ppAKT regulation of apoptosis through BAD (blue arrows) and BIM (red arrows). C) Simulations where BAD-dependent (blue arrows in B) or BIM-dependent (red arrows in B) mechanisms were switched off and percent death calculated in response to EGF + insulin at 48 hours. D) Correlation of initial protein levels with time-to-death for 400 simulated cells treated with EGF + insulin + MEKi + AKTi. E) ROC curves for predicting cell death from time-averaged BIM and Bcl2 levels based on 400 simulated cells (200 training/200 validation) treated as in (D) (blue-AUC = 0.86; red-AUC = 0.69; yellow-AUC = 0.64). F) Cumulative sum of BIM and Bcl2 levels for simulated cells up until their time of death for all conditions in (A). Color shows time to death.

The mechanisms by which oncogene-targeted drugs cause cell death through loss of survival signaling are not as well understood as their cytostatic effects. We measured cell death dynamics (assayed via Annexin-V/PI flow cytometry) in response to small molecule inhibitors of ERK and AKT pathways (downstream mediators of many oncogene-targeted drugs) alone or in combination, with serum-starvation or EGF + insulin (Fig 5A). As before, serum-starvation induces cell death, which is attenuated by EGF + insulin. Inhibition of either ERK or the AKT pathway alone increases cell death 72 hours post treatment, but negligibly at 48 hours. Combining the two inhibitors revealed synergy in cell death induction at 48 hours, and much more cell death at 72 hours. The integrated model captures these cell death dynamics reasonably well (Fig 5A—compare far right to far left).

We used the integrated model to reason about what mechanisms may underlie the synergy between and overall impacts of ERK and AKT inhibition for cell death. Both ERK and AKT can phosphorylate and inactivate the pro-apoptotic function of BAD, ERK can phosphorylate BIM causing similar inhibition of pro-apoptotic function, and AKT-mediated deactivation of FOXO downregulates BIM levels (Fig 5B). Simulations where either BAD- (blue arrows) or BIM-dependent (red arrows) mechanisms were computationally knocked-out predicted BIM to account for most ERK and AKT inhibition effects (Fig 5C). Independent experimental data corroborate this prediction; MCF10A cell detachment-induced death, which depends on such survival signaling mechanisms, has been observed to be predominantly controlled by BIM rather than BAD [46,47]. The model suggests several contributing mechanisms. One reason is expression levels; BAD is lowly expressed compared to anti-apoptotic BCL2 family proteins (~14,000 vs. ~61,000 molecules per cell), and thus is unlikely to exert significant control over cell death. Another is the cascade network structure for BIM regulation. If only AKT is inhibited, phosphorylation by ERK blocks the accumulation of active BIM. If only ERK is inhibited, the amount of BIM in the cell, even if completely unphosphorylated, is largely insufficient to drive cell death in the absence of further strong death signals. However, when both pathways are inhibited, the production and accumulation of active BIM proceeds uninhibited, thus creating a potent apoptotic signal. Furthermore, the inhibition of these activities must be coordinated over relatively long periods of time, with duration of AKT inhibition having enough overlap with that of ERK to cause both the accumulation and activation of BIM.

Why do some cells die early and others die late following ERK and AKT inhibition? We used simulation data to gain insight into this question. First, we asked whether initial total protein levels (sum across all forms of a protein) were correlated with time-to-death after ERK and AKT inhibition. In line with conclusions from prior studies focused on extrinsic death pathways [12], time-to-death was not significantly correlated with the initial levels of any single protein (Fig 5D). This suggests that stochastic processes post-inhibitor treatment largely dictate variable cell death fate. We performed lasso regression using time-averaged protein levels as predictors of early or late death (pre/post 40 hours) over 400 individual cell simulations. This analysis identified time-averaged BIM and BCL2 of the top explanatory variables. We note that the temporal dependence of explanatory variables reveals a dynamic signature of the pathway. Next, we trained a support vector machine (200 cells for training, 200 for validation) and found that average BIM and BCL2 levels over a 40-hour time course (or as long as a cell lived) are highly predictive for death timing (Fig 5E). Initial levels, or even those averaged over the first 8 hours post-stimulus, were moderately predictive at best. As one might expect based on the respective pro- and anti-apoptotic functions of BIM and BCL2, respectively, stochastic expression time courses that tend towards high BCL2 and low BIM are the most protective (Fig 5F). Interestingly, these predictors of stochastic death phenomena were not equally applicable to different death stimuli such as the extrinsic ligand TRAIL (S6A Fig), thought to depend more on stochastic activation dynamics of Caspase 8 [12], which highlights the treatment-specificity of stochastic cell fate determinants.

Overall, these results suggest that intrinsic death of individual cells induced by inhibition of survival signals may be inherently unpredictable prior to treatment. Such a claim is inherently difficult to prove conclusively, since there always might exist alternative measurable predictors and/or methods of analysis that render such prediction possible. Moreover, in our specific case, we have not yet incorporated detailed stochastic mechanisms of protein degradation, which when included may provide additional informative co-variates.

### Integrated unit testing and analysis—Synergistic mitogens for stochastic cell cycle entry

**Fig 6:**
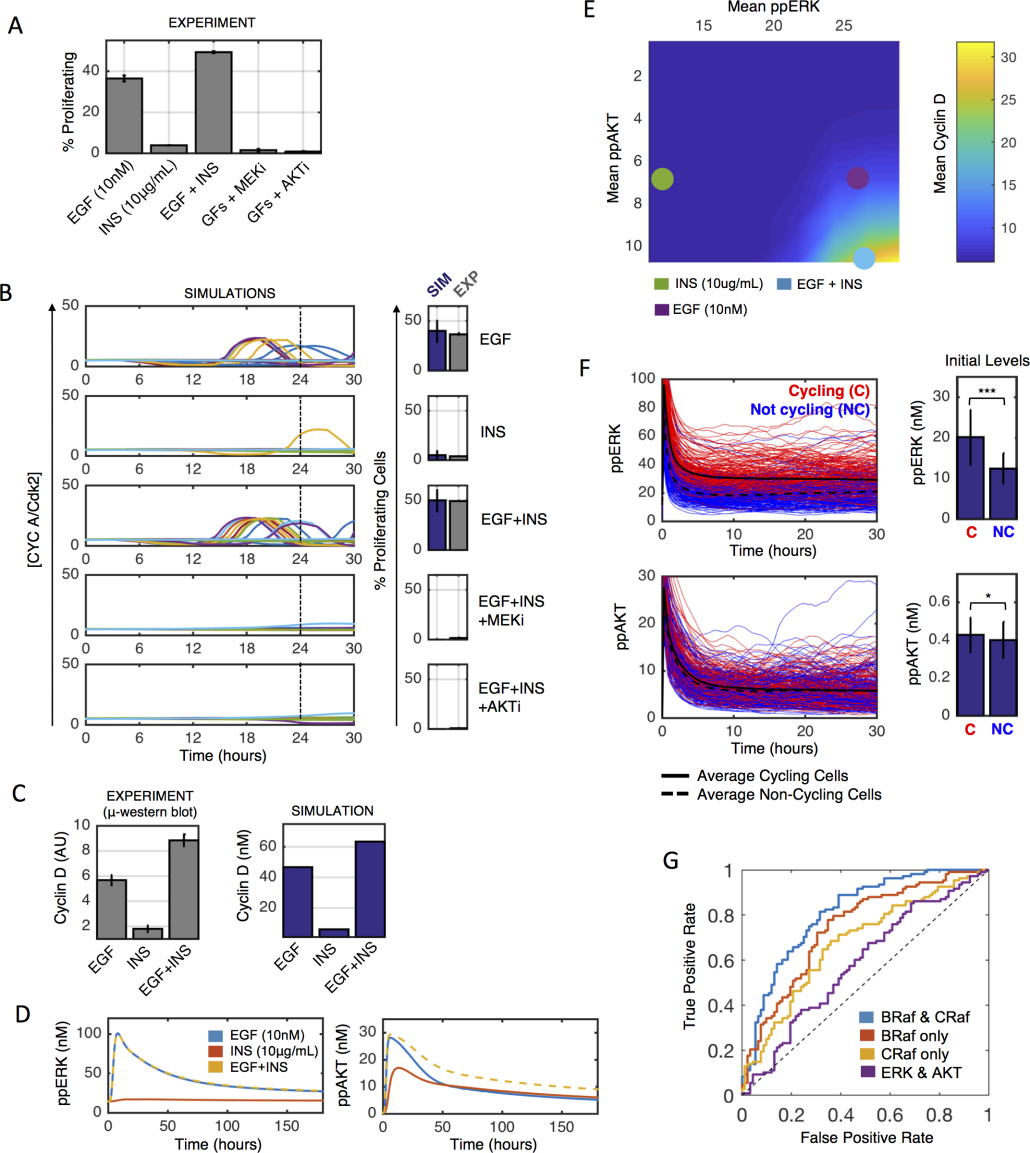
Regulation of stochastic proliferation. A) Serum-starved MCF10A cells were treated as indicated for 24 hours and then assayed for cell cycle progression via flow cytometry (MEKi-10μM; AKTi-10μM). Percent proliferating cells are S-phase plus G2/M minus that in serum-starved controls (<~5%). GFs: Growth Factors (EGF + insulin). B) Model fit. C) Measured (left) and simulated (right) cyclin D levels 6 hours post stimulation of serum-starved cells. D) Deterministic simulations of ppERK (left) and ppAKT (right) response of starved cells to indicated growth factors. E) Simulated relationships between time-averaged ppERK and ppAKT with cyclin D levels (heatmap colors). Colored circles indicate values for different treatments. F) 400 simulated cells treated with EGF + insulin were cycling (red; divided before 30 hours) or non-cycling (blue; did not divide before 30 hours). Right: Initial levels of ppERK or ppAKT in the same simulations. G) ROC curves predicting proliferation from 400 simulated cells (200 training/200 validation) following EGF + ins from initial BRaf and CRaf levels (blue-AUC = 0.81; red-AUC = 0.74; yellow-AUC = 0.68; purple-AUC = 0.59).

EGF and insulin regulate MCF10A proliferation. We measured (BrdU incorporation/flow cytometry) how these two ligands influence cell cycle progression of serum-starved MCF10A cells and how the ERK and AKT pathways were involved (Fig 6A). Insulin induces negligible cell cycle entry on its own but synergizes with EGF (Fig 6A), which is also seen on the level of cyclin D expression (Fig 6C). The ERK and AKT pathways are both essential to drive EGF + insulin-induced cell cycle progression (Fig 6A). After tuning the dependencies of transcriptional processes downstream of the ERK and AKT pathways, the integrated model reproduces these data (Fig 6B and 6C).

What underlies the synergy between EGF and insulin with regards to cell cycle progression? Prior work has suggested that the dynamics of the ERK pathway can determine cell proliferation fate [48]. However, data and simulations suggest that ERK pathway activation dynamics are essentially identical for EGF or EGF + insulin (Fig 6D). Insulin is a very strong activator of AKT but not ERK signaling (Fig 6D). When cells are treated with EGF + insulin, there are negligible differences in acute (<30 min) AKT activation dynamics, and significant differences only develop over long (hours) time scales. Thus, the mitogenic synergy between EGF and insulin seems associated with prolonged AKT pathway activity over long time scales. Simulations suggest that increasing mitogen-induced cyclin D expression cannot be accomplished by only increasing the activity of one pathway, but rather roughly equal amounts of both time-integrated ERK and AKT activity are needed to drive more robust mitogenic responses (Fig 6E). This interpretation cannot rule out coincident factors such as increased insulin-induced glucose uptake, but does suggest that the coordinated activation of both ERK and AKT for several hours post-mitogen treatment seem important to drive cell cycle entry, rather than acute activation dynamics following mitogenic stimulus.

These observations suggested to us that stochastic differences from cell-to-cell in AKT activity dynamics might be predictive for EGF + insulin-induced cell cycle entry. Surprisingly, in simulation results, neither the initial AKT activity nor dynamics post-stimulus contained significant discriminatory information related to subsequent cell cycle entry decisions of individual simulated cells (Fig 6F, bottom). Rather, the ERK activity dynamics were much more predictive of subsequent stochastic cell cycle entry (Fig 6F, top). This is consistent with prior live-cell imaging work that showed a strong correlation between time-integrated ERK activity and resultant S-phase entry decisions [34], albeit in a setting of much higher cell confluence than our experiments here entail and with additional mitogenic factors present (hydrocortisone and cholera toxin). Why would AKT activity dynamics control synergy between EGF and insulin but not be predictive of stochastic cell fate? AP1 and cMyc control cyclin D expression in this model. AP1 (cFos-cJun) activity has a sharp response to time-integrated ERK activity, relative to the less sharp dependence of cMyc expression on AKT activity. This sharpness difference is due to positive autoregulation of cJun expression by AP1 [49], and is likely also influenced by the already high expression of cMyc in serum-starved MCF10A (~48,000 molecules/cell, compared to 0 and ~3,000 for cFos and cJun), which is one of the few MCF10A alterations (they also have CDKN2A/ARF loss, which is captured in our gene copy number data) [50]. We also wondered whether higher AKT activity lowers the threshold of ERK activity needed to cause cell cycling; however, we found this not to be the case (S7D Fig).

Could stochastic cell cycle entry be predicted by protein abundance fluctuations prior to EGF + insulin treatment? We again applied lasso regression using initial conditions for total protein levels in individual simulated cells to identify explanatory variables for cell cycle entry, and then trained a support vector machine classifier. In contrast to predicting death in individual cells, here we found that initial CRaf and BRaf levels were fairly predictive of whether a cell would enter the cell cycle by 24 hours post EGF + insulin stimulus (Fig 6G). Initial total ERK and AKT (active + inactive) levels were not predictive at all (Fig 6G). We conclude that so long as AKT activity is prolonged, ERK activity fluctuations likely control stochastic cell proliferation fate in response to acute growth factor stimulus. These ERK activity fluctuations across a cell population seem heavily influenced by initial Raf protein levels.

### Analysis—Predicting initial stages of transformation fitness

**Fig 7:**
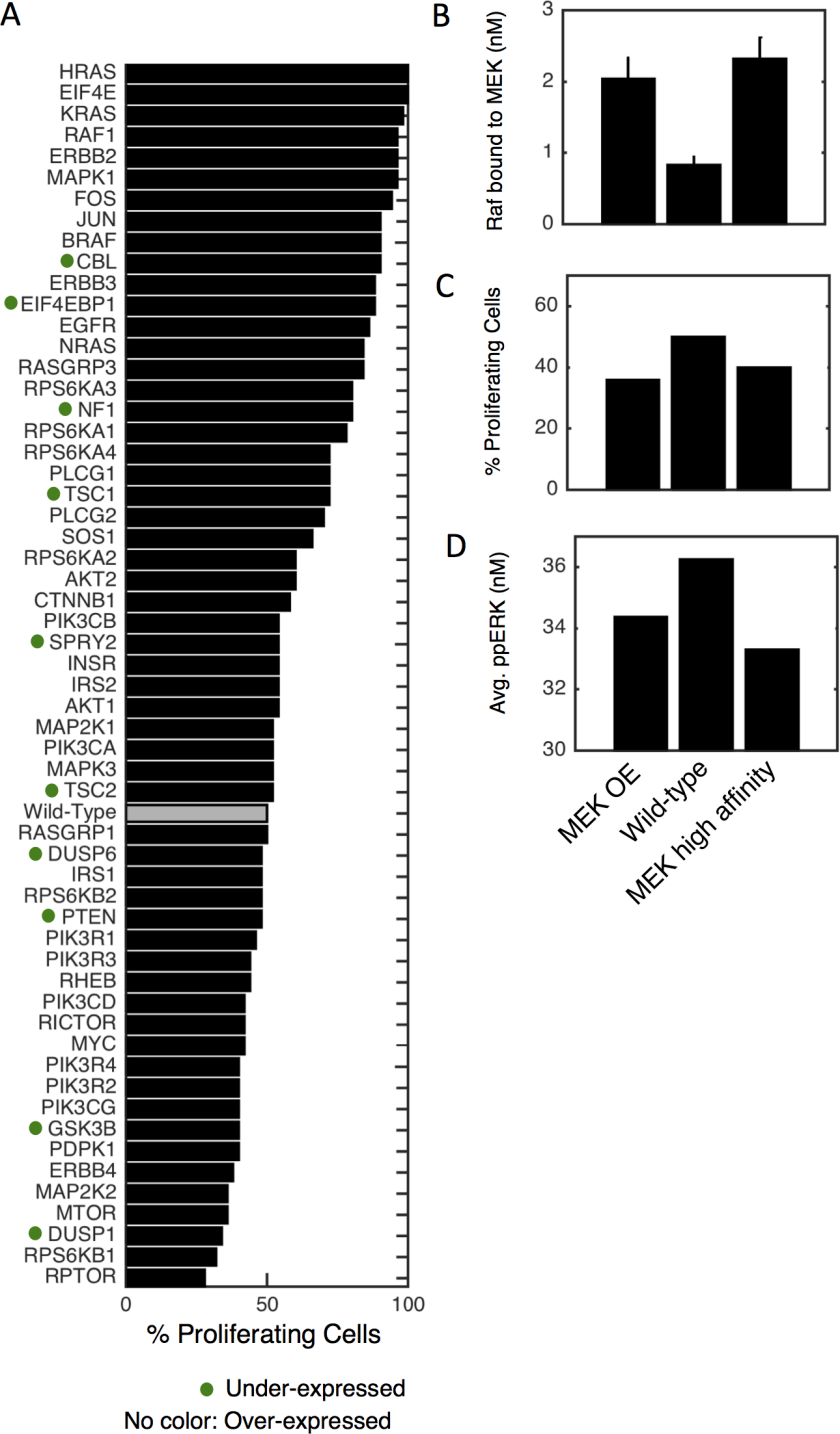
Simulated transformation. A) The mRNA level of each gene is altered 10-fold and EGF + insulin response of starved cells for 24 hours is simulated. Gray bar denotes wild-type, dots denote tumor suppressors (10-fold reduction), and no label denote proto-oncogenes (10-fold increase). B-D) MAP2K2 overexpression (OE-left), wild-type (middle), and MEK-Raf high affinity (right, unphosphorylated MEK has 10-fold higher affinity for all Raf species) were simulated as above and either the level of Raf bound to MEK at t = 0 (B), % proliferating cells (C) or time-averaged ppERK levels (D) were calculated.

The model contains multiple oncogenes and tumor suppressors, and is calibrated to a non-transformed epithelial cell state. We wondered what simulations would predict the transformation potential to be for each gene. To test this, we systematically altered expression of each RTK, proliferation and growth submodel gene (10-fold up for oncogenes, 10-fold down for tumor suppressors), simulated proliferation response to EGF + insulin (emulate a growth factor-containing microenvironment), and ranked nodes by this metric (Fig 7A). Several expected high-ranking results are observed, such as Ras, Raf, ErbB2/HER2 and EGFR, those with well-established transforming viral analogs such as cFos, cJun and cCbl, and more recently recognized translation control with EIF4E and EIF4E-BP1 [51]. Gene products along the PI3K axis (PIK3Cα, PTEN, β-Catenin, cMyc) are lower ranked, which seems counter-intuitive since these are also frequent cancer drivers. However, MCF10A cells, while non-transformed, are immortal and fast growing in culture indefinitely, and one of the reasons (as stated above) is cMyc overexpression. The cMyc protein is downstream biochemically of PI3K/AKT, its overexpression occurs as a resistance mechanism in response to PI3K inhibitor therapy, and PI3K and cMyc alterations tend to be mutually exclusive [52–54]. Thus, we interpret these results as being consistent with the MCF10A context being naturally less reliant on these PI3K axis gene products to drive increased proliferation.

One paradoxical result is the low ranking of MEK (MAP2K1/2), despite being functionally surrounded by highly ranked genes. This result is actually consistent with widespread clinical data that seldom report MEK alterations (~<5% TCGA pan-cancer in cBioPortal) as compared to Ras/Raf alterations, the explanation for which remains unclear. What mechanisms might explain MEK’s poor simulated transforming potential? In simulations, free C/B-Raf—those that are available to transmit signals from active Ras—are reduced, and most of this difference ends up sequestered by MEK itself, which seems to reduce active ERK (Fig 7B–7D). Simulations where MEK has artificially high affinity for MEK phenocopy this result. We interpret this as a consequence of stoichiometry and expression levels, since both C-Raf and B-Raf are relatively lowly expressed (~29,000 mpc and ~2,800 mpc, respectively) as compared to MEK (MAP2K1~147,000 mpc; MAP2K2~378,000 mpc), so although this sequestration effect might be relatively small, it can account for a non-negligible proportion of the total Raf pool. Thus, the prediction from these simulations is that MEK typically has poor transforming potential because increasing its levels it tends to sequester Raf from increased signaling as opposed to allowing for increased signal transmission. This of course does not fully explain observations related to the very low frequency of MEK activating mutations, which convolve protein structural robustness, and also potentially genome organization factors. Nevertheless, such a mechanism would not be possible to uncover from genomics or network model-based approaches because it is inherently based in quantitative biochemistry that our model captures from its formulation. Future work will have to perform a thorough genetic screen to experimentally validate these simulation-derived predictions regarding the transformation potential of single gene modifications.

### Analysis—Predicting cell death responses in U87 glioma cells

**Fig 8:**
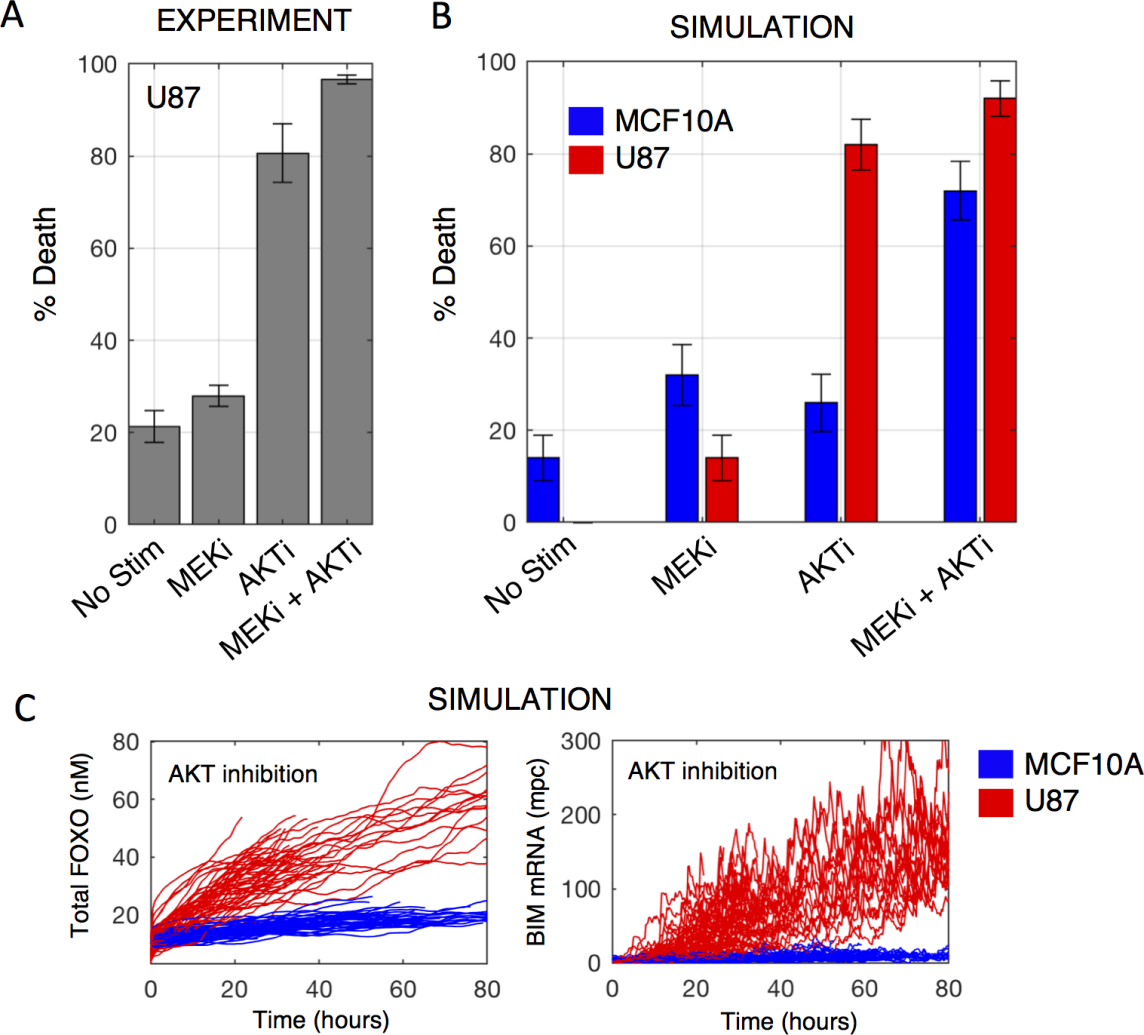
Predicting cell death responses in U87 cells. A) Serum-starved U87 cells were treated as indicated (MEKi-10μM; AKTi-10μM) for 48 hours and percent of cell death was measured via flow cytometry. B) Simulation results comparing cell death responses of a U87-tailored (red) and MCF10A-tailored (blue) model. C) Single cell traces comparing U87 (red) to MCF10A (blue) total FOXO (nuclear + cytoplasmic forms; left) and BIM mRNA levels (right).

Thus far we have focused on tailoring the model to MCF10A cells, and have parameterized the model to reflect expression levels, protein dynamics, cell death and cell cycling percentages following various stimuli. Does our model possess the ability to be predictive of cell fate outcomes when tailored to an alternative cellular context? To test this, we used data from U87 cells, a glioma cell line. Flow cytometry experiments of U87 cells treated with a MEK inhibitor, an AKT inhibitor, or dual kinase inhibition indicated pronounced sensitivity of U87 cells to AKT, but not MEK, inhibition (Fig 8A); this result was not found for MCF10A cells, which were found to be minimally sensitive to either inhibitor used alone (Fig 5A, grey bars). We thus sought to test whether the model tailored to U87 expression data could predict this increased sensitivity to AKT inhibition.

To test this, we quantified mRNA levels in serum-starved U87 cells and tailored the model with the same setup pipeline procedure as for the MCF10A-tailored model (Fig 2D). In addition, because U87 cells are PTEN-deficient [55–57], we set the PTEN translation rate constant to zero, essentially removing PTEN from the system. U87 cells are also thought to possess a mutation in CDKN2A; however, since the incorporation of this mutation would negligibly affect cell death responses to kinase inhibition, we excluded it from this analysis. However, future iterations of this model application to U87 should certainly incorporate CDKN2A mutation, in addition to more generally capturing the spectrum of mutations in this and other cell lines. We then simulated our U87 cell model under the above experimental conditions, recording percentage of cell death at 48 hours post stimulus (Fig 8B, red bars) and compared this to flow cytometry data (Fig 8A). Remarkably, the U87-tailored model predicted with reasonable accuracy cell death responses to the different stimuli, capturing the increased sensitivity to AKT inhibition compared to our MCF10A-tailored model simulations. We noted that the U87-tailored model is unable to capture basal cell death under the “No Stim” condition (Fig 8B). We suspect this highlights a limitation in the current initialization procedure, specifically in the tuning of basal caspase 8 activity to reflect balanced levels of intrinsic death signals in the absence of any stimuli. Future work must more carefully consider the phenotypic requirements of initialization across cell types. Simulated time courses suggest that the increased sensitivity to AKT inhibition in U87 cells may be driven by increased accumulation of FOXO (Fig 8C, left), and subsequently the pro-apoptotic BIM (Fig 8C, right), when compared to MCF10A cells. A proposed model-derived mechanism behind this observation lies in the faster degradation rate of phosphorylated FOXO [58–60]. In U87 cells, the presence of the PTEN-null mutation, in addition to higher basal AKT levels, results in higher basal AKT activity. This results in increased FOXO phosphorylation, which causes it to be more rapidly degraded. In turn, when AKT is inhibited and FOXO becomes unphosphorylated, its degradation is slowed and it accumulates, driving BIM upregulation and cell death. Future experimental work will have to test these mechanistic interpretations.

## Discussion

We developed the first pan-cancer driver network model of cell proliferation and death regulation that has a biological context mechanistically parameterized by multi-omics data. The model describes fractional cell kill and partial cytostasis in response to treatment with a variety of anti-cancer drugs, conditioned upon an expression and microenvironmental context. We first developed sets of unit test operations that build confidence in individual submodels, and then evaluated the ability of the model to reproduce cell biological behavior as an integrated whole, which we subsequently analyzed to gain insight into biochemical mechanisms of stochastic cell proliferation, death and transformation. We then applied the model to a glioma cell context, which was able to predict differential sensitivity to AKT inhibition.

While this model is complex and larger than any previous such signaling model, some may argue it still does not account for enough biology. We have predominantly focused on a single non-transformed cell line with no mutations. Impeding future work here is that most mutations are not functionally well-understood. We do not incorporate detailed mechanisms of post-translational Myc regulation, pathways outside of pan-cancer scope, tumor metabolism, hypoxia signaling or immune response. We do not include VEGFR, which although an important receptor tyrosine kinase in cancer, predominantly plays a tissue level role in angiogenesis as opposed to a signaling role inside tumor cells themselves, the focus of this study [61,62]. We also have not considered cell-to-cell communication from physical contact, autocrine, or paracrine signaling—so called population effects—which will be important to consider moving forward. In our experiments, we minimized cell confluence to reduce such effects. Nevertheless, MCF10A are strongly adherent to one another, and the presented data, results and phenotypes, and therefore our model implicitly account for these population effects. Future experiments may focus on better teasing these components apart. Yet others may argue that our first model draft is too large to be useful. Symmetric critiques often reflect striking an appropriate balance, but both views have validity. Rule-based modeling [63] and algorithmic model building [64] could help modelers capture more biology. Open and FAIR [65] data that is well-annotated and available by computational query (e.g. LINCS consortium) will be needed to drive such modeling forward.

We certainly do not claim that our present model is unique, neither in terms of structure nor parameterization. Several variants of submodels exist [22,29,30,66], as well as efforts to integrate them with expression data [67–70], most notable the whole cell model of mycoplasma [71]. These classes of models are well-known to exhibit parametric non-identifiability but yet are still able to make valid and precise biological predictions [72]. The fact that these models stand on the shoulders of decades of cell and molecular biology research and are formulated to the extent possible based on physicochemical principles facilitates predictive potential despite the inherent uncertainty. Increased ability to algorithmically compare model structure and predictions to a wide net of publicly available experimental data will help reduce model uncertainty. Regardless, model complexity is driven by the questions and data at hand, and here the ultimate goal of cancer precision medicine coupled with omics data for tailoring gives a rational basis for the model size and complexity. The fact that when we tuned the model to the U87 glioma cell context, it could predict increased sensitivity of cell death to AKT inhibition, suggests that the level of detail may be an appropriate foundation.

To study stochastic cell fate with our model we combined penalized regression (identify predictive features) with machine learning (relate predictive features to cell fates). Like prior work [12], we found that ERK+AKT inhibitor-induced apoptosis was not predictable from initial protein levels. Rather, the couplet of time-averaged total BIM and BCL2 levels following death stimulus were highly predictive. Thus, apoptosis seems inherently unpredictable at the time of the death stimulus, and rather evolves stochastically over the treatment time course. It is difficult to prove such a claim conclusively, as a combination of different initial measurements with non-linear models could provide such predictive power. However, the results do suggest a strong stochastic element of cell death that is hard to predict from initial protein levels alone.

To test the BIM and BCL2 dynamics predictions experimentally, the ability to track cells over time from the onset of perturbation to the final cell fate is needed. If one could engineer cell lines to express fluorescent protein tagged (yet still functional) copies of all BIM and BCL2 isoforms from each allele, and monitor their responses by live-cell imaging, then this prediction could be tested. However, construction of such lines remains a challenging proposition.

In contrast to the inherent unpredictability of apoptosis based on initial protein levels, S-phase entry in simulations was predictable from initial C-Raf and B-Raf levels. Why is this the case for S-phase entry and not apoptosis? There are several potential factors in the model. First is the relatively low expression levels of both Raf isoforms, giving larger expression noise. Second is the bottleneck role of Raf in propagating signals from RTKs to S-phase entry, coupled with enzymatic signal amplification as opposed to stoichiometric titration as in apoptosis signaling. Third is the predominant stochastic control of S-phase entry by ERK signaling dynamics, as opposed to AKT signaling dynamics. This may be due in part to the downstream positive feedback of cJun expression, which amplifies noise. Small fluctuations in Raf levels therefore might be amplified to all or nothing cJun and consequently cell cycle entry responses.

Our model can naturally account for drug dose, promiscuity, scheduling, kinetics and pharmacodynamics. These hallmarks of pharmacology are as yet difficult to rationalize with genomic approaches. One potential application of ours and similar models is improving upon genomic-based approaches to cancer precision medicine. For example, patient transcriptomic data could be used to tailor the model as described here, giving an *in silico* test bed to explore a plethora of single or combination therapy options, including dosing and timing. Given advances in patient-derived experimental systems from biopsies [73], perturbation data can be used to further refine patient-specific mechanistic models and test drug predictions prior to clinical administration. Such predictions would have engrained within important properties of clinical response such as the promiscuity of many targeted kinase inhibitors [74] and dynamic feedback mechanisms in cancer cells important for resistance [75]. Moreover, given inference of subclonal evolution patterns of a patient’s tumor [11], multiple instantiations of the model could be generated to find a cross-section of therapies, or perhaps even order of therapies, that could most efficiently control an (epi)genomically heterogeneous tumor.

Alternatively, virtual clinical trials could be conducted for a particular drug or combination, given a cohort of patients defined by multi-omics data (e.g. transcriptomics). Simulation results could stratify patients for inclusion or exclusion in trials—an exceedingly important task given the niche efficacy of targeted drugs from single genetic biomarkers. Further simulations could optimize dosing and timing strategies. With now hundreds of FDA-approved anti-cancer therapeutics (including dozens of targeted drugs), and the realization that combination therapy is almost always required for clinical response, computational approaches such as this that help prioritize drugs, their doses, and their scheduling for particular patients will become increasingly important in drug development and personalized oncology.

## Methods

### Overview

Our model is the amalgamation of several already-published models from the literature into an integrated system of ordinary differential equations (ODEs). The model can be subdivided into six principal submodels: (1) receptor tyrosine kinase (RTK) [22,23,76–78], (2) proliferation and growth (which includes Ras-Raf-MAPK plus PI3K-AKT-mTOR pathways) [23,29,30], (3) cell cycle [40], (4) apoptosis [37], (5) DNA damage response [35], and (6) gene expression (the genes/proteins involved in each submodel are noted in main text Fig 1). The model is composed of ~1200 total species, which includes all genes, mRNAs, lipids, proteins, and post-translationally modified protein/protein complexes. There are a total of 141 genes across all submodels that can be reduced into 102 functionally unique proteins, which we term “protein conglomerates” (see S1 Table for mapping). These 102 immediate gene products can become post-translationally modified in various ways to make up the remaining ~1100 species in the model. We opted to represent the majority of reactions as elementary steps to the extent possible, using mass action kinetics. However, sometimes we employ Michaelis-Menten or Hill representations to capture events where the mechanisms are not sufficiently elucidated. Kinetic parameters are taken from original source models, previous models, experimental studies, or estimated to fit empirical observations (see S2 Table for rate laws and rate constant sources). See Supplementary Methods for a more detailed description of the model structure and formulation.

### Computational methods

#### Data and software availability

The MATLAB code used to run the model is available as supporting information (S1 Code).

#### Model implementation

The model was implemented and simulated using MATLAB R2017b. The model switches between an ordinary differential equation portion and stochastic gene expression portion with a 30 second time step. We used a MATLAB interface to CVODES (sundialsTB v.2.4.0), which greatly increased computational speed for the deterministically simulated model portion (ODEs). Most simulations were run on a MacBook Pro with a 2.5 GHz Intel Core i5 processor and 16GB of RAM.

#### Initial concentrations

In order to tailor the model to the expression context of MCF10A cells, we acquired gene copy number information from a literature source [21], mRNA expression levels by performing RNA-seq, and protein expression levels by performing mass spectrometry-based proteomics. We acquired RNA-seq and proteomics data on serum-starved cells. We used proteomics data to set initial protein levels for the RTK, proliferation and growth, and apoptosis submodels. For the majority of proteins in the cell cycle and DNA damage submodels, we used initial conditions from their original source models. One reason is that the serum-starved condition (at 24 hours) is non-cycling and low stress, with little relevant expression of such proteins. Another reason is because these submodels already included intrinsic synthesis and degradation reactions, which we largely did not alter. As exceptions to this rule, we set initial cyclin D, p21, BRCA2, MSH6, and MGMT using proteomics data, as these were either proteins we added to the submodels *de novo* (all except cyclin D) or served as a link between submodels (cyclin D). We acquired a value for initial ribosome level from the literature; we found a range of values from ~2 million to ~10 million copies per cell across mammalian cells [79], thus we estimated 6 million copies of ribosomes copies per cell as an intermediate value. Note this value is ~10-fold greater than the estimated number of mRNA molecules / cell, in line with the notion of the poly-ribosome (multiple ribosomes simultaneously translating the same transcript) and translation initiation being largely rate-limiting. The initial concentrations for all ligands and all post-translationally modified forms of proteins were initially set to zero. The levels of post-translationally modified forms of proteins were approximated during the initialization procedure (see Supplementary Methods for details).

#### Calculation of transcription and translation parameters

Each gene exhibits a unique transcription and translation rate, which we calculated from our RNA-seq and proteomics data. The general form of the deterministic differential equations describing transcription and translation are as follows:

$$\frac{dm}{dt}=k_{bm}∙g^{*}-k_{dm}∙m$$ (1)

$$\frac{dp}{dt}=k_{bp}∙m-k_{dp}∙p$$ (2)

Where g* is active gene levels, *m* is mRNA levels, *p* is protein levels, *k*_*bm*_ is the transcription rate constant, *k*_*bp*_ is the translation rate constant, *k*_*dm*_ is the mRNA degradation rate constant, and *k*_*dp*_ is the protein degradation rate constant. At steady state, the transcription and translation rate constants are given by

$$k_{bm}=\frac{k_{dm}∙m_{ss}}{{g^{*}}_{ss}}$$ (3)

$$k_{bp}=\frac{k_{dp}∙p_{ss}}{m_{ss}}$$ (4)

The mRNA and protein degradation rate constants were calculated from gene-specific half-life measurements observed in data from [80]. Measurements from Schwanhausser et al. [80] were generated in NIH-3T3 cells, a murine fibroblast cell line; therefore, how do we know these mRNA and protein half-lives will apply to MCF10A human breast epithelial cells? First, observations point to consistent gene-specific mRNA-to-protein ratios across different human cell types [81], which, we reason, supports the conservation of protein half-lives across distinct cell types. Second, previous studies have reported conservation of mRNA and protein half-lives across species [82,80]. These reports give us confidence that mRNA and protein half-lives taken from Schwannhausser et al. [80] and other mammalian cell sources serve as reasonable estimates for half-life estimates in our MCF10A context.

We quantified the levels of mRNA and protein at steady-state (*m*_*ss*_ and *p*_*ss*_, respectively) based on our RNA-seq and proteomics measurements. The amount of active gene product at steady-state (g*_ss_) was derived. The differential equation for active gene production over time, given summation over a large population of cells, is defined as:

$$\frac{{dg}^{*}}{dt}=k_{gac}∙g-k_{gin}∙g^{*}$$ (5)

Applying moiety conservation to gene amount yields the following steady-state condition

$${g^{*}}_{ss}=\frac{k_{gac}∙g_{total}}{\left(k_{gin}+k_{gac}\right)}$$ (6)

Gene activation and inactivation rate constants were obtained from the literature [15] (see Supplementary Methods for more details). As mentioned above, the total amount of gene copies for each gene (*g*_*total*_) was also obtained from a literature source [21].

#### Steady-states

There are two relevant steady-states in our computations. The first is post-initialization. Here, the model is deterministic. It has expression and phenotypic components, and corresponds to a cell in a serum-starved state that is (i) not cycling, (ii) not undergoing apoptosis, and (iii) has mRNA and protein levels that match experimental measurements. With regards to cell size and ribosome number, they are constant at this steady-state. The concentrations of all molecular species in the model, in a deterministic setting, are time invariant at this steady-state as well. This corresponds to a single average cell.

The second is prior to treatment. The conditions from the above steady-state are used and then stochastic gene expression is turned on for 24 hours (see below section). This results in a cell with protein levels that have changed from the average cell and as compared to other such simulated cells.

#### Creating a simulated cell population in silico

In an experimental in vitro setting, cells on a dish exhibit cell-to-cell variability in mRNA and protein expression levels. This natural variability is largely due to stochastic gene expression processes and can in some cases lead to cell-to-cell differences in phenotypic response to a perturbation. We desired to mimic this natural variability in our computational simulations. Because the model begins with the same initial conditions for each cell, we first run a stochastic simulation for each cell for 24 hours in the absence of external stimuli prior to performing any in silico experiment. This initialization step introduces a natural amount of variation in protein and mRNA levels across the simulated cell population, which randomizes the serum-starved condition in subsequent simulations (see Fig 2).

#### Lasso regression and support vector machine (SVM) training

As part of our analyses we performed two lasso regressions; one related to apoptosis and one related to cell cycle. We used lasso regression to reduce our set of predictors for the SVM training. For apoptosis, we desired to identify genes that were predictive of whether a cell would die early (before 40 hours) or late (after 40 hours) in response to dual MEK and AKT inhibition. We used the lasso function in MATLAB to perform the regression. As predictor variables we used the total observable quantities of each protein (sum across all forms of a protein, i.e. conserved moiety), either at the initial time point, or averaged across a certain time frame (8 or 40 hours). Our response variable was binary (whether or not the cell died before 40 hours). We used the default settings and set the “CV” parameter (fold cross validation) to 5. Top hits were identified for mean 40 hours (Bcl2, BIM, EGFR, ErbB2, ErbB3), mean 8 hours (Bcl2, BIM, BAD, PDK1, FOXO), and initial levels (Bcl2, BIM, PARP, PDK1, FOXO). We chose to focus on time-averaged Bim and Bcl2 as they consistently appeared in all groups and no difference was found when comparing model predictability when other predictors were included or not. We then trained a binary SVM classifier using the function fitcsvm in MATLAB, using only time-averaged Bim and Bcl2 total protein levels as predictor variables and early/late death responses as the response variables. We set the “Standardize” option to “true”, which normalizes the predictor data. We used a total of 200 cells for training and another 200 for testing (for the lasso regression, above, we used the 200 training set as well). We tested the model using the predict function and generated ROC curves using the perfcurve function in MATLAB. For the cell cycle analysis, we performed the same steps as above though only considered the initial concentrations of each protein as predictors. Our response variable was binary depending on whether the cell divided within the 30-hour time course or not. CRaf and BRaf were the top two hits from the lasso regression, which we then used to train a binary SVM classifier, and generate ROC curves as above.

#### Sensitivity analysis on degradation parameters

Parameter sets for degradation rate constants were generated using the lhsnorm (latin hypercube sample with normal distribution) function in MATLAB, with means equal to the original parameter and a standard deviation of 3.16 (assuming no covariance between parameters). This introduced variability of ~2 to 4-fold from the original values. The model was re-initialized for each parameter set; some sets did not meet the initialization criteria and were discarded. A random population of 25 cells was created for each parameter set (24 hours simulation) and percentage of cell death was recorded 24, 48, and 72 hours post EGF (20ng/mL) + insulin (10μg/mL) + MEK inhibitor (10μM) + AKT inhibitor (10μM) simulated treatment.

#### Tailoring model to U87 cell data

To tailor the model to U87 data, mRNA molecules per cell from MCF10A cells were switched out for mRNA molecules per cell from serum-starved U87 cells (experiments and quantification performed the same as for MCF10A cells). As U87 cells are PTEN-deficient [55–57], we set its translation rate constants to zero, essentially removing it from the system. All remaining parameters were kept the same as the MCF10A-tailored and trained model. The U87-tailored model was initialized (see Fig 2D and Chapter 3 in S1 Supplemental Methods) and run in exactly the same manner as for the MCF10A-tailored model. We used gene-specific protein-to-mRNA ratios from MCF10A cells.

#### Significance testing

Significance testing was performed by using the ttest2 function in Matlab. Three stars indicate a p-value less than 0.001, two stars indicate a p-value in-between 0.001 and 0.01, and one star indicates a p-value between 0.01 and 0.05. Error bars correspond to standard error of the mean where possible.

#### Flow cytometry software

Analysis of flow cytometry data was performed using Cytobank. Raw flow cytometry data is available at flowrepository.org, accessions FR-FCM-ZYGG, FR-FCM-ZYHQ, FR-FCM-ZYHP, FR-FCM-ZYHX, and FR-FCM-ZYGF.

### Experimental methods

#### Cell culture

MCF10A cells were cultured in DMEM/F12 (Gibco; Cat: 11330032) medium supplemented with 5% (v/v) horse serum (Gibco; Cat: 16050–122), 20ng/mL EGF (PeproTech, Cat: AF-100-15), 0.5 mg/mL hydrocortisone (Sigma, Cat: H-0888), 10μg/mL insulin (Sigma, Cat: I-1882), 100ng/mL cholera toxin (Sigma, Cat: C-8052), 2mM L-Glutamine (Corning; Cat: 25-005-CI) and 1X (10,000 I.U./mL / 10,000 ug/mL) penicillin/streptomycin (Corning; Cat: 30-002-CI). Cells were cultured at 37°C in 5% CO_2_ in a humidified incubator, and passaged every 2–3 days with 0.25% trypsin (Corning; Cat: 25-053-CI) to maintain subconfluency. Cells originate from a female host, were a gift (Gordon Mills and Yoko Irie) and we did not independently authenticate the cells. Starvation medium was DMEM/F12 medium supplemented with 2mM L-Glutamine and 1X penicillin/streptomycin.

#### RNA sequencing and analysis

MCF10A cells were seeded at ~30% density (1 million cells / 60mm diameter dish, one dish per biological replicate), and incubated overnight in full growth medium. The next morning, the full growth medium was aspirated, cells were washed once with PBS, and then placed in starvation medium for 24 hours. Total RNA was extracted using TRIzol (Life Technologies, Cat: 15596018) per manufacturer instructions (detailed SOP at www.dtoxs.org; DToxS SOP A– 1.0: Total RNA Isolation). RNA sequencing and analysis was performed as previously described [83]. We prepared RNA-seq libraries by adapting a single-cell RNA-seq technique—Single Cell RNA Barcoding and Sequencing method (SCRB-seq; [84,85])—to extract total RNA from cells (detailed SOP at www.dtoxs.org; DToxS SOP A– 6.0: High-throughput mRNA Seq Library Construction for 3' Digital Gene Expression (DGE)). This method involves converting each Poly(A)+ mRNA molecule to cDNA labeled with universal adaptors, sample-specific barcodes, and unique molecular identifiers (UMIs) on every transcript molecule using a template-switching reverse transcriptase. cDNA was amplified and prepared for multiplexed sequencing, enriching for 3’ ends preserving strand information by using a modified transposon-based fragmentation approach. Libraries were sequenced using the Illumina HiSeq 2500 platform (detailed SOP at www.dtoxs.org; DToxS SOP A– 7.0: Sequencing 3'-end Broad-Prepared mRNA Libraries). Analysis was performed using a custom Python script (detailed SOP at www.dtoxs.org; DToxS SOP CO– 3.1: Generation of Transcript Read Counts). In brief, sequenced reads were aligned to a 3’-focused hg19 reference using BWA, and counts of reads that aligned to one or more than one gene with high confidence were calculated. The UMI count is defined as the number of distinct unique molecular identifiers (UMI) sequences embedded in those aligned reads and is well-known to alleviate PCR bias that can distort linearity in quantification. Biological triplicates were performed, but one sample failed to be sequenced, leaving biological duplicates.

#### Proteomics and analysis

MCF10A cells were seeded at ~30% density (5 million cells / 150mm diameter dish, 2 dishes pooled per biological replicate) and incubated overnight in full growth medium. The next morning, the full growth media was aspirated, cells were washed once with PBS, and then placed in starvation medium for 24 hours. Cells were trypsinized for 10 minutes, resuspended in 1X PBS, and spun down at 500g for 5 minutes. Cell pellets were then snap frozen in liquid nitrogen and shipped to the Advanced Proteomics Center at New Jersey School of Medicine to perform mass spectrometry. The cell pellet was subjected to urea lysis buffer composed of 8 M urea, 100 mM TEAB, 1X protease inhibitors (complete, EDTA-free Protease Inhibitor Cocktail from Sigma, prepared according to manufacturer instructions) and 1X phosphatase inhibitors (PhosSTOP from Sigma, prepared according to manufacturer instructions). Protein (50.0 μg total) was resolved by one dimensional SDS-PAGE. The gel lane was divided into 24 equal size bands, followed by in-gel digestion with trypsin performed as in [86]. Peptides were fractionated by reverse phase chromatography and analyzed directly by LC/MS/MS on a Q Exactive Orbitrap mass spectrometer (funded in part by NIH grant NS046593, for the support of the Rutgers Neuroproteomics Core Facility). We used Maxquant to analyze the data and calculate iBAQ scores, using a human UniProt protein database as the reference proteome. Final proteins were identified with 1.0% false discovery rate (FDR) at both protein and peptide levels. This resulted in ~10k unique proteins. Biological triplicates were performed.

#### Calculation of mRNA and protein molecules per cell

Importantly, the units of the data acquired from the RNA-seq (UMI count; [84]) and mass spectrometry (iBAQ scores; [87]) technologies are linearly proportional to the number of mRNA or protein molecules in the original sample, respectively. To convert from these measurement units to molecules per cell, we first quantile normalized the three biological replicates using the MATLAB function, quantilenorm, with default settings. The quantile-normalized values were then converted from their original unit of measure to units of molecules per cell with the following logic.

Consider measurement of mRNA or protein *i*, *A*_*i*_ (in UMI counts or iBAQ score), and the corresponding absolute copy number per cell that gives rise to this measurement, *X*_*i*_. Due to the linearity of the measurement modality, we have:

$$A_{i}=\alpha X_{i}$$ (7)

Summing over all species *n* in the cell gives:

$$\sum_{i}^{n}A_{i}=\alpha \sum_{i}^{n}X_{i}$$ (8)

Dividing by these sums then yields the following proportionality to convert the measurement to absolute copy number per cell.

$$\frac{A_{i}}{\sum_{i}^{n}A_{i}}=\frac{X_{i}}{\sum_{i}^{n}X_{i}}$$ (9)

Using these equations requires an estimate for the total number of mRNA and protein molecules in a cell. The total number of mRNAs in a typical mammalian cell was estimated from three sources. The first was taken from [80] by summing all mRNA quantities found in their search (173,921 molecules per cell). The second was taken from the Molecular Biology of the Cell textbook [88], which gave a value of 360,000 molecules/cell. The third was calculated by converting the total picogram amount of RNA in a typical mammalian cell, estimated from one source to be 26pg [89], to molecules per cell given estimates that ~3% of total RNA is mRNA [79], that there are an average of 2000 nucleotides in a single mRNA transcript [79], and that the average molecular weight of a single nucleotide is 339.5 g/mol [79]; using this information we calculated a total 691,891 molecules per cell. We took the average of these three numbers and approximated the result to 400,000 molecules per cell. To approximate the total number of proteins in a typical mammalian cell we summed the total number of protein molecules found by Schwanhausser et al. [80], which amounted to 3.08x10^9^ protein molecules per cell. Importantly, our method for converting to molecules per cell resulted in reasonable agreement with data from Schwanhausser et al. [80]. When comparing the numbers between our dataset (“ours”) and the Schwanhausser dataset (“S”) for a handful of constitutively expressed proteins that typically do not significantly change across cell types, we find most of them to be within the same order of magnitude: MAP2K1 (ours: 147250; S: 284958), MAP2K2 (ours: 377540; S: 327073), MAPK1 (ours: 746463; S: 1400595), MAPK3 (ours: 76314; S: 195819), AKT1 (ours: 74762; S: 37060), and AKT2 (ours: 81476; S: 4325). Discrepancies here could be due to cell-type differences, however.

Other studies have integrated universal spike-in standards (e.g. [20]), which allow for a more direct experimental determination of absolute protein copy numbers per cell. Here we did not integrate such standards but rather used the method described above based on label-free quantification by iBAQ score. A subset of the published spike-in data was directly comparable with our data. The comparison demonstrates reasonable agreement (S2F Fig).

#### Micro-western experiments and analysis

MCF10A cells were seeded at ~30% confluence in 6-well plates (150,000 cells / well), incubated overnight in full growth medium. The next morning, the full growth medium was aspirated, cells were washed once with PBS, and then placed in starvation medium for 24 hours. Cells were treated with combinations of EGF and insulin at various doses for 5 minutes, 30 minutes, 3 hours, or 6 hours. Growth medium was aspirated fully and cells were lysed on ice with a custom-made lysis buffer tailored for micro-western applications composed of 240 mM Tris-Acetate, 1% SDS w/v, 0.5% glycerol v/v, 5mM EDTA, water, and various protease and phosphatase inhibitors—aprotinin (1μg/mL), leupeptin (1μg/mL), pepstatin (1μg/mL), β-glycerophosphate (10mM), sodium orthovanadate (1mM), and dithiothreitol (DTT; 50mM). Reagent sources are listed in our SOPs (www.birtwistlelab.com/protocols). Samples were boiled at 95 degrees for 5 minutes, sonicated using a VialTweeter (Hielscher), then concentrated ~5 to 10-fold using Amicon Ultra centrifugal filters (Merck Millipore Ltd). Total protein content was assayed via the Pierce 660 assay per the manufacturer’s instructions. Samples were printed in 24 “blots” onto a single 9.5% poly-acrylamide gel using a GeSim Piezoelectric Nanoplotter, followed by horizontal electrophoresis and wet transfer to a nitrocellulose membrane. The membrane was blocked with Odyssey Blocking Buffer (TBS; LICOR) then put into a 24-well gasket system that corresponds to the original lysate printing and horizontal running pattern. This gasket system allows different primary antibodies to be probed in each well. We probed using the following antibodies from Cell Signaling: pERK (Cat: 4370; 1:1000), pAKT (Cat: 4060; 1:2000), pEIF4E-BP1 (Cat: 2855; 1:500), cyclin D (Cat: 2978; 1:500) and α-Tubulin (Cat: 3873; 1:2000). We then incubated the entire gel with two secondary antibodies from LiCor: IR dye 800CW goat anti-rabbit (green; Cat: 926–32211) and IR dye 680CD goat anti-mouse (red; Cat: 926–68070) at 1:20,000. The membrane was then imaged via a LICOR Odyssey scanner and quantified with Image Studio Lite software as “signal” (intensity with background subtracted). For full SOPs, please visit www.birtwistlelab.com/protocols. Each signal (pERK, pAKT, pEIF4E-BP1, and cyclin D) was first divided by the respective α-Tubulin signal (loading control) in the same lane to normalize for lane-to-lane differences in loading. Then to normalize for any systematic inter-well differences, each ratio was then normalized to the serum-starved control lane in its well. Raw images can be found in S4 Fig.

#### BrdU incorporation/propidium iodide flow cytometry experiments and analysis

MCF10A cells were seeded at ~1 million cells in a 10cm dish, and incubated overnight in full growth medium. The next morning, the full growth media was aspirated, cells were washed once with PBS, and then placed in starvation medium for 24 hours. Cells were treated with indicated doses of EGF (10nM), insulin (10μg/mL), the combination dose (10nM EGF + 10μg/mL insulin), and 30 min pre-incubation with a MEK inhibitor (PD0325901, Sigma, Cat: PZ0162) or AKT inhibitor (MK2206, ChemieTek, Cat: CT-MK2206) as indicated (both at 10μM). An hour before lifting, cells were treated with a pulse of 10 μM BrdU (Sigma). Cells were trypsinized, washed with 4°C 1X PBS and pelleted by centrifugation at 1000rpm for 5 minutes. To fix cells, they were resuspended in 200uL 4°C 1X PBS and 2mL of 4°C 70% ethanol (v/v in ddH_2_0) were added dropwise while vortexing at low speed. The cells were then stored at 4°C for at least 30 minutes. Cells were pelleted at 1000rpm for 5 minutes and resuspended in 1mL PBS. We added 1mL of 4N HCl and incubated the cells at room temperature for 15 minutes. We washed the cells by centrifuging at 1000rpm for 5 minutes and resuspending in 1mL PBS. After another spin, we resuspended the cells in 1mL PBT (PBS + 0.5% BSA (g/L) + 0.1% Tween 20 (v/v) (Sigma)). We spun the cells down and resuspended in 200 μL of PBT containing 1/40 dilution of anti-BrdU antibody (BD Biosciences) and incubated at room temperature for 30 minutes. We then added 1mL of PBT, spun the cells down and resuspended in 200uL PBT containing 1/40 dilution of anti-mouse FITC conjugate (BD Biosciences); we incubated cells in this for 30 minutes at room temperature in the dark. We added 1mL of PBT, spun cells down and resuspended in 250 μL PBS containing 250μg/mL RNAseA (Qiagen MaxiPrep Kit) and 10μg/mL propidium iodide (Sigma). We incubated the cells at room temperature for 30 minutes in the dark prior to analysis by flow cytometry on an Accuri C6 (BD Biosciences). We first gated for non-debris cells (FSC-A vs. SSC-A), then gated for singlets (SSC-A vs. SSC-H), then plotted PI intensity (x-axis) versus BrdU intensity (y-axis). We quantified percentages of cells in G1/G0 phase (First PI peak, BrdU negative), S-phase (BrdU positive), and G2/M phase (2^nd^ PI peak, BrdU negative). Our metric of percent cell cycling was the S-phase population plus the G2/M population percentages. Raw data are available at flowrepository.org.

#### Annexin V/Propidium iodide flow cytometry experiments and analysis

MCF10A cells were seeded at ~30% confluence in 12-well plates (75,000 cells / well), and incubated overnight in full growth medium. The full growth medium was replaced by starvation medium for 24 hours. Cells were then treated with different growth factor-inhibitor combinations as indicated and cells were assayed at 24, 48, and 72 hours post stimulation. A MEK inhibitor (PD0325901, Sigma, Cat: PZ0162) and/or AKT inhibitor (MK2206, ChemieTek, Cat: CT-MK2206) was used at 10μM. Anisomycin (Sigma, Cat: A9789), an inhibitor of protein and DNA synthesis, used at 10μM served as a positive control for cell death (death percentages were always greater than 90% cell death). Cells in full growth medium without treatment served as a negative control (death percentages were always less than 10% cell death). Cells were trypsinized, resuspended in PBS and pelleted by centrifugation at 1100g (to ensure collection of dead cells) for 5 minutes at room temperature. Cells were then resuspended in 100uL of Annexin-V binding buffer (10mM Hepes, 140mM NaCl, 2.5mM CaCl_2_ (Sigma) in ddH_2_0 pH 7.4) with 5uL of AnnexinV-FITC (BioLegend, Cat: 640906) and 0.1μg/mL propidium iodide (Sigma). Cells were incubated for 20 minutes at room temperature prior to analysis by flow cytometry. In TRAIL experiments, cells in full growth medium were treated with multiple doses of superkiller TRAIL (tumor necrosis factor (TNF)-related apoptosis-inducing ligand) (Enzo Life Sciences, Cat: ALZ-201-115-C010). After 5-hour TRAIL treatment, cells were harvested and analyzed by flow cytometry as described above, and a dose-response curve of two-fold serial dilutions of TRAIL ranging 2-300ng/mL was plotted. We first gated for non-debris cells, then gated for singlets (both as above), then quantified the % of cells in the Annexin-V positive population, which was our metric for percent cell death. Raw data are available at flowrepository.org.

#### Etoposide flow cytometry experiments

MCF10A cells were seeded in full growth medium at approximately 30% confluence in 12-well plates (75,000 cells / well) and incubated overnight. Cells in full growth medium were then treated with 100 μM of Etoposide (Cayman Chemical, Cat: 12092) and assayed at 24, 48, and 72 hours post stimulation. Alternatively, after the cells attached overnight, the full growth medium was aspirated, cells were washed once with PBS, and then placed in starvation medium for 24 hours. These cells were then treated with 100 μM Etoposide and assayed at 24 and 48 hours post stimulation. Again, Anisomycin (10μM; Sigma, Cat: A9789) was used as a positive control for cell death and cells in full growth medium without treatment were used as a negative control for cell death. Cells were assayed with Annexin V-FITC and PI and analyzed as described above for the apoptosis flow cytometry experiments. Raw data are available at flowrepository.org.

#### Single-cell microscopy experiments

Kinase translocation reporters (KTR) are fluorescent protein-linked kinase translocation biosensors that shuttle between the nucleus and cytoplasm based on the activation state of the pathway of interest. For this ERK KTR construct, the probe is localized to the nucleus when inactive (via NLS) and shuttled to the cytoplasm when active/phosphorylated by ERK (via NES). Signaling dynamics can be determined by plotting the ratio of cytoplasmic-to-nuclear KTR fluorescence [32].

The ERK KTR clover probe (pLenti CMV HYGRO DEST ERK KTR Clover; hygromycin resistance) was generated using gateway cloning enzymes BP, LR clonase, and the source vector pLenti CMV Puro DEST ERK KTR Clover. Lentivirus was generated as previously described [90] for both this ERK KTR clover probe and a pNLS-iRFP713 construct (used as nuclear marker). Lentiviral supernatant was collected and concentrated via Amicon Ultra-15 filters NMW 100 kD centrifugal filters.

MCF10A cells were transduced with ERK KTR concentrated lentiviral supernatant. Twenty-four hours post transduction, cells were imaged to confirm expression of ERK KTR clover. Cells expressing ERK KTR clover were sparsely seeded on a 150x25mm dish and cultured in selection media, DMEM F12, 5% Horse serum, EGF (20ng/mL), Hydrocortisone (.5μg/mL), Cholera toxin (100ng/mL), Insulin (10μg/mL), and hygromycin (31.25μg/mL) and placed in a humidified incubator at 37°C, 5% CO2. Cells were cultured until colonies were visible under brightfield microscope. Individual colonies were harvested by trypsonization (.25%), re-plated and imaged to verify expression of ERK KTR clover. Colonies showing uniform expression of ERK KTR were used in subsequent probe validation.

MCF10A cells were seeded at ~30% confluence (5,000 cells/well) in 96-well plates (Corning 3603) and incubated overnight in full growth medium (DMEM F12, 5% Horse serum, EGF (20ng/mL), Hydrocortisone (.5μg/mL), Cholera toxin (100ng/mL), Insulin (10μg/mL), and hygromycin (1.5μg/mL)). These cells were then transduced with the pNLS-iRFP713a nuclear marker (used for image segmentation purposes). The following day, the full growth medium was replaced by starvation medium for 24 hours. We then treated cells with EGF (20ng/mL) plus insulin (10μg/mL) and quantified ERK KTR clover response over time at 3 minute intervals over the course of 1 hour. We compared this to mock treatment (no treatment; serum-starved) and cells pre-treated with a MEK inhibitor (PD0325901; 100nM) for 30 minutes following stimulation with EGF plus insulin. Baseline ERK KTR activity was acquired for each condition for 15 minutes. Images were acquired with the InCell2200 (incubated at 37°C with CO2, Nikon 20X/0.75, Plan Apo, CFI/60, 3 fields/condition, KTR Ex. 475/28 Em. 511.5/23, iRFP Ex. 632/22, Em. 684/25, brightfield). Images were analyzed using a custom CellProfiler pipeline [91], segmenting cytoplasmic and nuclear compartments for each cell. Baseline normalized cytoplasmic-to-nuclear KTR fluorescence was plotted for each segmented cell. The CellProfiler pipeline can be supplied when requested.

#### Additional resources

A full SOP for the μ-Western blot procedures can be found at www.birtwistlelab.com/protocols. More detailed SOPs for RNA isolation (DToxS SOP A– 1.0: Total RNA Isolation), RNA-seq library preparation (DToxS SOP A– 6.0: High-throughput mRNA Seq Library Construction for 3' Digital Gene Expression (DGE)), RNA sequencing (DToxS SOP A– 7.0: Sequencing 3'-end Broad-Prepared mRNA Libraries), and analysis (DToxS SOP CO– 3.1: Generation of Transcript Read Counts) can be found at www.dtoxs.org.

## Supporting information

S1 Fig Detailed kinetic scheme of model. This kinetic scheme depicts all of the reactions in the model. Labels correspond to the indices for each rate law in the equations and code. A “p” prior to a protein name indicates it is phosphorylated. An underscore between two proteins indicates they are in complex. An arrow pointing to a curved arrow indicates three reactions: association, dissociation, and catalysis. A dashed arrow indicates a lack overt mechanism, described with either Hill or Michaelis-Menten rate laws.
(TIF)

S2 Fig Properties of the expression submodel. A) Probability of zero (blue diamonds), one (red squares), or two (green triangles) gene switching events given different time step intervals between the stochastic and deterministic components of the model. We chose 30 seconds as a tradeoff. B) Cell-to-cell variability in the timing of the first cell division event following growth factor stimulation. Different time steps between the stochastic and deterministic components of the model are shown along the x-axis. Growth factor stimulation is with all growth factors set to 10nM, except for insulin, which was set to 1721nM. Variability seems rather consistent for the 6, 30, and 60 second time steps, though it collapses when the time step reaches 300 seconds. C) The solution to the hill function (theta) describing the effect of EIF4E on translation rate. D) The number of ribosomes in a single cell doubles during the course of a typical cell cycle duration (~20 hours) in response a full mitogenic stimulus (same as B). E) Correlation between protein levels across a population of cells, showing the typically observed global positive correlations. F) Correlation between our protein quantification and that from Shi et al. [20] in MCF10A cells (genes: ADAM17, ARAF, CBL, DUSP4, EGFR, ERRFI1, GAB1, GRB2, HRAS, KRAS, MAP2K1, MAP2K2, MAPK1, MAPK3, NRAS, PTPN11, PTPRE, RAF1, RASA1, SHC1, SOS1, SOS2). Line is x = y line.
(TIF)

S3 Fig Additional unit testing. A) Cooperativity profiles comparing the ideal case (left)—when only the receptors that bind each ligand are present on the cell surface and at appreciable levels—to those for the model tailored to expression levels for MCF10A cells (right). B) Time course plot showing dynamics of active plasma membrane-bound EGFR dimers (solid black line) and internalized receptors (dashed black line) in response to 10nM EGF, showing kinetics largely consistent with prior knowledge on trafficking and downregulation. C) Dose response curves for ppERK and ppAKT in the MCF10A-tailored model in response to various ligands at doses ranging from 1μM to 10^-3^nM. D) Data obtained from the LINCS (Library of Integrated Network Cellular Signatures) project [25] showing average (across 10, 30, and 90 minute time points) levels of phosphorylated ERK (dark grey) and AKT (light grey) in response to ligands (all at 100ng/mL) depicted in model. Relative activation amplitudes are comparable to model predictions from S3C Fig, although IGF induces more ERK activation than predicted by our model. E) Dynamic data from the stochastic simulations that comprise Fig 3F (different cells are different colors). DNA damage increases from top to bottom. Although the number of pulses increases with increasing DNA damage, pulse height and width remain relatively constant.
(TIF)

S4 Fig μ-Western blot raw images. A) Schematic outlining layout of conditions on μ-Western blot for every well. Concentrations are in units of nM. B) Raw scans of μ-Western blot membranes probed for pERK, pAKT, pEIF4E-BP1, α-Tubulin, and cyclin D (see Methods). Time points go from top-to-bottom. Treatment conditions go from left-to-right, as indicated in (A). Each row has biological replicates (three for pERK and pAkt, two for Cyclin D and p4EBP1).
(TIF)

S5 Fig Comparing experimental and simulated cell-to-cell heterogeneity. A) Left: Single cell traces of the ratio of cytoplasmic to nuclear fluorescence for ERK KTR probe (reporter of ERK kinase activity) stably expressed by MCF10A cells. Serum-starved cells were stimulated with EGF (20ng/mL) + insulin (10μg/mL). Right: Simulation results showing phosphorylated ERK levels over time stimulated with same as in left. B) Serum-starved and MEK inhibitor (10μM) controls for ERK KTR probe showing no probe activity.
(TIF)

S6 Fig Additional apoptosis integrated unit testing. A) Simulated cells are treated with low doses of TRAIL (1ng/mL and 0.1ng/mL) and plots show the cumulative sum of BIM and Bcl2 levels across the time course during which they are alive (analogous to Fig 5F). Cell trajectories are colored in terms of their time to death. Here we see no obvious relationship between BIM and Bcl2 that is dependent on time to death as we saw in Fig 5F, highlighting to the stimulus-specific nature of apoptosis sensitivity. B) Sensitivity of cell death responses (stimulated with EGF + insulin + MEKi + AKTi) to protein half-life was tested by running 53 randomly sampled parameter sets. Degradation rates were altered by a maximum of 2–4 fold from original value (see Methods). Model was re-initialized with each parameter set and the percentage of dead cells at 24, 48, and 72 hours was measured (colored lines indicate different parameter sets). Box and whisker plot quantifies variability between parameter sets (red bar is the median).
(TIF)

S7 Fig Time-averaged ERK and AKT activities in simulations. A) The average, time-integrated ppAKT levels across different types of stimuli—EGF (10nM) + insulin (10μg/mL), EGF (10nM) alone, EGF (10nM) + insulin (10μg/mL) + small AKTi dose (1nM), or EGF (10nM) + insulin (10μg/mL) + small AKTi dose (2nM). A decrease in ppAKT is observed. B) The average, time-integrated ppERK levels across the same stimuli as in (A). No decrease in ppERK is observed. C) The percentage of proliferating cells at 24 hours post stimulation reveals that diminishing amounts of ppAKT signal results in a decrease in the percentage of proliferating cells. D) The average, time-integrated ppERK levels for cycling cells reveals no relationship between ppERK levels in cycling cells and ppAKT levels. This indicates that higher AKT activity does not lower the threshold of ERK activity needed to cause cell cycling.
(TIF)

S1 Supplemental Methods Further extrapolations and details on the inner workings of the model and how each submodel or algorithm was implemented. (PDF)

S1 Table Gene expression parameters. Expression levels, transcription rates, translation rates, mRNA and protein half-lives, and data sources for each gene in the model.
(XLSX)

S2 Table Reactions and parameters for submodels. Rate equations, reaction descriptions, parameter values, and parameter sources for each submodel.
(XLSX)

S1 Code Matlab model code. Matlab code needed to run the model, run all simulations included in this paper, and generate all figures.
(ZIP)
