# Correction: A mechanistic pan-cancer pathway model informed by multi-omics data interprets stochastic cell fate responses to drugs and mitogens

**DOI:** 10.1371/journal.pcbi.1006189

**Published:** 2018-07-09

**Authors:** The PLOS Computational Biology Staff

The supporting information item S1 Code is incomplete.

The final, peer-reviewed version of S1 Code can be viewed here:

The publisher apologizes for this error.

## Supporting information

S1 Code Matlab model code. (ZIP)
