# Supplementary figures and images for: A mechanistic pan-cancer pathway model informed by multi-omics data interprets stochastic cell fate responses to drugs and mitogens
Source: PLoS Comput Biol. 2018 Mar 26;14(3):e1005985. doi: 10.1371/journal.pcbi.1005985 (PMC5886578; doi:10.1371/journal.pcbi.1005985)

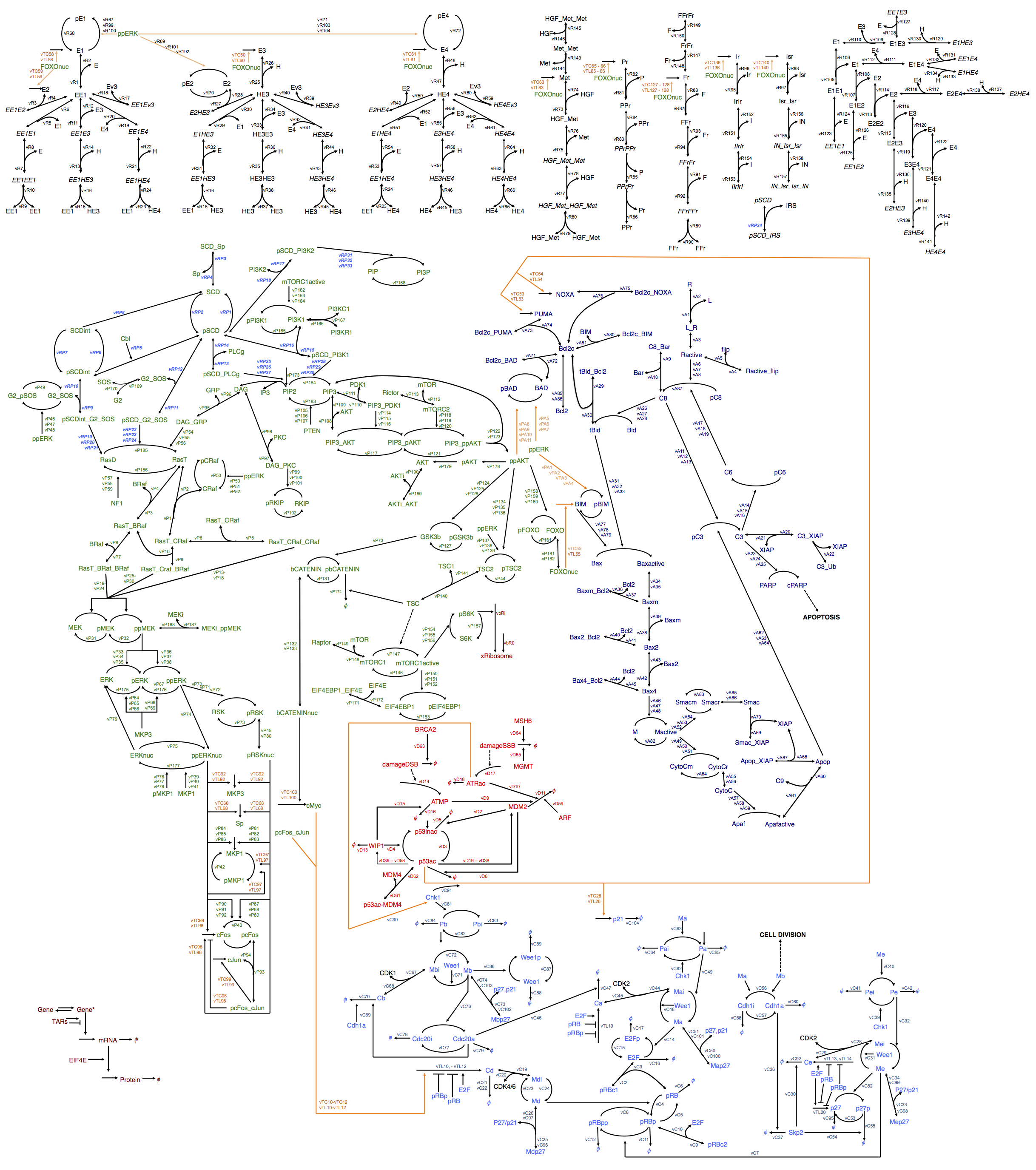

Supplement: S1 Fig — This kinetic scheme depicts all of the reactions in the model. Labels correspond to the indices for each rate law in the equations and code. A “p” prior to a protein name indicates it is phosphorylated. An underscore between two proteins indicates they are in complex. An arrow pointing to a curved arrow indicates three reactions: association, dissociation, and catalysis. A dashed arrow indicates a lack overt mechanism, described with either Hill or Michaelis-Menten rate laws. (TIF) [file pcbi.1005985.s001.tif]

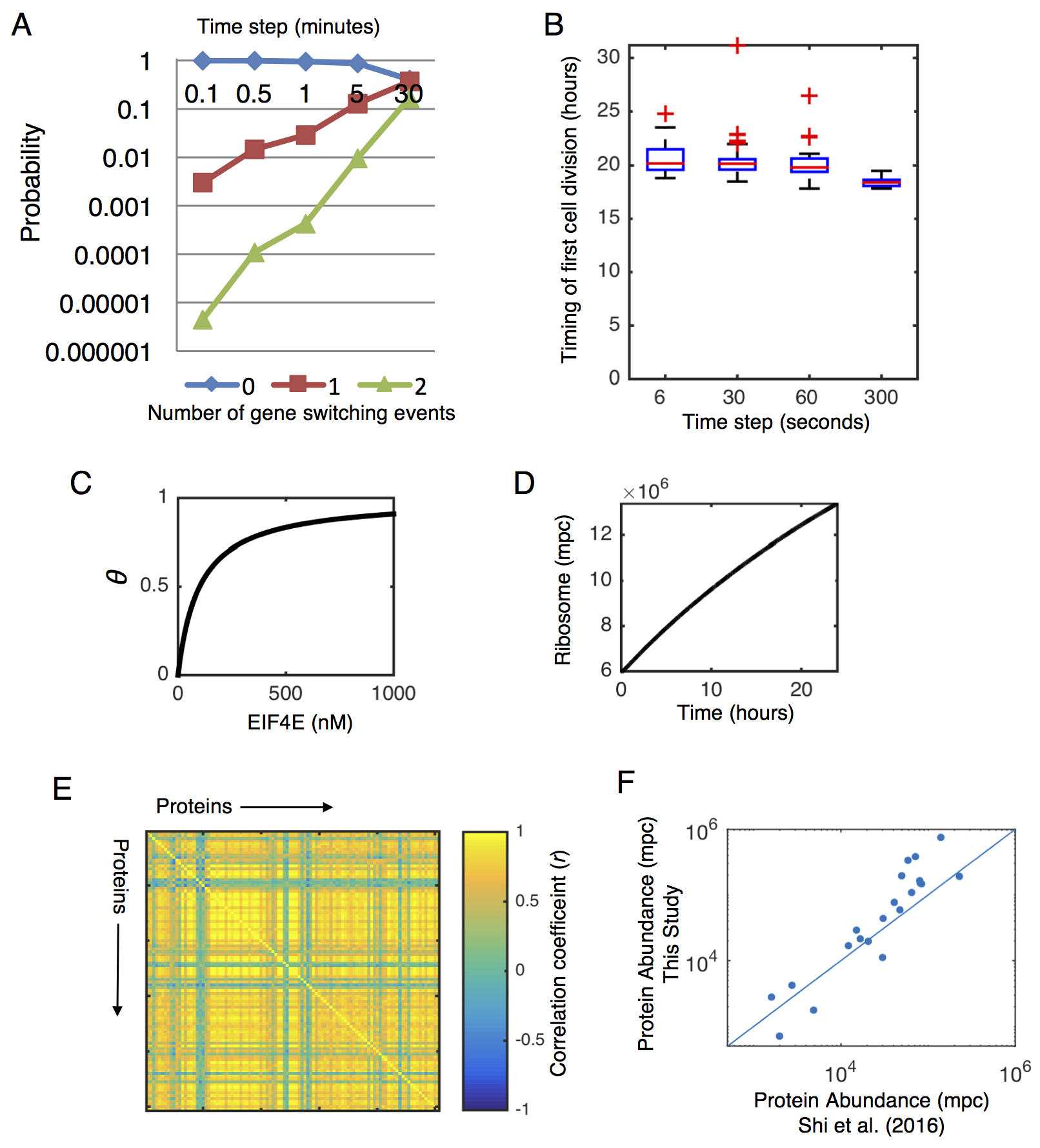

Supplement: S2 Fig — A) Probability of zero (blue diamonds), one (red squares), or two (green triangles) gene switching events given different time step intervals between the stochastic and deterministic components of the model. We chose 30 seconds as a tradeoff. B) Cell-to-cell variability in the timing of the first cell division event following growth factor stimulation. Different time steps between the stochastic and deterministic components of the model are shown along the x-axis. Growth factor stimulation is with all growth factors set to 10nM, except for insulin, which was set to 1721nM. Variability seems rather consistent for the 6, 30, and 60 second time steps, though it collapses when the time step reaches 300 seconds. C) The solution to the hill function (theta) describing the effect of EIF4E on translation rate. D) The number of ribosomes in a single cell doubles during the course of a typical cell cycle duration (~20 hours) in response a full mitogenic stimulus (same as B). E) Correlation between protein levels across a population of cells, showing the typically observed global positive correlations. F) Correlation between our protein quantification and that from Shi et al. [20] in MCF10A cells (genes: ADAM17, ARAF, CBL, DUSP4, EGFR, ERRFI1, GAB1, GRB2, HRAS, KRAS, MAP2K1, MAP2K2, MAPK1, MAPK3, NRAS, PTPN11, PTPRE, RAF1, RASA1, SHC1, SOS1, SOS2). Line is x = y line. (TIF) [file pcbi.1005985.s002.tif]

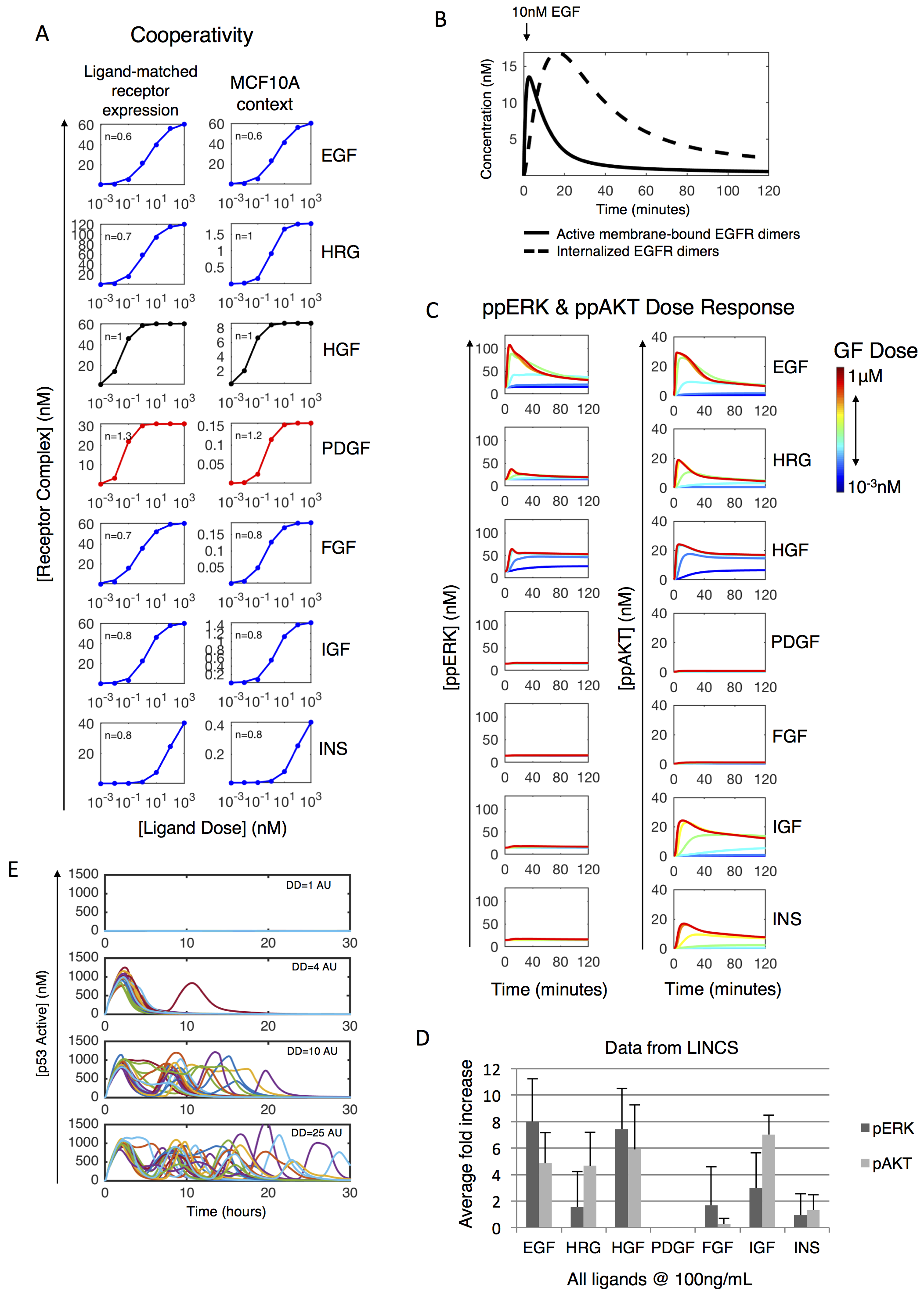

Supplement: S3 Fig — A) Cooperativity profiles comparing the ideal case (left)—when only the receptors that bind each ligand are present on the cell surface and at appreciable levels—to those for the model tailored to expression levels for MCF10A cells (right). B) Time course plot showing dynamics of active plasma membrane-bound EGFR dimers (solid black line) and internalized receptors (dashed black line) in response to 10nM EGF, showing kinetics largely consistent with prior knowledge on trafficking and downregulation. C) Dose response curves for ppERK and ppAKT in the MCF10A-tailored model in response to various ligands at doses ranging from 1μM to 10-3nM. D) Data obtained from the LINCS (Library of Integrated Network Cellular Signatures) project [25] showing average (across 10, 30, and 90 minute time points) levels of phosphorylated ERK (dark grey) and AKT (light grey) in response to ligands (all at 100ng/mL) depicted in model. Relative activation amplitudes are comparable to model predictions from S3C Fig, although IGF induces more ERK activation than predicted by our model. E) Dynamic data from the stochastic simulations that comprise Fig 3F (different cells are different colors). DNA damage increases from top to bottom. Although the number of pulses increases with increasing DNA damage, pulse height and width remain relatively constant. (TIF) [file pcbi.1005985.s003.tif]

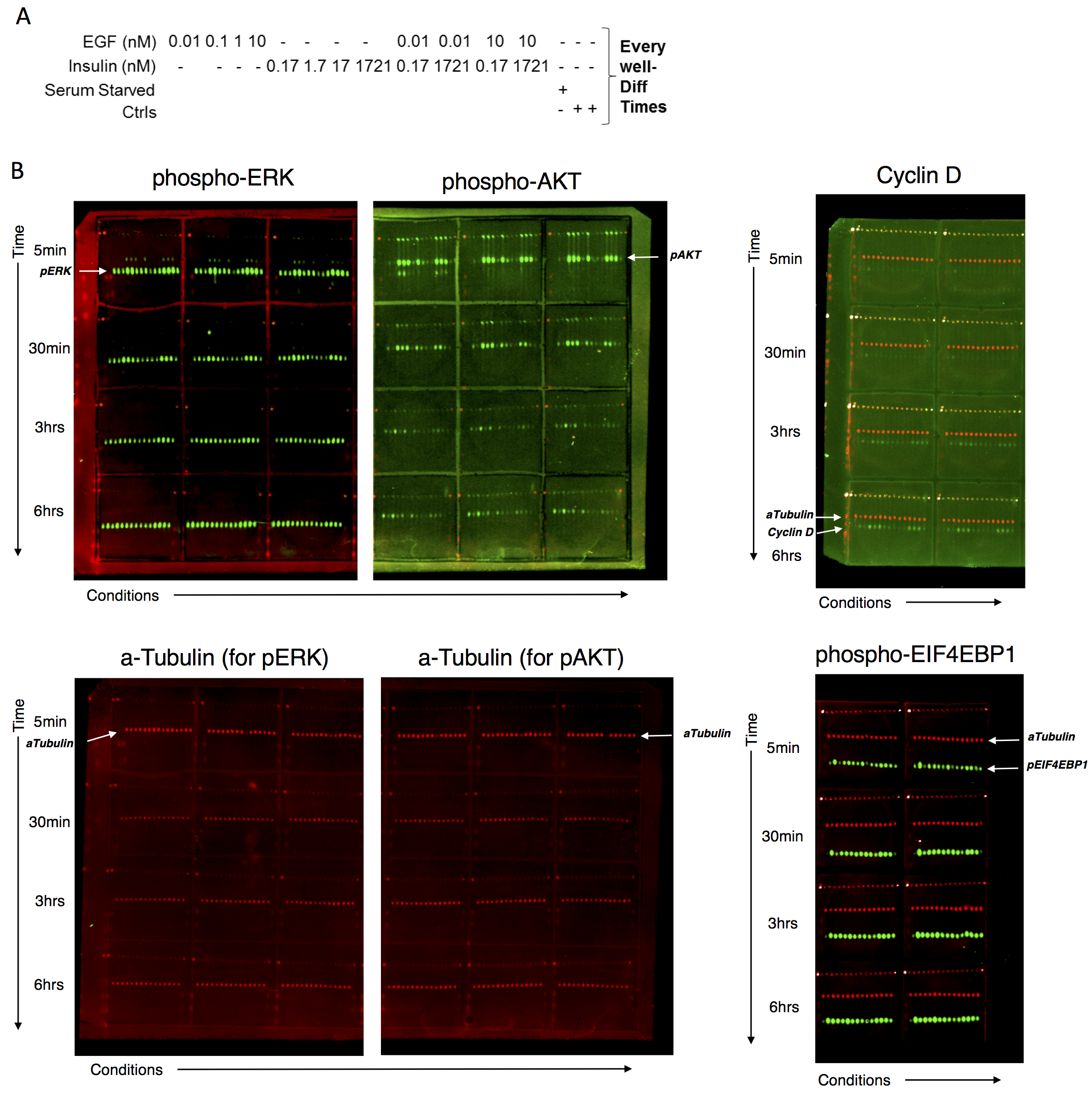

Supplement: S4 Fig — A) Schematic outlining layout of conditions on μ-Western blot for every well. Concentrations are in units of nM. B) Raw scans of μ-Western blot membranes probed for pERK, pAKT, pEIF4E-BP1, α-Tubulin, and cyclin D (see Methods). Time points go from top-to-bottom. Treatment conditions go from left-to-right, as indicated in (A). Each row has biological replicates (three for pERK and pAkt, two for Cyclin D and p4EBP1). (TIF) [file pcbi.1005985.s004.tif]

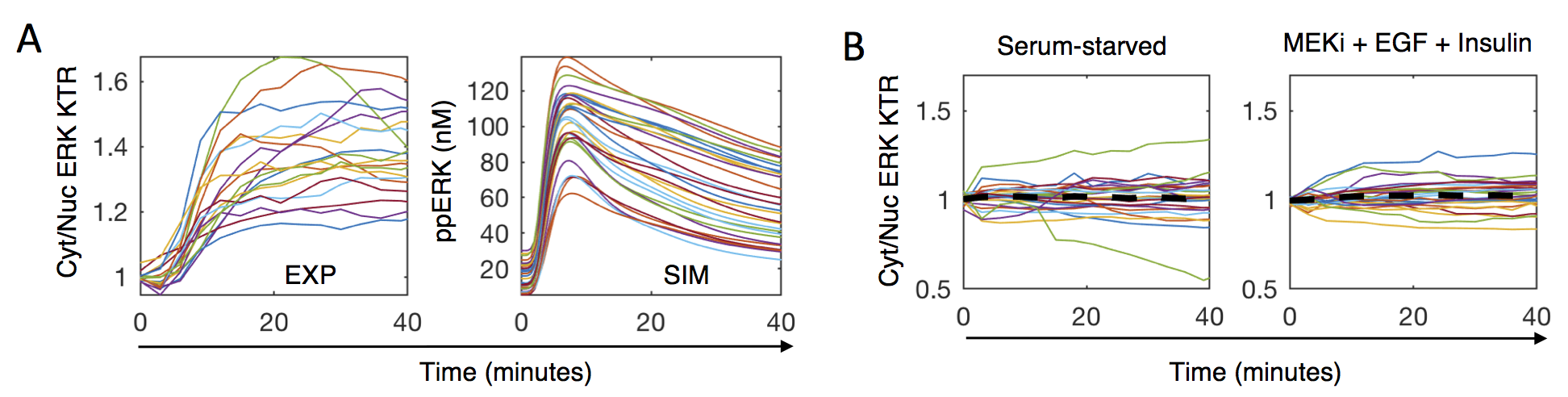

Supplement: S5 Fig — A) Left: Single cell traces of the ratio of cytoplasmic to nuclear fluorescence for ERK KTR probe (reporter of ERK kinase activity) stably expressed by MCF10A cells. Serum-starved cells were stimulated with EGF (20ng/mL) + insulin (10μg/mL). Right: Simulation results showing phosphorylated ERK levels over time stimulated with same as in left. B) Serum-starved and MEK inhibitor (10μM) controls for ERK KTR probe showing no probe activity. (TIF) [file pcbi.1005985.s005.tif]

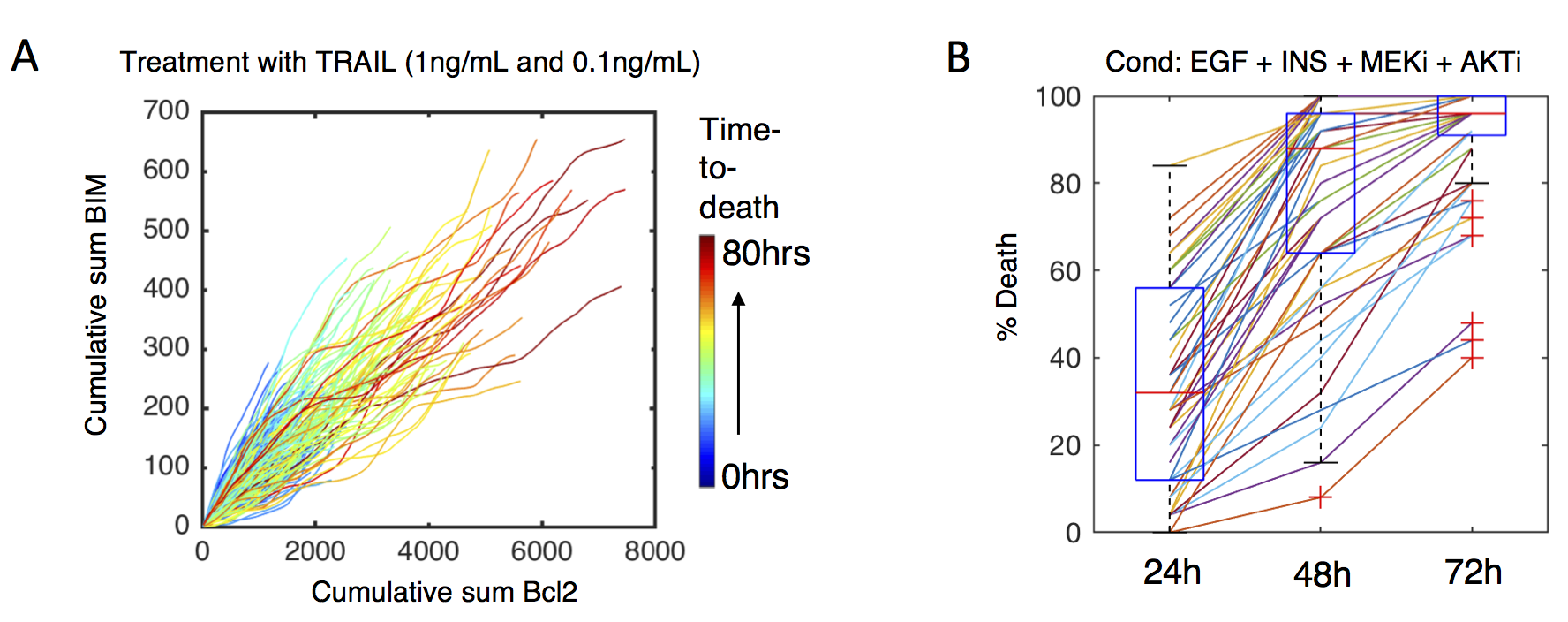

Supplement: S6 Fig — A) Simulated cells are treated with low doses of TRAIL (1ng/mL and 0.1ng/mL) and plots show the cumulative sum of BIM and Bcl2 levels across the time course during which they are alive (analogous to Fig 5F). Cell trajectories are colored in terms of their time to death. Here we see no obvious relationship between BIM and Bcl2 that is dependent on time to death as we saw in Fig 5F, highlighting to the stimulus-specific nature of apoptosis sensitivity. B) Sensitivity of cell death responses (stimulated with EGF + insulin + MEKi + AKTi) to protein half-life was tested by running 53 randomly sampled parameter sets. Degradation rates were altered by a maximum of 2–4 fold from original value (see Methods). Model was re-initialized with each parameter set and the percentage of dead cells at 24, 48, and 72 hours was measured (colored lines indicate different parameter sets). Box and whisker plot quantifies variability between parameter sets (red bar is the median). (TIF) [file pcbi.1005985.s006.tif]

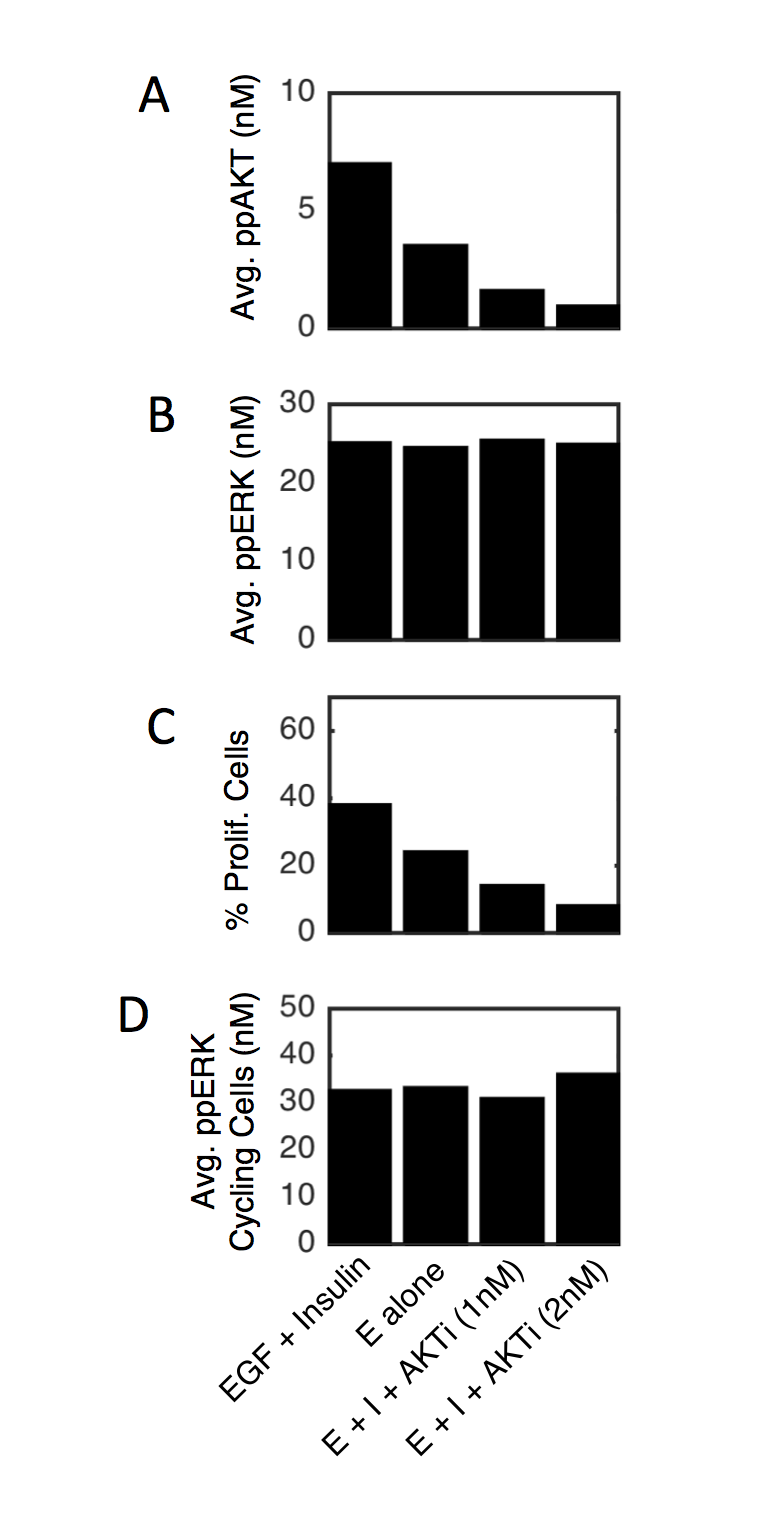

Supplement: S7 Fig — A) The average, time-integrated ppAKT levels across different types of stimuli—EGF (10nM) + insulin (10μg/mL), EGF (10nM) alone, EGF (10nM) + insulin (10μg/mL) + small AKTi dose (1nM), or EGF (10nM) + insulin (10μg/mL) + small AKTi dose (2nM). A decrease in ppAKT is observed. B) The average, time-integrated ppERK levels across the same stimuli as in (A). No decrease in ppERK is observed. C) The percentage of proliferating cells at 24 hours post stimulation reveals that diminishing amounts of ppAKT signal results in a decrease in the percentage of proliferating cells. D) The average, time-integrated ppERK levels for cycling cells reveals no relationship between ppERK levels in cycling cells and ppAKT levels. This indicates that higher AKT activity does not lower the threshold of ERK activity needed to cause cell cycling. (TIF) [file pcbi.1005985.s007.tif]
