# Supplementary material for: A mechanistic pan-cancer pathway model informed by multi-omics data interprets stochastic cell fate responses to drugs and mitogens
Source: PLoS Comput Biol. 2018 Mar 26;14(3):e1005985. doi: 10.1371/journal.pcbi.1005985 (PMC5886578; doi:10.1371/journal.pcbi.1005985)
Supplement: S1 Supplemental Methods — (PDF) [file pcbi.1005985.s008.pdf]

S1 Supplemental Methods

A Mechanistic Pan-Cancer Pathway Model Informed by Multi-Omics Data Interprets Stochastic Cell Fate Responses to Drugs and Mitogens

by

Mehdi Bouhaddou, Anne Marie Barrette, Alan D. Stern, Rick J. Koch, Matthew S. DiStefano, Eric A. Riesel, Luis C. Santos, Annie L. Tan, Alex E. Mertz and Marc R. Birtwistle

Contents

1 Model Overview
1.1 Pathway and submodel selection
1.2 Model structural overview
1.3 Synthesis and degradation
1.4 Hybrid stochastic-deterministic algorithm
1.5 Stoichiometric matrix
1.6 Volumetric compartments
1.7 Ribosomes and cell size

2 Submodel Methods
2.1 Expression
2.2 Receptor Tyrosine Kinase (RTK)
2.3 Proliferation and Growth
2.4 DNA Damage
2.5 Apoptosis
2.6 Cell Cycle
2.7 Submodel integration

3 Initialization procedure
3.1 Initialization rationale, approach, and output
3.2 Step 1: Basal Ras and PIP activities
3.3 Step 2: Basal cyclin D synthesis
3.4 Step 3: Basal caspase 8 cleavage
3.5 Step 4: Basal DNA damage
3.6 Step 5: Transcriptional activators and repressors (TARs)

Chapter 1

Model Overview

1.1 Pathway and submodel selection

We sought to build a model could integrate the major cancer signaling pathways. A pan-cancer analysis by The Cancer Genome Atlas (TCGA) identified four major pathways that are frequently mutated across a variety of human cancers. These are the RTK-Ras-Raf pathway, the PI3K-AKT-mTOR pathway, cell cycle pathways, and p53-DNA repair pathways (Ciriello et al., 2013). To this list we added apoptosis pathways, in order to simulate cell death responses, and gene expression and degradation processes. With these pathways in mind, we created six "submodels": (1) receptor tyrosine kinase (RTK) (Birtwistle, 2015; Birtwistle et al., 2007; Bouhaddou and Birtwistle, 2014; Kiselyov et al., 2009; Park et al., 2003), (2) proliferation and growth (which includes Ras-Raf-MAPK plus PI3K-AKT-mTOR pathways) (Birtwistle et al., 2007; von Kriegsheim et al., 2009; Nakakuki et al., 2010), (3) cell cycle (Gérard and Goldbeter, 2009), (4) apoptosis (Albeck et al., 2008), (5) DNA damage response (Batchelor et al., 2011), and (6) gene expression (the genes/proteins involved in each submodel are noted in main text Figure 1). To create our model, we assembled already-published models from the literature into a whole. The entirely of several submodels were already well represented by published models, such as cell cycle, apoptosis, and DNA damage response, to which we made very minor changes (details in Chapter 2). The RTK, proliferation and growth, and expression submodels were built largely from scratch, though prior models heavily inspired them (see above references). We considered models from the literature based on (*i*) whether they included proteins identified by TCGA as commonly mutated across cancers and (*ii*) whether they were built in conjunction with experimental observations in mammalian cells. All submodels were formatted according to the same rubric: lists of rate laws were extracted from each submodel and a stoichiometric matrix was created to define the elements of each differential equation. As a quality control measure, we ensured that we could reproduce the simulations from each original source model. Once each submodel was finalized, all reactions and stoichiometric matrices were assembled into an integrated system.

1.2 Model structural overview

The model is composed of 1197 total species, which includes all genes, mRNAs, lipids, proteins, and post-translationally modified proteins/protein complexes. There are a total of 141 genes that are considered across all submodels. Each gene possesses an inactive gene product, an active gene product, and an mRNA product (141+141+141=423 total species included in expression submodel). Because many of these genes are functionally redundant, these 141 mRNAs are summed during translation to create 102 "protein conglomerates". Protein conglomerates represent functionally unique proteins (within the scope of our model) (see Supplementary Table 1 for list of genes and their mapping onto protein conglomerates). Once translated, these 102

protein conglomerates can become post-translationally modified in a number of ways; some can be phosphorylated, others form complexes, etc. This creates an additional 672 species across all submodels. These nascent proteins (102) plus their post-translationally modified versions (672) make up a total of 774 protein species (102+672=774). The 423 genes and mRNA species plus the 774 protein species comprise the 1197 total molecular products represented by the model.

We opted to represent the majority of reactions as elementary steps to the extent possible, using mass action kinetics. However, sometimes we employ Michaelis-Menten or Hill representations to capture events where the mechanisms are not sufficiently elucidated. Kinetic parameters are taken from original submodel sources, previous models, experimental studies, or estimated to fit empirical observations, many of which are discussed in the sections that follow.

1.3 Synthesis and degradation

The expression submodel controls the synthesis of most proteins in the model. The production of mRNA is based on a stochastic algorithm we devised (detailed in Chapter 2). Protein synthesis, v_{bp_j}, for each protein, j, is defined by (see Chapter 2 for derivation):

$$v_{bp_j} = k_{TL_j} \cdot m_{Total_j} \cdot \frac{[EIF4E]}{k_D + [EIF4E]} \qquad \text{(E1)}$$

This equation is used to describe the synthesis of the majority of proteins in the model, with a few exceptions in the cell cycle and DNA damage submodels (details in Chapter 2).

Protein synthesis in cell cycle and DNA damage submodels. The original cell cycle (Gérard and Goldbeter, 2009) and DNA damage (Batchelor et al., 2011) models that we acquired already possessed their own synthesis reactions. Due to the dynamical complexity of these submodels, we needed a way to reconcile their original synthesis reactions with our equations. To do this, we required that, at steady state:

$$v_{bp_j} = \sum_{i=1}^{n} v_{syn_i} \qquad \text{(E2)}$$

Where v_{syn_i} equals each original synthesis reaction, i, for a particular protein, j. For example, the synthesis of MDM2 is defined by a zero-order reaction plus a reaction that is dependent on the levels of active p53 ($\sum v_{syn} = bmi + bm \cdot p53^*$). To meet this requirement, we defined k_{TL_j}, a translation rate constant for a particular protein, j, as a dynamic term that could account for changes in v_{syn}. We thus define k_{TL_j} as:

$$k_{TL_j} = \frac{\sum_{i=1}^{n} v_{syn_i}}{\left(m_{SS_j} \cdot \frac{[EIF4E_{SS}]}{k_D + [EIF4E_{SS}]}\right)} \qquad \text{(E3)}$$

Here, m_{SS_j} represents the levels of mRNA at steady state (from RNAseq data) for a given gene, j and $EIF4E_{SS}$ are the levels of free EIF4E at steady state (defined during initialization procedure, described in Chapter 3). The final equation for protein production for species in the cell cycle and DNA damage submodels is:

$$v_{bp_j} = \frac{\sum_{i=1}^{n} v_{syn_i}}{\left(m_{SS_j} \cdot \frac{[EIF4E_{SS}]}{k_D + [EIF4E_{SS}]}\right)} \cdot m_{Total_j} \cdot \frac{[EIF4E]}{k_D + [EIF4E]} \quad \text{(E4)}$$

Where m_{Total_j} and $EIF4E$ are levels of total mRNA for every gene, j, and free EIF4E currently in the system. By doing this, we allow for natural fluctuations in m_{Total_j} and $EIF4E$ levels to affect the overall translation rate of proteins in the cell cycle and DNA damage submodel. However, as you will see (below and in Chapter 2.6) we did not allow the majority of cell cycle proteins to be stochastically regulated (with the exception of cyclin D and p21), as it resulted in non-biologically plausible behavior.

Lipid synthesis and degradation. Lipids represented in the model include phosphatidylinositol (3,4,5)-triphosphate (PI(3,4,5)P or PIP_3) phosphatidylinositol 4,5-bisphosphate (PIP_2), diacylglycerol (DAG), and inositol triphosphate (IP_3). In our model, PIP_2 is synthesized by a zero-order reaction. PIP_2 can be cleaved by PLCγ to produce DAG and IP_3. PIP_2 can also be phosphorylated by PI3K to become PIP_3. All lipids are degraded by first-order degradation reactions. Degradation rate constants for all lipids are taken from a literature source and references therein (Zhang et al., 2014). We increased the degradation of DAG by a factor of 10 to fit ERK signaling dynamics to experimental data.

Protein degradation. Proteins (protein conglomerates and post-translationally modified proteins) and transcripts follow first-order degradation kinetics. Protein and mRNA half-lives were taken from three sources, with preference given to the first source, then if a gene was not found to the second source, and so on: i) (Schwanhausser et al., 2011), ii) (Tani et al., 2012) (only for mRNA), iii) from miscellaneous literature references on mammalian cells (see Supplementary Table 1 for gene-specific half-life values and sources). Half-lives (τ) are converted into first-order degradation rate constants (k_{deg}) using the following equation:

$$k_{deg} = \frac{\ln(2)}{\tau} \quad \text{(E5)}$$

An additional layer of complexity arises when we consider the degradation of post-translationally modified protein monomers or protein complexes. In the absence of evidence for post-translational regulation of protein stability, degradation rates of post-translationally modified protein monomers were set equal to that of the non-post-translationally modified monomer. However, there are instances when, for example, the phosphorylated form of a protein may be degraded at a higher rate, due to the recruitment of degradation machinery, or, conversely, may be stabilized and thus degraded more slowly. For example, altered degradation rates have been shown to occur for cleaved caspases (Tawa et al., 2004), phosphorylated β-Catenin (Aberle et al., 1997), phosphorylated BIM (Luciano et al., 2003), phosphorylated BAD (Howie et al., 2008), phosphorylated FOXO (Fu et al., 2009; Matsuzaki et al., 2003; Smith and Shanley, 2010), and phosphorylated cFos and DUSP proteins (Nakakuki et al., 2010a and references therein), which we encoded in the model. Regarding the degradation of protein complexes, we assumed that the degradation rate of each complex is equal to the degradation rate

of the most quickly degraded protein in the complex. Sometimes, however, the binding between two proteins can serve as a stabilizing force. We employ this reasoning to the degradation of phosphorylated cFos bound to cJun (AP1 complex), which we set equal to the literature-acquired degradation rate for phosphorylated cFos. We do this because phosphorylated cFos has been shown to be more stable compared to the relatively quick degradation rate for unphosphorylated cFos (Murphy et al., 2002; Nakakuki et al., 2010). Enabling AP1 to have an identical degradation rate also prolongs the AP1 signal in response to a mitogenic stimulus.

The cell cycle and DNA damage submodels possessed built-in degradation reactions, many of which had non-linear components. To retain the fidelity to the original model, we left degradation reactions in these submodels as they were. Exceptions to this rule include proteins we added to these models *de novo* or proteins that connect submodels; these are cyclin D, p21, BRCA2, MGMT, MSH6, and MDM4. The degradation reactions for these exceptions are modeled using the same first-order degradation reactions as in the other submodels.

Failure of incorporating stochastic gene expression into cell cycle submodel. During a stochastic simulation, all genes are simulated stochastically except for the majority of cell cycle genes. Cyclin D and p21 are the only two cell cycle genes that are simulated stochastically. Interestingly, incorporating stochastic gene expression processes into the entire cell cycle submodel created non-biologically plausible behavior. Principally, cells would randomly commit to cell division in the absence of a mitogenic stimulus. We attempted to alleviate this artifact through multiple modeling additions including increased availability and number of cyclin dependent kinase inhibitors, but could not find the solution. Therefore, to prevent this behavior, we only permitted stochastic control of cyclin D and p21 in our model; the other cell cycle proteins were simulated deterministically. Of course, stochastic variation in cyclin D and p21 levels propagate through to other cell cycle proteins, which introduces a much smaller degree of noise into the submodel compared to when each gene is simulated stochastically. We also did not allow any variations in the levels of EIF4E to affect cell cycle proteins other than cyclin D and p21. This result suggests that there may be hitherto unknown mechanisms by which the cell prevents against random cycling other than those formalized in this model.

1.4 Hybrid stochastic-deterministic algorithm

We desired to integrate stochastic gene expression processes into our model in order to capture natural fluctuations in protein levels that may render a cell more or less sensitive to a particular stimulus. We designed a hybrid stochastic-deterministic modeling framework in order to increase computational efficiency above the low efficiency expected of a fully stochastic algorithmic approach. Although the number of gene copies in a cell (roughly 2 copies) and the number of mRNAs in a cell (~20 copies per cell on average) are relatively low, and thus amenable to efficient stochastic simulation, the number of protein molecules can approach hundreds of thousands of molecules per cell, rendering stochastic simulation of them inefficient. Conveniently, however, as molecule number approaches that typical of protein levels, the effects of stochasticity become small to negligible. Thus, we decided to simulate gene switching and mRNA birth-death stochastically and all protein processes (e.g. phosphorylation, dimerization, protein degradation, etc.) deterministically. Updated mRNA quantities were passed from the stochastic to the deterministic component. Signaling entities that act as transcriptional activators

or repressors were transferred in the reverse direction, from the deterministic to the stochastic component. Stochastic implementation was based on Poisson processes (details in Chapter 2.1).

A critical concern was defining a time step between the stochastic and deterministic components that balanced computational efficiency with the need to capture the maximum number of stochastic gene switching events. We selected a time step of 30 seconds, which was the maximum time step ensuring (*i*) the probability of two gene switching events occurring within a single time step was low (below 0.1%; Figure S2A) and (*ii*) phenotypic behavior was not altered, such as timing of cell cycle entry (Figure S2B). This latter point was evaluated during model development.

1.5 Stoichiometric matrix formulation

In order to simplify and organize model implementation, we use the stoichiometric matrix formalism (Jeong et al., 2000; Schilling and Palsson, 1998). We created a matrix that describes the stoichiometry (either a 0, 1, -1, 2, or -2) between species (rows) and reactions (columns) in the model. The left hand side of the system of ODEs at each time step is calculated using the following matrix equation:

$$\dot{\mathbf{y}} = \mathbf{S} \cdot \mathbf{v} \qquad \text{(E6)}$$

If n is the number of species and m is the number of reactions, then **S** is an n-by-m stoichiometric matrix, $\mathbf{v}$ is an m-by-1 vector of rate laws, and $\dot{\mathbf{y}}$ is an n-by-1 vector of the first time derivatives of model species concentrations. We modified this basic form to correct for cell compartment volumes (see Section 1.6, below, about Compartments).

1.6 Compartments

The model contains four compartments with associated volumes, V: extracellular space (V_e), cytoplasm (V_c), nucleus (V_n), and mitochondria (V_m). A typical mammalian cell has a volume between 100-10,000μm^3, or 0.1-10pL (Milo et al., 2009). As a middle ground and based on visual inspection of phase contrast images, we approximated our total cell volume (V_T) as 7pL (Ballesta et al., 2014; Geltmeier et al., 2015). Based on dimensions from phase contrast images, we determined the nucleus to be roughly a quarter of the cell's volume (thus, $V_n = V_T/4$). We then calculated the size of the cytoplasm to be equal to the difference between the total cell volume and nuclear volume ($V_c = V_T - V_n$). The mitochondrial volume was previously estimated to be approximately 7% of the size of the cytoplasm (Albeck et al., 2008). For cell culture experiments, the volume of the extracellular space is much larger than the volume of single cells. We set the volume of the extracellular space to be 50μL, which is the volume of media typically placed into one well of a 96-well plate. Although experiments are sometimes done in different vessel sizes, this large volume ratio renders simulations insensitive to typical variation across experimental culture conditions.

Based on their cellular localization, every species and every reaction was assigned one of these four home compartments. Importantly, when modeling in terms of concentration, one must introduce volume corrections when (*i*) reactants in a rate law possess different home compartments or (*ii*) rate laws comprising a differential equation possess different home compartments. When a reaction possesses reactants with different home compartments, we

introduce a volume correction term to volume correct the outsider reactant to be defined in terms of the home compartment of the reaction, as such:

$$v_B = k \cdot X_A \cdot Y_B \cdot \left(\frac{V_A}{V_B}\right) \quad \text{(E7)}$$

Where X_A is a species in a compartment of volume V_A, X_B is a species in a compartment of volume V_B, and v_B is a reaction with a home compartment of volume V_B. For example, if we consider the reaction describing the binding of EGF to EGFR, which takes place in the extracellular space, the home compartment of EGF is the extracellular space (V_e) whereas the home compartment of EGFR is the cytoplasm (V_c). In this case, we correct the concentration of EGFR to be in terms of the extracellular volume by multiplying the first order reaction by V_c/V_e.

In addition to this bimolecular volume correction on the reaction level, sometimes an ordinary differential equation can possess reaction terms that are of different home compartments. To correct for this, we multiply every reaction by its respective home compartment to remove units of volume. We multiply this vector of "volume-less" reaction values, $\mathbf{v_{VC}}$, by our stoichiometric matrix, producing an n-by-1 vector $\dot{\mathbf{n}}$ with units of nmol / time (and length equal to the total number of species, *n*):

$$\dot{\mathbf{n}} = \mathbf{S} \cdot \mathbf{v_{VC}} \quad \text{(E8)}$$

Finally, to add units of volume back to our $\dot{\mathbf{n}}$ quantities, we element-wise divide by the home compartment of each species, which produces our final n-by-1 vector of the first time derivatives of model species concentrations, $\dot{\mathbf{y}}$.

1.7 Ribosomes and cell size

A cell must approximately double its contents prior to cell division; else, repeated rounds of cell division would eventually push a cell population to non-existence. One predominant mechanism for this is an increase in the number of ribosomes, which increases the translation rate globally across all proteins. We thus use ribosome quantity (related to ribosome number and not concentration) as a proxy for cell size.

$$V = \alpha \cdot R$$

Here, V is total cell volume, R is the number of ribosomes (molecules per cell), and α is a proportionality constant that converts ribosome number linearly to cell size (based on an initial ribosome number of 6 million molecules per cell and an initial cell size of 7 picoliters, α=1.17x10^{-18} liters/molecules). This has the effect of keeping ribosome concentration constant, which we lump into the effective translation rate constant. Therefore, we are not able yet to account for how stochastic fluctuations in ribosome levels affect protein expression noise. However, since the average number of ribosomes in a mammalian cell, at ~6,000,000 molecules per cell (Milo et al., 2009), is much greater than the average number of mRNA molecules, at ~400,000 molecules per cell (Alberts, B. Johnson, A. Lewis, J. Raff, M. Roberts, K. Walter, 2008), we expect such fluctuations to be small compared to stochastic gene switching and mRNA birth-death, which we account for.

The de novo synthesis of ribosomes for cell growth and proliferation has been linked to activation of p70 S6K (Chauvin et al., 2014). This, on top of synthesis (zero-order) and turnover (first-order) terms for ribosome homeostasis, gives rise to the following differential equation for ribosome dynamics:

$$\frac{d[R]}{dt} = kb_0 + kb_i \cdot \left(\frac{[pS6K]^n}{{K_{50}}^n + [pS6K]^n}\right) - kd_0 \cdot [R] \quad \text{(E9)}$$

We determined the degradation rate for ribosomes by taking the average of the half-lives reported by Schwanhausser et al (2011) for all 40S and 60S ribosomal proteins.

To calculate kb_i, the rate constant governing the upregulation of ribosomes by $pS6K$, we assumed that $pS6K$ levels in the serum-starved state are low (approximately ~1% of the total pool of S6K). In a highly mitogenic environment, we assume S6K will be highly phosphorylated (~50%). We then estimated the value of kb_i (value of 0.04 $nM^{-1}s^{-1}$) such that, in response to a mitogenic stimulus, ribosome numbers would double during the course of one cell cycle (Figure S2D), which for MCF10A cells was determined to be between 16 and 24 hours (Albeck et al., 2013).

Chapter 2

Submodel Methods

Here we describe more details about each submodel. Therein, we detail any changes we made to the original source models, where applicable. We also discuss the major biological behaviors we desired that each submodel possess. These are outlined as follows:

Submodel	Required Properties
Expression	• Model is tailored to genomic, transcriptomic, and proteomic context of MCF10A cells.<br>• Stochastic gene expression is simulated with a computationally efficient algorithm.<br>• Cell-to-cell variability in mRNA and protein levels matches experimental observations.<br>• EIF4E levels possess extrinsic control over the translation rate.<br>• Ribosomes double during the course of one cell cycle.
Receptor Tyrosine Kinase	• Ligand-receptor cooperativity matches experimental observations<br>• Receptor trafficking kinetics reflects experimental observations.
Proliferation & Growth	• Receptors possess unique pathway preferences that match experimental observations.<br>• Basal, serum-starved state activity flux through ERK and AKT pathways exists.<br>• Dynamic dose responses of ERK, AKT, and mTOR signaling matches experimental data.
DNA Damage	• Original delayed differential equations are converted into ordinary differential equations.<br>• p53 dynamics corresponding to single- and double-stranded DNA breaks matches experimental observations.<br>• Rate of DNA damage repair is dependent on levels of repair enzymes.<br>• p53 activation dynamics exhibit “digital” and not “analog” behavior, whereby the number of p53 pulses, but not pulse height or width, scales to magnitude of DNA damage.<br>• Etoposide-induced DNA damage is dependent on the cell cycle stage (S-phase).
Apoptosis	• Robustness against small death signals.<br>• Model exhibits all-or-nothing death response when apoptosis signaling surpasses threshold.<br>• Dose and dynamics of TRAIL-induced extrinsic apoptosis matches experimental observations.

	• Intrinsic apoptosis signaling responds to interrupted survival signaling and DNA damage induced upregulation of pro-apoptotic proteins.
Cell Cycle	• Cell cycle entry is driven by induction of cyclin D mRNA.<br>• Order and timing of cyclin/cdk complexes matches established observations.<br>• Cell cycle duration matches duration measured in MCF10A cells.<br>• Upregulation of p21 (a cyclin dependent kinase inhibitor) arrests the cell cycle.

2.1 Expression

About. The expression submodel describes genes switching between an inactive and active state, transcription of the active gene to create mRNA, and translation of the mRNA to create protein. The mRNA and protein products can be degraded. Some genes in the model possess explicitly coded transcriptional activators and/or repressors, which can affect the transcription rate (see equations below). The translation rate of an mRNA is dependent on the mRNA concentration in the cell as well as on the levels of EIF4E, a rate-limiting cap-dependent translation initiation factor. Importantly, each mRNA and protein possesses a unique synthesis and degradation rate, resulting in dynamical behaviors that may play important roles in constraining protein function and phenotypic outcomes. Moreover, these gene-specific attributes are known to vary significantly across the genome (Schwanhausser et al., 2011). Therefore we sought to capture these gene-specific attributes to the extent possible.

Transcription and translation rates are estimated as part of the initialization procedure (see Chapter 3). mRNA and protein degradation rates are calculated from half-life measurements found in the literature (Supplementary Table 1). Although these half-lives were procured from different mammalian cell lines and cell types, we assume mRNA and protein half-lives to be fairly consistent between cell types. This is inspired by (*i*) the fact that gene-specific ratios of mRNA-to-protein are fairly consistent between different human cell types (Wilhelm et al., 2014) and (*ii*) evidence from several studies that report conservation of mRNA and protein half-lives across species (Friedel et al., 2009; Schwanhausser et al., 2011). These points give us confidence that mRNA and protein half-lives taken from other mammalian cell contexts serve as reasonable approximations of half-life estimates for MCF10A cells, and that altering protein levels through translational means, as we implement, is preferred. Finally, this submodel possesses a stochastic component; specifically, in gene switching and mRNA births and deaths. Translation and protein degradation are simulated deterministically, though fluctuations from gene expression propagate into protein levels over time. As described above, the model switches from the stochastic to the deterministic component (and back again) every 30 seconds of simulation time.

Epigenetics. Rates of gene switching were acquired from an experimental study (Suter et al., 2011). The study established a transgenic cell line expressing a short-lived luciferase from an unstable mRNA, allowing them to measure gene activation and inactivation kinetics for a handful of genes. The paper defined an average gene activation and inactivation rate, which we used for each gene in our model, similar to other modeling work (Bertaux et al., 2014) (see Table SI1).

Table SI1. Rate constants for gene activation and inactivation.

k_{gac} (gene activation)	0.03 min^{-1}
k_{gin} (gene inactivation)	0.3 min^{-1}

We model gene switching as a Poisson process. The Poisson distribution is a discrete probability distribution that describes the probability that a given number of events will occur within a certain time interval, independently from when the last event occurred. It is parameterized by lambda, which is defined as the expected number of events in the considered time interval. For gene switching, lambda is defined as the rate of gene activation, or inactivation, multiplied by the time interval:

$$\lambda_{gac} = k_{gac} \cdot t \quad \text{(E10)}$$

$$\lambda_{gin} = k_{gin} \cdot t \quad \text{(E11)}$$

For each gene, we calculate the probability of getting zero switching events using the `poisspdf` function in Matlab evaluated at zero. This defines two probabilities: p_{off} (probability that a gene that was on would stay on; calculated using λ_{gin}) and p_{on} (probability that a gene that was off would stay off; calculated using λ_{gac}). Next, a random number was generated for each gene between 0 and 1 using the `rand` function. For genes that were on, if their random number was greater than or equal to p_{off}, then they were switched from on to off. Similarly, for genes that were off, if their random number was greater than or equal to p_{on}, then they were switched from off to on.

Transcription. Transcription and mRNA degradation were also modeled as Poisson processes. The number of mRNA births and deaths per time step were randomly sampled from Poisson distributions using the `poissrnd` function in MATLAB. Again, lambda terms were defined as the transcription rate or degradation rate of a gene multiplied by the time interval. The rate of transcription was determined by summing a leak term, which accounts for all non-modeled or constitutive transcription, and an induction term, which accounts for all modeled transcriptional induction. We define the rate law for transcription as:

$$v_{bm} = \overbrace{k_{leak} \cdot g^*}^{\textit{Leak term}} + \overbrace{k_{max} \cdot \left(\frac{\sum \left(\frac{[TA_i]}{KA_{50i}} \right)^{nA_i}}{1 + \sum \left(\frac{[TA_i]}{KA_{50i}} \right)^{nA_i} + \sum \left(\frac{[TR_i]}{KR_{50i}} \right)^{nR_i}} \right) \cdot g^*}^{\textit{Induction term}} \quad \text{(E12)}$$

Here, k_{max} is the maximal transcription rate of RNA polymerase (same for all genes), which we determined to be 0.1 molecules per second (Iyer and Struhl, 1996; Kugel and Goodrich, 2000). For every transcriptional activator (TA_i) or repressor (TR_i), i, KA_{50i} and KR_{50i} are the concentrations needed to achieve their half-maximal effect on the transcriptional output, respectively. nA_i and nR_i values are hill coefficients for activators and repressors, respectively. K_{50} and hill coefficient values were tuned as part of model training. Hill functions such as these are commonly used to describe transcriptional induction terms (Alon, 2007; Nakakuki et al., 2010; Thattai and van Oudenaarden, 2001). The number of active or inactive genes in the cell are denoted by g^* or g, respectively.

The rate law for mRNA degradation is a first order term:

$$v_{dm} = k_{dm} \cdot m \tag{E13}$$

Here, k_{dm} is a rate constant derived from mRNA half-life data taken from the literature for each mRNA (Supplementary Table 1), and m is the number of mRNA molecules in the cell. Thus, the lambda terms for the Poisson distributions defining transcription and mRNA degradation are:

$$\lambda_{bm} = v_{bm} \cdot t \tag{E14}$$

$$\lambda_{dm} = v_{dm} \cdot t \tag{E15}$$

Translation. Equations describing translation are simulated deterministically. We derived a differential equation for translation that accounted for extrinsic control based on the levels of EIF4E in the cell, a critical initiation factor that binds to the mRNA 5' cap to recruit it to an available ribosome (Mamane et al., 2004). Because translation initiation is thought to be the rate-limiting factor in protein production (Kudla et al., 2009; Sonenberg and Hinnebusch, 2009), we take the rate of translation initiation, and thus the rate of translation, to be linearly proportional to the amount of mRNA bound to an EIF4E molecule (m^*):

$$v_{bp} = k_{TL} \cdot m^* \tag{E16}$$

Here, k_{TL} implicitly includes the dependence on ribosome concentration, which is constant in our model because cell volume is proportional to ribosome number.

Next we sought to solve m^* in terms of the total amount of mRNA per cell, a metric that is experimentally quantifiable from the output of mRNA sequencing experiments. The differential equation describing the binding between free mRNA (m_{free}) and EIF4E is defined as:

$$\frac{dm^*}{dt} = k_{T1} \cdot [EIF4E] \cdot m_{free} - k_{T2} \cdot m^* \tag{E17}$$

Solving for m^* at steady state yields:

$$m^* = \frac{k_{T1} \cdot [EIF4E] \cdot m_{free}}{k_{T2}} \tag{E18}$$

Given mRNA moiety conservation ($m_{Total} = m_{free} + m^*$), and the definition of the dissociation constant K_D, yields

$$m^* = \frac{m_{Total} \cdot [EIF4E]}{K_D + [EIF4E]} \tag{E19}$$

Plugging this definition for m^* back into Equation E16 gives our final equation for the rate of translation:

$$v_{bp} = k_{TL} \cdot m_{Total} \cdot \frac{[EIF4E]}{K_D + [EIF4E]} \tag{E20}$$

The final differential equation for proteins is also noted above in Equation E1.

2.2 Receptor Tyrosine Kinase (RTK)

About. The receptor tyrosine kinase submodel contains many receptors that are frequently overexpressed or mutated in cancer; these include the ErbB family (Her1-4), the hepatocyte growth factor receptor (cMet), the platelet-derived growth factor receptor (PDGFR), the fibroblast growth factor receptor (FGFR), the insulin-like growth factor receptor (IGFR), and the insulin receptor (INSR). For all receptors except for PDGFR, we allow receptors to form dimers in the absence of ligand, though at very low affinities. IGFR and INSR are exceptions, forming covalent cysteine bonds between receptor dimers prior to ligand binding and activation (Kiselyov et al., 2009).

Ligand stimulation induces receptor dimerization. Ligand can bind to either a receptor monomer or a preformed receptor dimer. Once a ligand binds a receptor monomer, it can dimerize with another receptor monomer or another ligand-bound receptor (forming either LRR or LRLR type species). The ligand binding to the preformed dimer can subsequently bind another ligand. Once receptor dimers are in complex with either one or two ligands, we term them signaling competent dimers (SCDs). For the ErbB family of receptors, EGF can only bind to EGFR, ErbB2 has no known ligand, and ErbB3 and ErbB4 can bind to heregulin. All family members can dimerize with one another, creating a plethora of possible combinations.

The formation of these SCDs induces the activation of the tyrosine kinase domain and the phosphorylation of intracellular tyrosine residues. This can recruit adaptor proteins, which mediates downstream signaling. For IGFR and INSR receptors, we require an additional step—binding to IRS—prior to phosphorylation. To construct the schematic for PDGFR, we used a simplified scheme presented by Haugh and colleagues (Park et al., 2003). In this scheme, the binding of ligand to PDGFR induces it to bind to another ligand-bound receptor. From this complex, one ligand can dissociate, which provokes the subsequent release of one receptor molecule. For a detailed model schematic please refer to Figure S1. All rate constant values, and sources for each rate constant, for the receptor tyrosine kinase submodel (as well as for the proliferation and growth submodel) can be found in Supplementary Table 2.

Ligand-receptor cooperativity. In order to reproduce appropriate dose-response behavior for each ligand-receptor system we ensured that the cooperativity behavior for each ligand-receptor system matched experimental observations from the literature (see table below). Specifically, we ensured the appropriate fold change between the K_D of the 1st and 2nd ligand-binding sites (see Table SI2). For example, it is thought that the binding of the first EGF molecule to EGFR occurs with ~10 fold higher affinity than the binding of the second ligand (Alvarado et al., 2010). Because receptor cooperativity measurements can vary between cell types, we sought only that cooperativity behavior was in the correct category – either displaying negative, positive, or no cooperativity (Figure 3A and S3A in main text). As a general rule, we attempted to keep ligand-receptor association rate constants consistent across reactions and slower than diffusion encounter rate (we take them as 0.001 $nM^{-1}s^{-1}$). Ligand dissociation rate constants were tailored to match experimental observations as described. Also, wherever possible, we used generic receptor dimerization (0.01 $nM^{-1}s^{-1}$) and dissociation (0.001 s^{-1}) rate constants, which serve as reasonable approximations for these reactions for which direct experimental measurement is difficult (Birtwistle, 2015). It was not always possible to match cooperativity and dynamics while satisfying detailed balance; this may be due to unmodeled but implicit energy dependent steps such as cycles of phosphorylation coupled to dimerization, or other receptor microdomain compartmentalization that is also energy dependent.

Table SI2. Receptor cooperativity parameters and literature sources.

Ligand	Receptor(s)	Fold difference between binding sites	Cooperativity	Literature Sources
EGF	EGFR	10	Negative	(Alvarado et al., 2010)
HRG	ErbB3, ErbB4	10	Negative	(Hiroshima et al., 2012)
HGF	c-Met	1	None	(Takahashi et al., 1996)
PDGF	PDGFRα, PDGFRβ	NA *	Positive	(Park et al., 2003)
FGF	FGFR1, FGFR2	500	Negative	(Mohammadi et al., 2005; Story et al., 1994)
IGF	IGF1R	100	Negative	(Christoffersen et al., 1994; Kiselyov et al., 2009; De Meyts and Whittaker, 2002)
INS	INSR	100	Negative	(Christoffersen et al., 1994; Kiselyov et al., 2009)

* PDGFR was coded according to a simplified scheme depicted in a literature source (Park et al., 2003).

Receptor trafficking dynamics. Once receptors bind their cognate ligands and form dimers, they phosphorylate one another. Phosphorylated signaling competent receptor dimers can also be internalized and targeted for degradation, or can be recycled back to the plasma membrane from early endosomes. Receptor internalization and degradation is an important mechanism by which pathways shut off after acute stimulation (in addition to any negative feedback that might exist). Importantly, internalization and degradation kinetics can vary between receptor dimer types (see Table SI3). Of note, dimers containing EGFR are known to internalize and degrade 10X faster, and recycle back to the plasma membrane 10X slower, than when an EGFR molecule is not

involved (Resat et al., 2003). Rate constants for internalization, degradation, and recycling were set to the following:

Table SI3. Receptor internalization and degradation kinetics.

Dimer type	Internalization of pSCDs (s^{-1})	Degradation rate of internalized SCDs (s^{-1})	Recycling rate of internalized SCDs (s^{-1})	Sources
EGFR+EGFR	4.67×10^{-3}	8.37×10^{-4}	8.37×10^{-4}	(Shankaran et al., 2013)
EGFR+other	1.67×10^{-3}	2.13×10^{-4}	8.52×10^{-4}	(Shankaran et al., 2013)
other+other	1.67×10^{-4}	8.71×10^{-5}	1.4×10^{-3}	(Shankaran et al., 2013)
IGFR	7.93×10^{-5}	8.71×10^{-5}	7.7×10^{-7}	(Kiselyov et al., 2009)
INSR	1.67×10^{-4}	8.71×10^{-5}	3.2×10^{-5}	(Kiselyov et al., 2009)

It is thought that the early stages of endocytosis are clathrin-mediated; however, due to the limited availability of clathrin, this mechanism quickly becomes saturated. Once saturated, slower endocytosis mechanisms take over. To describe this process, our rate law describing receptor internalization contains two parts:

$$v = \overbrace{k_c \cdot \left(\frac{[pSCD]}{K_M + [pSCD]}\right)}^{\textit{Clathrin-mediated term}} + k_{nc} \cdot [pSCD] \qquad \text{(E21)}$$

The first is the saturable, clathrin-mediated component, whose rate constant k_c indicates values described in Table SI3, above. The second part is a first order reaction whose rate constant k_{nc} is set to the "other+other" internalization rate for all receptor dimers. The term $pSCD$ denotes the concentration of the phosphorylated signaling competent dimer and K_M is an effective Michaelis constant.

2.3 Proliferation and growth submodel

About. This submodel was largely build from scratch, with much inspiration from prior models (Birtwistle et al., 2007; von Kriegsheim et al., 2009; Nakakuki et al., 2010). The model depicts the binding of adaptor proteins to phosphorylated receptor tyrosine kinases, leading to the activation of two major signaling pathways—the ERK and AKT pathway. Once phosphorylated,

receptors can become internalized and are known to signal to the ERK pathway from early endosomes (Burke et al., 2001; Gregory and Hann, 2000; McKay and Morrison, 2007). Many receptors then enter into late endosomes and are degraded; others are de-phosphorylated and recycled back to the plasma membrane (Resat et al., 2003). The activation of ERK (resulting in a cascade from Ras to Raf to MEK to ERK) results in the upregulation, phosphorylation and stabilization of cFos (with p90RSK), promoting it to bind cJun, forming AP1; active ERK also mediates the transcriptional upregulation of cFos (Murphy et al., 2002), dual-specificity phosphatases (DUSPs) (Brondello et al., 1997), and Sprouty proteins (Ozaki et al., 2001). There exists a positive feedback at the level of cJun, as AP1 (phosphorylated cFos bound to cJun) is known to transcriptionally upregulate cJun (Angel et al., 1988). In addition to the negative feedback ERK induces via DUSP phosphatases and Sprouty proteins, other mechanisms of negative feedback include ERK regulation of CRaf (Dougherty et al., 2005), receptor tyrosine kinases (Li et al., 2008), and Grb2-SOS (Chen et al., 1996) via direct (and/or indirect) phosphorylation.

The activation of AKT can induce the nuclear localization of β-Catenin through GSK3β, which is known to upregulate cMyc (He et al., 1998). Active AKT (along with ERK) can also activate mTOR signaling via the TSC complex. TSC complex inhibition by AKT leads to the upregulation of mTORC1, which is known to increase translation globally and regulate cell growth. This is accomplished by mTORC1-mediated phosphorylation and inhibition of EIF4E-BP1 (Manning and Cantley, 2003), causing it to dissociate from and make available EIF4E, as well as its phosphorylation and activation of p70S6K (Fingar et al., 2002), which, when active, drives ribosome synthesis. mTORC1 is also thought to exhibit negative feedback on PI3K (Carracedo and Pandolfi, 2008). As was done for the RTK reactions, here we also assume association reactions to be slower than diffusion limited (0.001 $nM^{-1}s^{-1}$). When tuning was needed (to match simulations to experimental observations), we principally altered dissociation rate constants and catalytic constants. As a baseline assumption, we set dissociation rate constants to 0.1 s^{-1} and catalytic constants for kinase/phosphatase reactions to 1 s^{-1} (Birtwistle, 2015). All rate constant values, and sources for each rate constant, for the proliferation and growth submodel (as well as for the RTK submodel) can be found in Supplementary Table 2.

Pathway preferences. Once intracellular tyrosine residues of signaling-competent dimers become phosphorylated, adaptor proteins are recruited, each of which leads to the activation of distinct downstream signaling pathways. The extent to which a receptor activates one pathway over another depends, in part, on the number of binding sites it possesses for adaptor proteins. In our model we consider three different adaptor proteins: Grb2 (and implicitly Shc), PLC-γ, and PI3K (composed of regulatory and catalytic subunits). We determined how many binding sites for each of these three adaptor proteins existed in each receptor based on known domains within each receptor sequence. To do this, we used ScanSite (http://scansite3.mit.edu) set to "medium" stringency setting (Obenauer et al., 2003). For Grb2 we summed across domains corresponding to Grb2 and Shc. For PI3K we summed across domains corresponding to PI3K SH2 and SH3 domains. For PDGFR we took the average between the number of sites on PDGFR-A and PDGFR-B isoforms for each adaptor. For FGFR, we averaged across FGFR1 and FGFR2 isoforms. For IRS, we averaged across IRS1 and IRS2 isoforms. These factors, depicted in Table SI4, served as multipliers in the rate laws describing the binding of phosphorylated signaling competent dimers to adaptor proteins.

Table SI4. Number of adaptor binding sites on each receptor tyrosine kinase.

**Receptor**	**Grb2**	**PI3K**	**PLC-γ**
EGFR	5	1	2
ErbB2	4	3	2
ErbB3	3	9	1
ErbB4	6	3	1
Met	1	3	2
PDGFR	0.5	3	2.5
FGFR	4	8	3
IGFR	1+3*	0+10.5*	0+1.5*
INSR	2+3*	2+10.5*	1+1.5*

* Added number of sites when receptor is bound to an IRS molecule.

In addition, the affinity of adaptor proteins for phosphorylated tyrosine residues (Table SI5) was taken from a literature source that previously compiled data from multiple experimental studies (Birtwistle, 2015):

Table SI5. Affinity (K_D) of adaptor proteins for phosphorylated tyrosine residues on receptors.

	Grb2	PI3K	PLC-γ
K_D	2600nM	70nM	510nM

Basal fluxes. Even when cells are serum-starved, basal levels of signaling exist. We encode this basal flux by modeling basal activity of Ras (first-order Ras-GDP to Ras-GTP, and vice versa) and PI-3K (first-order PIP_2 to PIP_3, and vice versa). These propagate basal signals through the ERK and AKT (and mTOR) pathways. Normally these signals are between 1-10% of the total activation amplitude.

Dose and dynamic pathway responses. We stimulated MCF10A cells with various doses and combinations of EGF and insulin and measured pERK, pAKT, pEIF4E-BP1 (mTORC1 proxy) and cyclin D at various time points post-stimulation via μ-Western blot. We focused on EGF and insulin because we observed a synergistic interaction between the two growth factors and desired to investigate further (see Figure 6 in main text). Also, these two growth factors are critical for the growth of MCF10A cells. We tuned model parameters to match the dose and dynamic response of measured pERK, pAKT, and pEIF4E-BP1 levels at 0, 5, 30, 180, and 360 minutes post-stimulation (see Figure 3B-D in main text). In addition, we ensured that the proportions of simulated cyclin D levels at 6 hours post-stimulation for EGF (10nM), insulin (10μg/mL), and EGF plus insulin matched those measured via μ-Western blot (see Figure 6C in main text).

Incorporating inhibitors. Because many of our research questions revolved around how ERK and AKT activity related to various cell fate outcomes, several of our experimental perturbations included the inhibition of MEK (just upstream of ERK) and AKT. Our AKT inhibitor, MK2206, is an non-ATP competitive allosteric inhibitor, thought to prevent binding of the kinases that phosphorylate and activate AKT (Cherrin et al., 2010). Thus, we decided to model it binding to the non-phosphorylated form of AKT and inhibiting its activation. Conversely, the MEK inhibitor we use, PD-0325901, is thought to bind to the ATP-binding domain of MEK,

preventing its ability to phosphorylate its targets (Barrett et al., 2008). We decided to code this as the MEK inhibitor binding to the doubly phosphorylated form of MEK.

2.4 DNA damage submodel

About. The DNA damage submodel describes the activation of ATR and ATM by single- and double-stranded DNA breaks, respectively. Single-strand breaks also at this juncture encapsulate point mutations that must be excised and replaced with the correct base pair by repair enzymes (although future models could take a finer grained approach). The activation of ATR and ATM leads to the production of sustained versus oscillatory p53 dynamics, as is known to occur in mammalian cells (Batchelor et al., 2011). The activation of p53 leads to the production of MDM2 and WIP1, both of which serve as negative feedback regulators on p53 activation—MDM2 is an E3 ubiquitin ligase that induces degradation of active p53 (Haupt et al., 1997); WIP1 has a negative feedback on ATM (Shreeram et al., 2006). DNA repair enzymes can repair DNA damage; here, we encode activities of BRCA2 (double-stranded breaks), MSH6 (single-stranded breaks), and MGMT (single-stranded breaks). p53 can also bind to, and be sequestered, by MDM4 (Bessette et al., 2015).

Converting DDEs to ODEs. The original DNA damage model from the Lahav lab (Batchelor et al., 2011) originally contained delay differential equations, which served to model a delay between p53 activation and the upregulation of WIP1 and MDM2. We replaced the delay differential equations with a series of 20 first-order ordinary differential equations that are meant to represent the mechanistic steps that are implied by the original delay (e.g. p53 biding to DNA, recruiting co-transcription factors, synthesizing RNA, etc.). These equations were of the form:

$$\frac{dX}{dt} = \frac{Y}{\tau} - \frac{X}{\tau} \quad \text{(E22)}$$

Here, Y would be the next variable in the cascade. We estimated the τ parameters using a genetic algorithm, implemented using the `ga` function in Matlab, which gave a near-perfect fit between the original model and ours (Figure SI1, below)

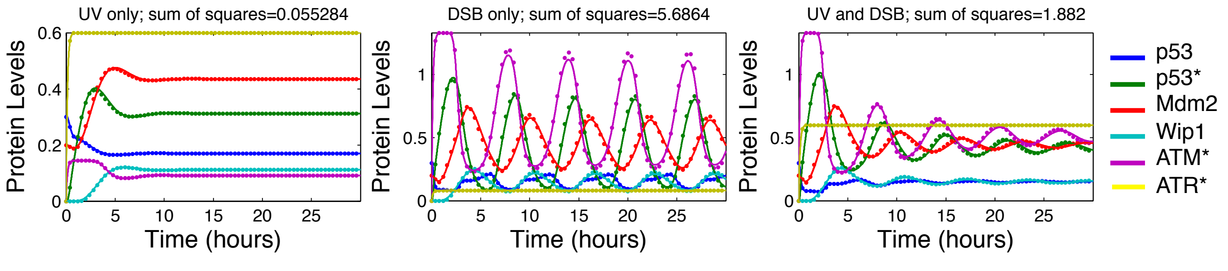

Figure SI1. Simulation results comparing original delay differential equation model (Batchelor et al., 2011) displayed with dots and ordinary differential equation model fit displayed with lines in same color.

DNA damage induction by Etoposide. We also altered the way in which DNA damage was induced in the model. The original Bachelor et al. (2011b) model possessed an on or off switch for DNA damage. We decided to encode DNA damage as a continuous variable, one for single

stranded breaks (SS) and one for double stranded breaks (DS). Here, we decided to focus on Etoposide as the principal DNA damage stimulus, a clinically approved inhibitor of topoisomerase II. Topoisomerase II is an enzyme that aids in the uncoiling of DNA during replication. Importantly, Etoposide is only lethal to cells actively traversing through S-phase (see Figure 4 in main text). We used the following equation to capture these properties:

$$v_{DD} = \left(k_{basal} + k_E \cdot \left(\frac{[Etop]}{[Etop] + KE_{50}}\right)\right) \cdot \left(\frac{(C_A + C_E)^n}{(C_A + C_E)^n + {KS_{50}}^n}\right) \quad \text{(E23)}$$

Here, v_{DD} is the rate of DNA damage accumulation (with equal contributions to the creation of single- and double-stranded breaks), k_{basal} is the basal DNA damage rate meant to reflect damage in the absence of an overt stimulus (estimated during initialization, see Chapter 3.4), k_E is the maximal rate of Etoposide-caused DNA damage for which KE_{50} is the half-maximal (estimated to fit model to cellular measurements), and C_A and C_E are the levels of active cyclin A/Cdk2 and cyclin E/Cdk2 in the model (these are the cyclins upregulated during S-phase and bound to their cognate cyclin dependent kinases).

DNA damage repair. For the repair of DNA damage, we incorporated DNA repair enzymes that could repair DNA in a concentration-dependent manner. The incorporation of DNA repair enzymes is important because they are frequently mutated in cancer. These genes included BRCA2 (double strand break repair), MSH6 and MGMT (both single strand break or point mutation repair). Differential equations describing the change of DNA damage over time were defined as follows:

$$\frac{d[DS]}{dt} = v_{DD} - k_{ds} \cdot [BRCA2] \cdot [DS] \quad \text{(E24)}$$

$$\frac{d[SS]}{dt} = v_{DD} - k_{ss1} \cdot [MGMT] \cdot [SS] - k_{ss2} \cdot [MSH6] \cdot [SS] \quad \text{(E25)}$$

Initial conditions. The initial conditions of the DNA damage submodel were taken directly from their original source, except for the levels of repair enzymes, which came from proteomics measurements. Species in the original model possessed arbitrary units. We found the best approximation of these to an absolute unit of measurement to assume they represented units of µM, which we then converted to units of nM, scaling all rate constants accordingly.

"Digital" p53 dynamics. As mentioned in the original text, p53 dynamics are thought to be "digital" instead of "analog." Namely, that increases in DNA damage are thought to increase the number of p53 pulses in a single cell without inducing a change in pulse amplitude or width (Lahav et al., 2004). To achieve this behavior, we needed to render the p53 response to DNA damage even more ultrasensitive. The original model already possessed a cooperative hill function for the activation of p53 by ATR and ATM. We added an additional cooperative hill function for the activation of ATM and ATR by DNA damage. The rate laws describing the activation of ATM and ATR are now as follows:

$$v_{ATM} = b_{ATM} \cdot \left(\frac{DS^n}{{DSK_{50}}^n + DS^n}\right) \quad \text{(E26)}$$

$$v_{ATR} = b_{ATR} \cdot \left(\frac{SS^n}{{SSK_{50}}^n + SS^n}\right) \quad \text{(E27)}$$

Here, b_{ATM} and b_{ATR} are rate constants and DS and SS are quantities of double- and single-stranded breaks in DNA. DSK_{50} and SSK_{50} (both set to 2nM; n=20) are EC_{50} concentrations for DS and SS, respectively (half-maximal concentrations required to induce a full response). This ensured a switch-like p53 response to DNA damage once a certain threshold of DNA damage was achieved. (In the original model, there was a DNA damage multiplier following the b_{ATM} and b_{ATR}, with a value of either one or zero, serving as an on or off switch for DNA damage.) The rate of DNA damage repair also played an important role in ensuring that the number of p53 pulses increased with increasing DNA damage. As DNA damage is repaired and its levels drop beneath the threshold needed for activation of ATR and ATM, the activation of p53 is eventually ceased; however, while DNA damage remains above the threshold, highly regular p53 pulses persist (see Figure 3F and S3E in main text). Thus, in addition, we also manually fit several parameters of DNA repair (k_{ds}, k_{ss1}, and k_{ss2} from Equations E24 and E25) to enable this behavior.

2.5 Apoptosis submodel

About. The apoptosis submodel depicts the activation of initiator caspases (caspase 8 and 10) by TRAIL through the death receptors (DR4/5), which leads to the activation of executioner caspases (caspases 3 and 7), leading to cell death (Albeck et al., 2008). Initiator caspases can also activate a mitochondrial dependent pathway, driven by caspase 8 directed cleavage of Bid, which activates Bax. Active Bax can then oligomerize on the mitochondrial membrane and form pores leading to the release of cytochrome C. The release of cytochrome C from the mitochondria prompts the formation of the apoptosome, which activates caspase 9 (regulated by levels of XIAP and Smac). Caspase 9 can then feed back onto the activation of executioner caspases. There exists a positive feedback loop between initiator and executioner caspases (caspase 8 → caspase 3 → caspase 6 → caspase 8), which serves to create an all-or-nothing response once the appropriate levels of caspases have become activated (Eissing et al., 2004). Executioner caspases drive the digestion of many critical cellular components, which results in cell death. PARP, a DNA repair enzyme, is one target of caspase 3, and cleaved PARP (cPARP) is commonly readout for apoptosis (Tewari et al., 1995).

There are also intracellular modes of apoptosis signaling, through the regulation of pro- and anti-apoptotic proteins by phosphorylated ERK, phosphorylated AKT, PUMA, and NOXA. We added a few proteins to the original Albeck et al. (2008) model in order to describe intrinsic apoptosis signaling. We added BAD (pro-apoptotic Bcl2 protein, phosphorylated by ppERK and ppAKT) and BIM (pro-apoptotic Bcl2 protein, phosphorylated by ppERK and transcriptionally upregulated via ppAKT/FOXO). In addition, we added a bidirectional translocation reaction between cytoplasmic and mitochondrial forms of Bcl2 as it was not included in the original model.

Sensitivity and robustness of apoptosis submodel. The original apoptosis model created by Albeck et al. (2008) was designed to model extrinsic cell death processes in response to TRAIL over relatively short time scales. Because of this, they did not need to incorporate synthesis and degradation processes, or basal caspase activities. Because we are modeling over longer time scales, we added such mechanisms, which also allow robustness to small amounts of death signal. One mechanism by which the cell can do this is by rapidly degrading active, pro-apoptotic proteins (e.g. active caspases). We increased the degradation rates for several active pro-apoptotic proteins by several fold above their inactive form. Degradation of active TRAIL receptor complex was increased 1000 times and the degradation rates of active caspase 8, 3, 6, tBid, and active Bax were increased by 100 times above their inactive forms. Now, when a subthreshold amount of caspases becomes active, they are rapidly degraded, and the cell can return to homeostasis. Synthesis of all mRNAs and proteins in the model was incorporated via the expression submodel.

Once the MCF10A levels for apoptosis proteins were set, several rate constants needed to be modified to reduce the sensitivity of the model without losing the all-or-nothing response once a threshold of apoptosis signaling has been achieved. We made alterations to the reactions as described below in Table SI6. These changes are certainly not unique but do result in phenotypic predictions consistent with experimental observations.

Table SI6. Changes made to rate constants in original apoptosis model by Albeck et al (2008).

Reaction	Fold change
Caspase 6 biding pro-caspase 8	1/1000
Caspase 3 binding pro-caspase 6	1/100000
Caspase 8 binding pro-caspase 3	1/100000
Caspase 8 binding Bid	1/10000
Caspase 3 binding PARP	1/10000
Bax dimerization	100000
Bax2 (dimer) dimerization	100000
Bax2 (dimer) dissociation	10000
Bax4 (4-subunit protein) dissociation	10000
Apoptosome cleaving pro-caspase 3	1/10000
Caspase 8 cleaving Bid	1/10000
koff for open pores	1000
Catalytic constant for open pores	1/100
Ligand-bound TRAIL receptor becoming active	1/10

Intracellular apoptosis signaling. We desired that the model incorporate intracellular modes of apoptosis signaling, for which we required basal pro-apoptotic signaling. Intracellular modes of apoptosis are thought to rely primarily on the regulation of pro- and anti-apoptotic Bcl2 proteins. Anti-apoptotic Bcl2 proteins are thought to "keep the brakes" on cell death, releasing their inhibition of pro-apoptotic proteins only when an intracellular death signal is initiated or a pro-survival signal is abrogated. We reasoned that for this to be possible, a basal level of death signaling would have to be propagated through the pathway. This would lead to the creation of subthreshold levels of pro-apoptotic proteins (e.g., tBid), which would be sequestered by anti-apoptotic Bcl2 proteins until a death signal of sufficient magnitude and duration was received. To accomplish this, a basal amount of pro-caspase 8 cleavage was incorporated. This propagated

a low amount of death signaling through the entire apoptosis pathway. The rate of basal caspase 8 cleavage was estimated as part of the initialization procedure (below), ensuring that it by itself did not cause apoptosis. In order to approximate the levels of cell death for serum-starved cells over a 72-hour time course, we increase the affinity of BIM for Bax by a factor (x5), post-initialization.

2.6 Cell cycle submodel

About. The cell cycle submodel is initiated by the upregulation of cyclin D mRNA by AP1 and cMyc. Cyclin D becomes active by binding to either Cdk4 or Cdk6 cyclin dependent kinases. Active cyclin D/Cdk4-6 phosphorylates Rb to de-repress E2F transcription. Subsequent upregulation of E2F causes the further upregulation of cyclin D and initiation of cyclin E and cyclin A induction (which bind to Cdk2 to become active), marking the beginning of S-phase and lasting into G2 phase. Finally, the activation of cyclin B/Cdk1 marks the beginning of mitosis, which, when complete, returns the cells to G1 phase.

Cell cycle equations are taken directly from their original source (Gérard and Goldbeter, 2009). We made a few changes to the original model. We removed the equations related to "ATR/Chk1 DNA replication checkpoint" except for the equations describing the upregulation of Chk1 in response to ATR (native to the DNA damage response submodel). We also removed the production of AP1 by growth factors, which is something that is now captured by the proliferation and growth submodel (through the formation of phosphorylated c-Fos bound to c-Jun).

Initial conditions. Because the original cell cycle model possessed intrinsic synthesis and degradation reactions, which we did not alter, we ran the model to a steady state in the absence of cycling and used these final (steady-state) species concentrations as the initial conditions. In addition, this submodel did not need mRNA quantities, as its synthesis reactions were already defined and we did not allow stochastic gene expression to affect it (see more below). We scaled all units from the original cell cycle model (species and rate constants) down by a factor of 10 to better agree with parameters from MCF10A cells.

Cell cycle entry driven by cyclin D mRNA. As described above, we removed the original growth factor terms and replaced them with the AP1 encoded by the Proliferation and Growth submodel. AP1 and MYC served as the primary proliferative inputs into the cell cycle model, whose levels cooperate to drive synthesis of cyclin D mRNA. The production of cyclin D above a certain threshold pushes the system beyond the "restriction point", prompting the cascade of events initiating of the cell cycle and driving it to completion. Because flow cytometry data indicated that inputs from both ERK and AKT pathways were necessary for S-phase to proceed (Figure 6A), we modified the general form of the transcription equation (Equation E12) for cyclin D to reflect a need for both AP1 *and* cMyc (as opposed to AP1 *or* cMyc).

$$v_{bm} = k_{leak} \cdot g^{*} + k_{max} \cdot \left(\frac{\left(\frac{[TA_{AP1}]}{kA_{50AP1}}\right)^{na_{AP1}}}{1 + \left(\frac{[TA_{AP1}]}{kA_{50AP1}}\right)^{na_{AP1}}} \right) \cdot \left(\frac{\left(\frac{[TA_{MYC}]}{kA_{50MYC}}\right)^{na_{MYC}}}{1 + \left(\frac{[TA_{MYC}]}{kA_{50MYC}}\right)^{na_{MYC}}} \right) \cdot g^{*} \quad \text{(E28)}$$

Here, TA_{AP1} and TA_{MYC} are the levels of transcriptional activators AP1 and MYC, respectively, and kA_{50AP1} (estimated to be 1.25 nM) and kA_{50MYC} (estimated to be 450 nM) are the half maximal concentrations of AP1 and MYC, respectively, and na_{AP1} and na_{MYC} (both set to n=3) are hill coefficients for AP1 and MYC induction, respectively. These parameters were manually estimated in order to match simulations to experimental data in terms of cyclin D induction (μ-Western blot data; Figure 6C) and percentage of cycling cells (BrdU flow cytometry data; Figure 6A) in response to EGF (10nM), Insulin (10μg/mL), and EGF + Insulin.

Order and timing of cyclin/cdk complexes. Importantly, this cell cycle model replicated the proper order and timing of cyclin/cdk complex oscillations (depicted in Figure SI2, below). As cyclin D becomes upregulated in G1 phase and binds to its cognate cdks, cdk 4 and 6, it provokes the phosphorylation of Rb, causing Rb to release its inhibition of E2F synthesis. An increase in E2F leads to the production of cyclin E and cyclin A, which define the entry into S- and G2-phase. These then lead to the activation of CDC25, the phosphatase responsible for activating cyclin B/cdk1, which defines entry into mitosis (M-phase). Post-mitosis, cyclin B/cdk1 levels return to baseline and mark re-entry into G1 phase or a possible exit into G0 phase if cyclin D levels have dropped considerably during cycling.

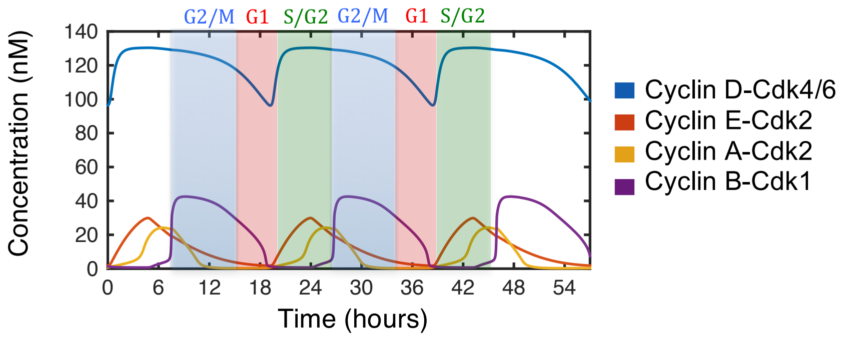

Figure SI2. Timing and order to cyclin/cdk complexes and their correspondence with cell cycle stage.

Cell cycle duration
The simulated duration of one cell cycle event for cells treated with growth factors was approximately 20 hours, which is typical for a mammalian cell. This is also the approximate cell cycle duration measured for MCF10A cells (Albeck et al., 2013).

Inhibition of cycling by p21. Damage to DNA causes upregulation of p53, which is known to upregulate p21 (Riley et al., 2008), a potent cyclin dependent kinase inhibitor (CKI), which can bind to and sequester all cyclin/cdk complexes (Abbas and Dutta, 2009) leading to cell cycle arrest. In order to verify if our model could reproduce this qualitative behavior, we simulated DNA damage while cell cycle oscillations were present (cell cultured in growth factors) and looked for cell cycle arrest. As is clear from Figure SI3, below, upregulation of p21 results in the ceasing of cyclin oscillations and interrupts cell cycle progression. Also, when DNA damage is repaired (by DNA repair enzymes), and p53 activation returns to baseline, the cell is able to re-enter the cell cycle.

Unresolved emergent fragility of cell cycle to expression noise. As described above, every gene product in our model is linked to stochastic gene switching and mRNA production/degradation, giving rise to temporal fluctuations in protein levels. When we attempted to link the cell cycle submodel to stochastic gene expression, we obtained non-sensical simulation results, including rampant spontaneous cycling in the absence of Cyclin D induction, spurious expression of the various cyclins with incorrect ordering, and a lack of regular frequency, amplitude and duration of cyclin peaks. This fragility of cell cycle models to stochastic gene expression-like noise is documented by previous studies (Kar et al., 2009). This observation implies there are as-yet undiscovered cellular mechanisms that provide natural robustness of the cell cycle to gene expression (or other) noise, solving which is outside the scope of this manuscript. Therefore, we retained a deterministic formulation of the cell cycle sub-model.

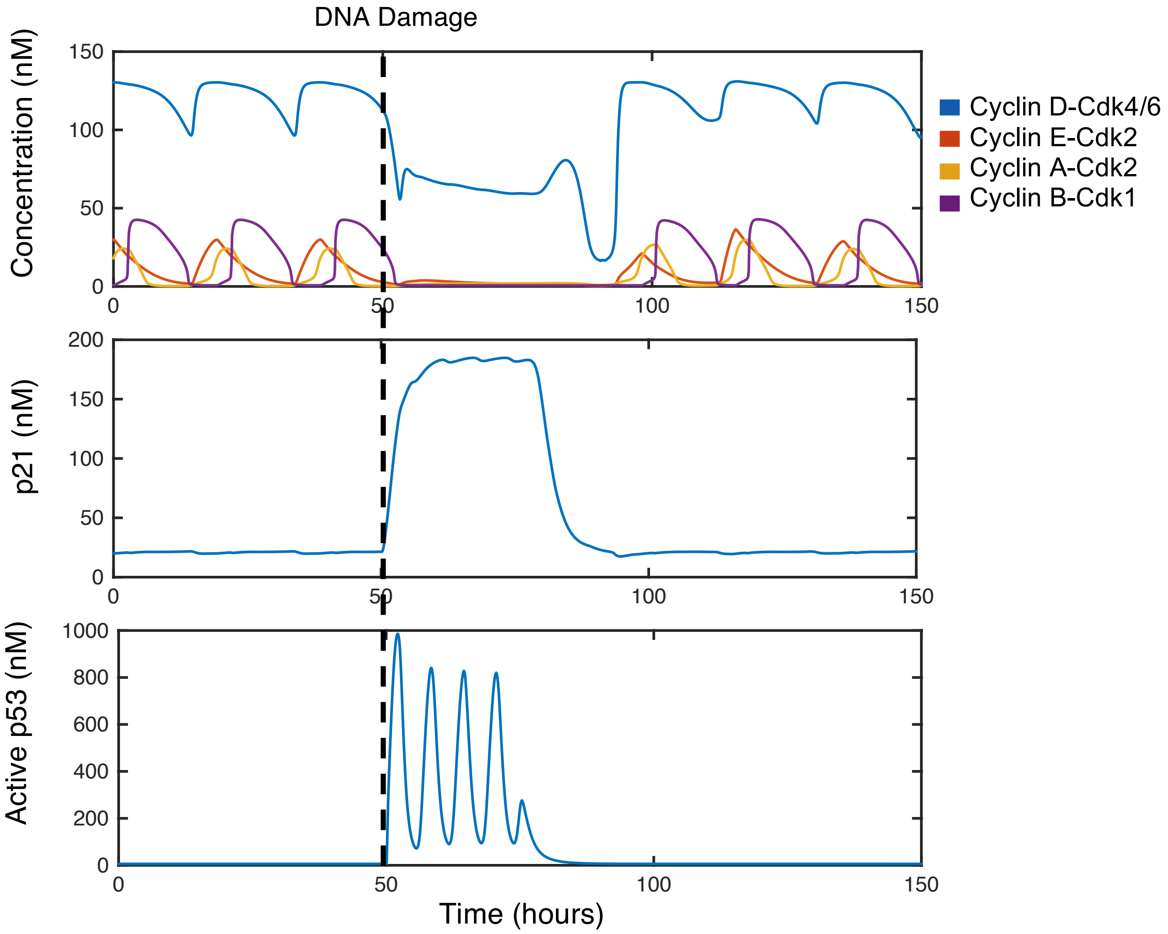

Figure SI3. DNA damage is induced at 50 hours for a cycling cell. The activation of p53 (bottom) causes upregulation of p21 (middle), which arrests the oscillatory behavior of cyclic/cdk complexes (top).

2.7 Submodel integration

Links between submodels were created based on well-studied mechanistic interactions that are known to exist between proteins in each submodel (see Figure S1 for a detailed mechanistic schematic).

Survival Signaling—Proliferation and Growth to Apoptosis. The primary species that mediate survival signals in the model are active ERK and Akt kinases (ppERK and ppAkt). ERK is known to phosphorylate the pro-apoptotic protein BIM, inactivating its ability to active Bax (Hübner et al., 2008). AKT is known to phosphorylate FOXO (Zhang et al., 2011), which when phosphorylated translocates into the nucleus to induce transcription of target genes. These target genes include receptor tyrosine kinases (see below) and BIM (Gilley et al., 2003), which can active Bax. Both ERK and AKT are known to phosphorylate BAD (Fang et al., 1999; del Peso et al., 1997), which renders it inactive and unable to bind to, and sequester, anti-apoptotic Bcl2 proteins.

G0/G1 Transition—Proliferation and Growth to Cell Cycle. Signal flux through the ERK and AKT pathways can also induce the cell cycle by upregulating cyclin D. Specifically, cyclin D is upregulated by AP1 (Shaulian and Karin, 2001) (phosphorylated cFos in complex with cJun) and cMyc (Bouchard et al., 1999), induced by the ERK and AKT pathways, respectively. Moreover, AP1 induces transcription of cJun, leading to positive feedback, which further amplifies AP1's influence on cyclin D (Angel et al., 1988).

Intrinsic Death—DNA Damage to Apoptosis. In a non-transformed cell, the induction of DNA damage promotes apoptosis if the damage is not repaired in a timely fashion (Roos and Kaina, 2006). The cell does this to protect against the potential for developing disease, such as cancer, as a result of the DNA damage. Here, we allow active p53 to upregulate PUMA (Nakano and Vousden, 2001) and NOXA (Oda et al., 2000), both pro-apoptotic proteins that bind to, and sequester, anti-apoptotic Bcl2 proteins.

Cell Cycle Arrest—DNA Damage to Cell Cycle. When a non-transformed cell sustains DNA damage, it arrests the cell cycle in order to prevent the spread of potentially deleterious mutations to daughter cells. This is thought to occur primarily through the transcriptional upregulation of p21 by active p53 (Riley et al., 2008). p21 is a cyclin dependent kinase inhibitor which binds to and inactivates all cyclin/cdk complexes (Abbas and Dutta, 2009). The other mechanism that we encode in our model is the upregulation of Chk1 by active ATR (Hekmat-Nejad et al., 2000), which is activated primarily in response to single-stranded breaks. Chk1 promotes cell cycle arrest by phosphorylating CDC25 proteins, which targets it for rapid degradation. Chk1 can inhibit all CDC25 isoforms (Karlsson-Rosenthal and Millar, 2006). CDC25 are phosphatases that activate many cyclin/cdk complexes. For example, it removes an inhibitory phosphate group from cyclin B/cdk1, rendering it fully active which promotes the initiation of mitosis.

Feedback on Survival Signaling—Proliferation and Growth to RTK. As is commonly seen across numerous signaling pathways, activation is usually followed by a phase of deactivation or negative feedback, preventing chronic and potentially deleterious pathway activation. Signaling through the ERK pathway is known to affect receptor tyrosine kinases by phosphorylating them on specific sites that blunt their ability to signal downstream (Li et al., 2008), providing a mechanism of negative feedback. In our model ERK phosphorylates and inactivates the ErbB family members (ErbB1-4). There are mechanisms of transcriptional feedback as well. Receptor tyrosine kinases are known transcriptional targets of FOXO (we allow FOXO to transcriptionally upregulate all receptor tyrosine kinases in our model), which translocates into the nucleus upon

dephosphorylation; when AKT is active, FOXO is phosphorylated and cytoplasmic (Tzivion et al., 2011; Zhang et al., 2011). Sprouty proteins are transcriptionally upregulated by active ERK, which bind to and inactivate multiple RTKs (Ozaki et al., 2001).

Protein synthesis—Expression to Other Submodels. Synthesis processes are captured by the expression submodel, which controls the production of proteins across most of the submodels (with the exception of the cell cycle, as described above). We model 141 total genes. These genes are translated to create only 102 "protein conglomerates", as some of the gene isoforms perform redundant functions or are experimentally indistinguishable (see Supplementary Table 1 for mapping between proteins and gene isoforms). Functionally redundant isoforms are summed into the final protein amount, p, which we term a conglomerate:

$$\frac{dp}{dt} = \sum_{i=1}^{n} v_{bp_i} - k_{dp} \cdot p \qquad \text{(E29)}$$

Here, v_{bp_i} is the translation rate for every functionally redundant isoform, i, for protein, p. For example, the ERK protein is functionally composed of two main isoforms: MAPK1 (ERK2) and MAPK3 (ERK1) (Figure 2B). Upon translation, these two isoforms are summed to create the protein conglomerate "ERK" in the model.

Chapter 3

Initialization Procedure

3.1 Initialization rationale, approach, and output

The goal of the initialization procedure is to ensure that the steady state levels for all proteins and mRNAs in the model match what we have measured in serum-starved MCF10A cells. It is also done to capture phenotypic behavior that one would expect for cells in the serum-starved state (e.g. not cycling, dying in small numbers, etc.). The model commences with protein quantities given to species that represent proteins that have been immediately translated from mRNA; that is, they lack any post-translational modifications. However, the levels of post-translationally modified proteins may change when we run the model due to basal activities (e.g. basal Ras, PIP_3, and caspase 8 activity; more below) or other phenomena (e.g. phosphorylation of β-catenin by GSK3-β; formation of heterodimeric complexes). These post-translationally modified forms of proteins may have different degradation rates than their nascent counterparts. Thus, if run to steady state, the total amount of a given protein may no longer equal what was measured via proteomics.

To correct for this, we increase or decrease synthesis rates by altering gene-specific translation rate constant (k_{TL}) values. We do this using an iterative approach. Every time we introduce a new element to the system (e.g. such as different basal activities), we run the model to an effective steady state in the absence of growth stimulus (1000 hours) and calculate a ratio between simulated (sum across all species that possess a given protein) and measured protein levels. We then multiply the translation rate constant (k_{TL}) by this ratio and re-run the simulation. We repeat this process until simulations are within 1% of experimental data. All simulations during initialization are performed deterministically.

The process of initializing transcription is described below under subheading TARs, however the goal is the same (post-initialization mRNA levels should match mRNA-seq measurements). Quantification of the final initialization results can be found in main text Figure 2E. Overview of initialization process can be found in Figure SI4, below.

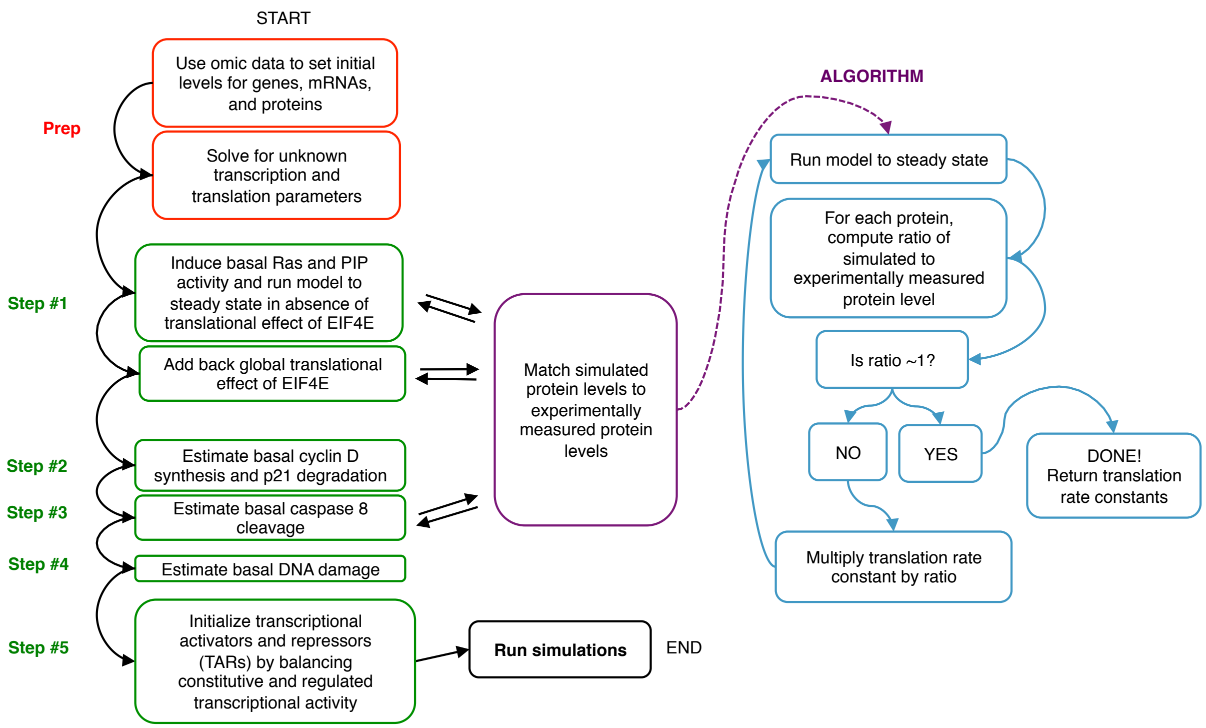

Figure SI4. Schematic depicting overview of initialization process.

3.2 Step 1: Basal Ras and PIP activities

The first step of initialization begins with all post-translationally modified forms of proteins set to zero. During this step, basal activity fluxes are turned on. Basal fluxes through the ERK and AKT pathways are encoded by two activities: (*i*) basal Ras activation and inactivation reactions (e.g. intrinsic RasGTPase activity) and (*ii*) basal PIP2 phosphorylation and dephosphorylation reactions. Here, we employ the iterative approach described above to alter the translation rate constants (k_{TL}) until steady-state protein levels match experimentally-measured protein levels. Importantly, during this step we prohibit EIF4E from affecting the overall translation rate. We do this because basal fluxes as well as the binding of EIF4E to mRNAs induce large changes in free EIF4E levels. Because EIF4E can alter translation rates globally, rapid changes in its levels make it difficult to approximate a steady state for each species. We accomplish this by keeping the free EIF4E levels in the translation equation from Equation E12 set to total EIF4E levels (as measured by proteomics), so that changes in free EIF4E cannot affect the overall translation rate. This converts the translation equation to:

$$v_{bp\_step1} = k_{TL} \cdot m_{Total}{}^{*} \frac{[EIF4E_{Total}]}{k_D + [EIF4E_{Total}]} \quad \text{(E30)}$$

Once good agreement between simulation and experiment is achieved, a final free EIF4E quantity is defined. Using this quantity, we calculate a new v_{bp} for each gene and multiply the translation rate constants (k_{TL}) by the ratio between the v_{bp_step1} and v_{bp}.

3.3 Step 2: Basal cyclin D synthesis and p21 degradation

This initialization step is performed in order to estimate two parameters in the cell cycle submodel. As mentioned before, synthesis and degradation reactions for the cell cycle submodel are intrinsic to that submodel. We also keep the initial concentrations from the original model, with the exception of cyclin D and p21, which we set using our proteomics data. To set initial cyclin D levels we estimate a basal cyclin D synthesis constant. The rate equation describing cyclin D synthesis is as follows:

$$v_{CYCD} = k_{cd1} + k_{cd2} \cdot [E2F] \cdot \left(\frac{K_{i7}}{K_{i7} + pRB}\right) \cdot \left(\frac{K_{i8}}{K_{i8} + pRBp}\right) \quad \text{(E31)}$$

The first term, k_{cd1}, is a zero-order synthesis for cyclin D driven by basal levels of transcriptional activators (Gérard and Goldbeter, 2009). The second term describes cyclin D synthesis as driven by E2F levels and inhibited by Rb levels. In this initialization step, we estimate the value of k_{cd1}. We employ a similar iterative approach as described above until the total amount of cyclin D in the system is equal to the amount measured via proteomics. Importantly, we ensure that this amount of cyclin D does not induce cell cycle oscillations, as the cells do not cycle in the serum-starved state. To set p21 levels we estimate the degradation rate constant for p21, which follows first-order degradation kinetics. We do this in the same iterative manner as for cyclin D.

3.4 Step 3: Basal caspase 8 cleavage

The ability of an internal pro-apoptotic stimulus (e.g. PUMA/NOXA upregulation by p53) to induce apoptosis depends in large part on the negative regulation of anti-apoptotic proteins. This is only possible if there is a basal flux of death signaling through apoptotic pathways. Indeed, if there was no basal death signaling, there would be no pro-apoptotic proteins for the anti-apoptotic Bcl2 proteins to bind, and the inhibition of anti-apoptotic Bcl2 would do nothing to induce cell death. Basal death signaling could be the result of basal caspase activity, which is known to occur in the absence of an overt death stimulus (Gdynia et al., 2007; Marini et al., 2014). We chose to add a basal, first-order caspase 8 cleavage reaction to simulate this effect. Prior to this initialization step, the basal caspase 8 cleavage rate is set to zero. In this initialization step, we iterate through increasing values (log-spaced) of the caspase 8 cleavage rate constant until apoptosis occurs within a 1000-hour simulation time window. We choose the value that is one increment lower than the value that caused apoptosis. Finally, because the introduction of this rate may render a mismatch between the quantities of various proteins in the model and their experimentally measured values, we also re-approximate translation rate constants (as above) until all model protein levels match their experimentally measured values. We then test to make sure the cell still undergoes apoptosis as previously.

3.5 Step 4: Basal DNA damage

Due to insults from the environment as well as internal stressors, there exists a low, basal amount of DNA damage inside living cells even when no explicit insult is present. The effects of basal DNA damage are enhanced during S-phase, as the DNA is uncoiled from histones and becomes more venerable to insult. In addition, because DNA is being shuffled so much during replication,

breaks are more likely to occur. However, this basal DNA damage plus replicative stress should not be enough to induce a p53 response on average (i.e. deterministic regime). The goal of this initialization step is to calculate a level of basal DNA damage activity (described by the term k_{basal}, in Equation E23) such that (*i*) the total amount of active p53 is between 1-5% of its total quantity and that (*ii*) a full p53 response is not elicited by the basal activity. To do this, we estimate a value for k_{basal} such that 1-5% of p53 is active and such that the quantity of single- and double-stranded DNA breaks does not exceed its half-maximal effective concentration (SSK_{50} and DSK_{50}, respectively, from Eqs E5 and E5, below) needed to induce full activation of ATR and ATM, respectively. The rate law for the production of DNA damage (v_{DD}) is defined above in Equation E23, and placed here for convenience:

$$v_{DD} = \left(k_{basal} + k_E \cdot \left(\frac{[Etop]}{[Etop] + KE_{50}}\right)\right) \cdot \left(\frac{(C_A + C_E)^n}{(C_A + C_E)^n + {KS_{50}}^n}\right) \qquad \text{(E23)}$$

Recall the differential equations for double- and single-stranded breaks from above:

$$\frac{d[DS]}{dt} = v_{DD} - k_{ds} \cdot [BRCA2] \cdot [DS] \qquad \text{(E24)}$$

$$\frac{d[SS]}{dt} = v_{DD} - k_{ss1} \cdot [MGMT] \cdot [SS] - k_{ss2} \cdot [MSH6] \cdot [SS] \qquad \text{(E25)}$$

When a cell is in S-phase and untreated with Etoposide, v_{DD} is equivalent to k_{basal} (basal DNA damage rate). Solving Equations E24 and E25 above at steady state for DS and SS gives:

$$DS = \frac{k_{basal}}{k_{ds} \cdot [BRCA2]} \qquad \text{(E32)}$$

$$SS = \frac{k_{basal}}{k_{ss1} \cdot [MGMT] + k_{ss2} \cdot [MSH6]} \qquad \text{(E33)}$$

Thus we ensure that the quantity for DS and SS do not exceed DSK_{50} and SSK_{50} (both set to 2nM) in the following rate laws describing activation of ATM and ATR from above, repasted here for convenience:

$$v_{ATM} = b_{ATM} \cdot \left(\frac{DS^n}{{DSK_{50}}^n + DS^n}\right) \qquad \text{(E26)}$$

$$v_{ATR} = b_{ATR} \cdot \left(\frac{SS^n}{{SSK_{50}}^n + SS^n}\right) \qquad \text{(E27)}$$

Our final active p53 percentage was approximately 2% and this did not induce a full p53 response.

3.6 Step 5: Transcriptional activators and repressors (TARs)

The goal of this initialization step is to ensure that mRNA synthesis rates are in equilibrium with mRNA degradation rates, particularly for mRNAs that have modeled regulation. These transcriptional activators and repressors (TARs) that perform such regulation are the species that transfer information from the deterministic model to the stochastic model. As depicted in Equation E12, above, the differential equation for mRNA production contains the rate of transcription, which possesses a "leak" or constitutively active term plus an "induced" or TAR induced expression term, minus the rate of mRNA degradation. The general form of the differential equation is as follows:

$$\frac{dm}{dt} = leak + induction - degradation \quad \text{(E34)}$$

The rate of mRNA degradation was already known, calculated from mRNA half-lives and mRNA levels as described above. We then simply required that, at steady-state, the rate of transcription was equal to the rate of degradation:

$$leak + induction = degradation \quad \text{(E35)}$$

We first calculated the induction term, which, as depicted in Equation E12, is a function of the levels of TARs, and preset K_{50} values and hill coefficients. The level of each TAR is taken from the fully equilibrated model. K_{50} values and hill coefficients are estimated to (i) ensure that induction plus leak terms do not exceed degradation terms and (ii) to fit the model to experimental results or other empirical observations. For example, the parameters for induction of cyclin D by AP1 and cMyc were estimated to approximate cyclin D protein induction as measured by μ-Western blot and percentage of cycling cells in response to different growth factors as measured by BrdU incorporation flow cytometry experiments. Leak terms are then calculated to balance out the equation; specifically the k_{leak} term from Equation E5. If a gene did not possess any explicitly coded TARs, the induction term was zero, and the leak term was equal to the degradation term.

REFERENCES

Abbas, T., and Dutta, A. (2009). P21 in Cancer: Intricate Networks and Multiple Activities. Nat. Rev. Cancer *9*, 400–414.

Aberle, H., Bauer, A., Stappert, J., Kispert, A., and Kemler, R. (1997). β-catenin is a target for the ubiquitin-proteasome pathway. EMBO J. *16*, 3797–3804.

Albeck, J.G., Burke, J.M., Spencer, S.L., Lauffenburger, D.A., and Sorger, P.K. (2008). Modeling a snap-action, variable-delay switch controlling extrinsic cell death. PLoS Biol. *6*, 2831–2852.

Albeck, J.G., Mills, G.B., and Brugge, J.S. (2013). Frequency-Modulated Pulses of ERK Activity Transmit Quantitative Proliferation Signals. Mol. Cell *49*, 249–261.

Alberts, B. Johnson, A. Lewis, J. Raff, M. Roberts, K. Walter, P. (2008). Molecular Biology of the Cell, 5th Edition.

Alon, U. (2007). An Introduction to Systems Biology: Design Principles of Biological Circuits. Chapman HallCRC Math. Comput. Biol. Ser. *10*, 301.

Alvarado, D., Klein, D.E., and Lemmon, M. a. (2010). Structural Basis for negative cooperativity in growth factor binding to an EGF receptor. Cell *142*, 568–579.

Angel, P., Hattori, K., Smeal, T., and Karin, M. (1988). The jun proto-oncogene is positively autoregulated by its product, Jun/AP-1. Cell *55*, 875–885.

Ballesta, a, Zhou, Q., Zhang, X., Lv, H., and Gallo, J.M. (2014). Multiscale design of cell-type-specific pharmacokinetic/pharmacodynamic models for personalized medicine: application to temozolomide in brain tumors. CPT Pharmacometrics Syst. Pharmacol. *3*, e112.

Barrett, S.D., Bridges, A.J., Dudley, D.T., Saltiel, A.R., Fergus, J.H., Flamme, C.M., Delaney, A.M., Kaufman, M., LePage, S., Leopold, W.R., et al. (2008). The discovery of the benzhydroxamate MEK inhibitors CI-1040 and PD 0325901. Bioorganic Med. Chem. Lett. *18*, 6501–6504.

Batchelor, E., Loewer, A., Mock, C., and Lahav, G. (2011). Stimulus-dependent dynamics of p53 in single cells. Mol. Syst. Biol. *7*, 488.

Bertaux, F., Stoma, S., Drasdo, D., and Batt, G. (2014). Modeling Dynamics of Cell-to-Cell Variability in TRAIL-Induced Apoptosis Explains Fractional Killing and Predicts Reversible Resistance. PLoS Comput. Biol. *10*.

Bessette, D.C., Tilch, E., Seidens, T., Quinn, M.C.J., Wiegmans, A.P., Shi, W., Cocciardi, S., McCart-Reed, A., Saunus, J.M., Simpson, P.T., et al. (2015). Using the MCF10A/MCF10CA1a breast cancer progression cell line model to investigate the effect of active, mutant forms of EGFR in breast cancer development and treatment using gefitinib. PLoS One *10*, 1–25.

Birtwistle, M.R. (2015). Analytical reduction of combinatorial complexity arising from multiple protein modification sites. J. R. Soc. Interface *12*, 20141215.

Birtwistle, M.R., Hatakeyama, M., Yumoto, N., Ogunnaike, B.A., Hoek, J.B., and Kholodenko, B.N. (2007). Ligand-dependent responses of the ErbB signaling network: experimental and modeling analyses. Mol. Syst. Biol. *3*.

Bouchard, C., Thieke, K., Maier, A., Saffrich, R., Hanley-Hyde, J., Ansorge, W., Reed, S., Sicinski, P., Bartek, J., and Eilers, M. (1999). Direct induction of cyclin D2 by Myc contributes to cell cycle progression and sequestration of p27. EMBO J. *18*, 5321–5333.

Bouhaddou, M., and Birtwistle, M.R. (2014). Dimerization-based control of cooperativity. Mol. Biosyst. *10*, 1824–1832.

Brondello, J.M., Brunet, A., Pouysségur, J., and McKenzie, F.R. (1997). The dual specificity mitogen-activated protein kinase phosphatase-1 and -2 are induced by the p42/p44(MAPK) cascade. J. Biol. Chem. *272*, 1368–1376.
Burke, P., Schooler, K., and Wiley, H.S. (2001). Regulation of epidermal growth factor receptor signaling by endocytosis and intracellular trafficking. Mol. Biol. Cell *12*, 1897–1910.
Carracedo, A., and Pandolfi, P.P. (2008). The PTEN – PI3K pathway : of feedbacks and cross-talks. 5527–5541.
Chauvin, C., Koka, V., Nouschi, a, Mieulet, V., Hoareau-Aveilla, C., Dreazen, a, Cagnard, N., Carpentier, W., Kiss, T., Meyuhas, O., et al. (2014). Ribosomal protein S6 kinase activity controls the ribosome biogenesis transcriptional program. Oncogene *33*, 474–483.
Chen, D., Waters, S.B., Holt, K.H., and Pessin, J.E. (1996). SOS phosphorylation and disassociation of the Grb2-SOS complex by the ERK and JNK signaling pathways. J. Biol. Chem. *271*, 6328–6332.
Cherrin, C., Haskell, K., Howell, B., Jones, R., Leander, K., Robinson, R., Watkins, A., Bilodeau, M., Hoffman, J., Sanderson, P., et al. (2010). An allosteric Akt inhibitor effectively blocks Akt signaling and tumor growth with only transient effects on glucose and insulin levels in vivo. Cancer Biol. Ther. *9*, 493–503.
Christoffersen, C.T., Bornfeldt, K.E., Rotella, C.M., Gonzales, N., Vissing, H., Shymko, M., Groffen, J., Heisterkampg, N., Meyts, P. De, Shymko, R.M., et al. (1994). Negative cooperativity in the insulin-like growth factor-I receptor and a chimeric IGF-I/insulin receptor. Endocrinology *135*, 472–475.
Ciriello, G., Miller, M.L., Aksoy, B.A., Senbabaoglu, Y., Schultz, N., and Sander, C. (2013). Emerging landscape of oncogenic signatures across human cancers. Nat. Publ. Gr. *45*.
Dougherty, M.K., Müller, J., Ritt, D.A., Zhou, M., Zhou, X.Z., Copeland, T.D., Conrads, T.P., Veenstra, T.D., Lu, K.P., and Morrison, D.K. (2005). Regulation of Raf-1 by direct feedback phosphorylation. Mol. Cell *17*, 215–224.
Eissing, T., Conzelmann, H., Gilles, E.D., Allgöwer, F., Bullinger, E., and Scheurich, P. (2004). Bistability analyses of a caspase activation model for receptor-induced apoptosis. J. Biol. Chem. *279*, 36892–36897.
Fang, X., Yu, S., Eder, a, Mao, M., Bast, R.C., Boyd, D., and Mills, G.B. (1999). Regulation of BAD phosphorylation at serine 112 by the Ras-mitogen-activated protein kinase pathway. Oncogene *18*, 6635–6640.
Fingar, D.C., Salama, S., Tsou, C., Harlow, E., and Blenis, J. (2002). Mammalian cell size is controlled by mTOR and its downstream targets S6K1 and 4EBP1/eIF4E. Genes Dev. *16*, 1472–1487.
Friedel, C.C., Dölken, L., Ruzsics, Z., Koszinowski, U.H., and Zimmer, R. (2009). Conserved principles of mammalian transcriptional regulation revealed by RNA half-life. Nucleic Acids Res. *37*.
Fu, W., Ma, Q., Chen, L., Li, P., Zhang, M., Ramamoorthy, S., Nawaz, Z., Shimojima, T., Wang, H., Yang, Y., et al. (2009). MDM2 acts downstream of p53 as an E3 ligase to promote FOXO ubiquitination and degradation. J. Biol. Chem. *284*, 13987–14000.
Gdynia, G., Grund, K., Eckert, A., Böck, B.C., Funke, B., Macher-Goeppinger, S., Sieber, S., Herold-Mende, C., Wiestler, B., Wiestler, O.D., et al. (2007). Basal caspase activity promotes migration and invasiveness in glioblastoma cells. Mol. Cancer Res. *5*, 1232–1240.
Geltmeier, A., Rinner, B., Bade, D., Meditz, K., Witt, R., Bicker, U., Bludszuweit-Philipp, C., and Maier, P. (2015). Characterization of dynamic behaviour of MCF7 and MCF10A cells in

ultrasonic field using modal and harmonic analyses. PLoS One *10*, 1–20.
Gérard, C., and Goldbeter, A. (2009). Temporal self-organization of the cyclin/Cdk network driving the mammalian cell cycle. Proc. Natl. Acad. Sci. U. S. A. *106*, 21643–21648.
Gilley, J., Coffer, P.J., and Ham, J. (2003). FOXO transcription factors directly activate bim gene expression and promote apoptosis in sympathetic neurons. J. Cell Biol. *162*, 613–622.
Gregory, M. a, and Hann, S.R. (2000). c-Myc Proteolysis by the Ubiquitin-Proteasome Pathway : Stabilization of c-Myc in Burkitt ' s Lymphoma Cells c-Myc Proteolysis by the Ubiquitin-Proteasome Pathway : Stabilization of c-Myc in Burkitt ' s Lymphoma Cells. Mol. Cell. Biol. *20*, 2423–2435.
Haupt, Y., Maya, R., Kazaz, A., and Oren, M. (1997). Mdm2 promotes the rapid degradation of p53. Nature *387*, 296–299.
He, T.C., Sparks, A.B., Rago, C., Hermeking, H., Zawel, L., da Costa, L.T., Morin, P.J., Vogelstein, B., Kinzler, K.W., Groden, J., et al. (1998). Identification of c-MYC as a target of the APC pathway. Science *281*, 1509–1512.
Hekmat-Nejad, M., You, Z., Yee, M. ching, Newport, J.W., and Cimprich, K.A. (2000). Xenopus ATR is a replication-dependent chromatin-binding protein required for the DNA replication checkpoint. Curr. Biol. *10*, 1565–1573.
Howie, H.L., Shiflett, S.L., and So, M. (2008). Extracellular Signal-Regulated Kinase Activation by Neisseria gonorrhoeae Downregulates Epithelial Cell Proapoptotic Proteins Bad and Bim □. *76*, 2715–2721.
Hübner, A., Barrett, T., Flavell, R.A., and Davis, R.J. (2008). Multisite Phosphorylation Regulates Bim Stability and Apoptotic Activity. Mol. Cell *30*, 415–425.
Iyer, V., and Struhl, K. (1996). Absolute mRNA levels and transcriptional initiation rates in Saccharomyces cerevisiae. Proc. Natl. Acad. Sci. U. S. A. *93*, 5208–5212.
Jeong, H., Tombor, B., Albert, R., Oltvai, Z.N., and Barabasi, A.-L. (2000). The large-scale organization of metabolic networks. Nature *407*, 651–654.
Kar, S., Baumann, W.T., Paul, M.R., and Tyson, J.J. (2009). Exploring the roles of noise in the eukaryotic cell cycle. Proc. Natl. Acad. Sci. U. S. A. *106*, 6471–6476.
Karlsson-Rosenthal, C., and Millar, J.B.A. (2006). Cdc25: mechanisms of checkpoint inhibition and recovery. Trends Cell Biol. *16*, 285–292.
Kiselyov, V. V, Versteyhe, S., Gauguin, L., and De Meyts, P. (2009). Harmonic oscillator model of the insulin and IGF1 receptors' allosteric binding and activation. Mol. Syst. Biol. *5*, 243.
von Kriegsheim, A., Baiocchi, D., Birtwistle, M., Sumpton, D., Bienvenut, W., Morrice, N., Yamada, K., Lamond, A., Kalna, G., Orton, R., et al. (2009). Cell fate decisions are specified by the dynamic ERK interactome. Nat. Cell Biol. *11*, 1458–1464.
Kudla, G., Murray, A.W., Tollervey, D., and Plotkin, J.B. (2009). Coding-sequence determinants of gene expression in Escherichia coli. Science *324*, 255–258.
Kugel, J.F., and Goodrich, J. a (2000). A kinetic model for the early steps of RNA synthesis by human RNA polymerase II. J. Biol. Chem. *275*, 40483–40491.
Lahav, G., Rosenfeld, N., Sigal, A., Geva-Zatorsky, N., Levine, A.J., Elowitz, M.B., and Alon, U. (2004). Dynamics of the p53-Mdm2 feedback loop in individual cells. Nat. Genet. *36*, 147–150.
Li, X., Huang, Y., Jiang, J., and Frank, S.J. (2008). ERK-dependent threonine phosphorylation of EGF receptor modulates receptor downregulation and signaling. Cell. Signal. *20*, 2145–2155.
Luciano, F., Jacquel, A., Colosetti, P., Herrant, M., Cagnol, S., Pages, G., and Auberger, P. (2003). Phosphorylation of Bim-EL by Erk1/2 on serine 69 promotes its degradation via the

proteasome pathway and regulates its proapoptotic function. Oncogene *22*, 6785–6793.
Mamane, Y., Petroulakis, E., Rong, L., Yoshida, K., Ler, L.W., and Sonenberg, N. (2004). eIF4E--from translation to transformation. Oncogene *23*, 3172–3179.
Manning, B.D., and Cantley, L.C. (2003). United at last: the tuberous sclerosis complex gene products connect the phosphoinositide 3-kinase/Akt pathway to mammalian target of rapamycin (mTOR) signalling. Biochem Soc Trans *31*, 573–578.
Marini, E.S., Giampietri, C., Petrungaro, S., Conti, S., Filippini, a, Scorrano, L., and Ziparo, E. (2014). The endogenous caspase-8 inhibitor c-FLIPL regulates ER morphology and crosstalk with mitochondria. Cell Death Differ. 1–13.
Matsuzaki, H., Daitoku, H., Hatta, M., Tanaka, K., and Fukamizu, A. (2003). Insulin-induced phosphorylation of FKHR (Foxo1) targets to proteasomal degradation. Proc. Natl. Acad. Sci. U. S. A. *100*, 11285–11290.
McKay, M.M., and Morrison, D.K. (2007). Integrating signals from RTKs to ERK/MAPK. Oncogene *26*, 3113–3121.
De Meyts, P., and Whittaker, J. (2002). Structural biology of insulin and IGF1 receptors: implications for drug design. Nat. Rev. Drug Discov. *1*, 769–783.
Milo, R., Jorgensen, P., Moran, U., Weber, G., and Springer, M. (2009). BioNumbers The database of key numbers in molecular and cell biology. Nucleic Acids Res. *38*, 750–753.
Mohammadi, M., Olsen, S.K., and Ibrahimi, O. a (2005). Structural basis for fibroblast growth factor receptor activation. Cytokine Growth Factor Rev. *16*, 107–137.
Murphy, L.O., Smith, S., Chen, R.-H., Fingar, D.C., and Blenis, J. (2002). Molecular interpretation of ERK signal duration by immediate early gene products. Nat. Cell Biol. *4*, 556–564.
Nakakuki, T., Birtwistle, M.R.M.R., Saeki, Y., Yumoto, N., Ide, K., Nagashima, T., Brusch, L., Ogunnaike, B.A.B.A.B.A., Okada-Hatakeyama, M., and Kholodenko, B.N.B.N. (2010). Ligand-specific c-fos expression emerges from the spatiotemporal control of ErbB network dynamics. Cell *141*, 884–896.
Nakano, K., and Vousden, K.H. (2001). PUMA, a novel proapoptotic gene, is induced by p53. Mol. Cell *7*, 683–694.
Obenauer, J.C., Cantley, L.C., and Yaffe, M.B. (2003). Scansite 2.0: Proteome-wide prediction of cell signaling interactions using short sequence motifs. Nucleic Acids Res. *31*, 3635–3641.
Oda, E., Ohki, R., Murasawa, H., Nemoto, J., Shibue, T., Yamashita, T., Tokino, T., Taniguchi, T., and Tanaka, † Nobuyuki (2000). Noxa, a BH3-Only Member of the Bcl-2 Family and Candidate Mediator of p53-Induced Apoptosis. Science (80-.). *288*, 1053 LP-1058.
Ozaki, K., Kadomoto, R., Asato, K., Tanimura, S., Itoh, N., and Kohno, M. (2001). ERK pathway positively regulates the expression of Sprouty genes. Biochem Biophys Res Commun *285*, 1084–1088.
Park, C.S., Schneider, I.C., and Haugh, J.M. (2003). Kinetic analysis of platelet-derived growth factor receptor/phosphoinositide 3-kinase/Akt signaling in fibroblasts. J. Biol. Chem. *278*, 37064–37072.
del Peso, L., González-García, M., Page, C., Herrera, R., and Nuñez, G. (1997). Interleukin-3-induced phosphorylation of BAD through the protein kinase Akt. Science *278*, 687–689.
Resat, H., Ewald, J.A., Dixon, D.A., and Wiley, H.S. (2003). An integrated model of epidermal growth factor receptor trafficking and signal transduction. Biophys. J. *85*, 730–743.
Riley, T., Sontag, E., Chen, P., and Levine, A. (2008). Transcriptional control of human p53-regulated genes. Nat. Rev. Mol. Cell Biol. *9*, 402–412.

Roos, W.P., and Kaina, B. (2006). DNA damage-induced cell death by apoptosis. Trends Mol. Med. *12*, 440–450.
Schilling, C.H., and Palsson, B.O. (1998). The underlying pathway structure of biochemical reaction networks. Proc. Natl. Acad. Sci. U. S. A. *95*, 4193–4198.
Schwanhausser, B., Busse, D., Li, N., Dittmar, G., Schuchhardt, J., Wolf, J., Chen, W., and Selbach, M. (2011). Global quantification of mammalian gene expression control. Nature *473*, 337–342.
Shankaran, H., Zhang, Y., Tan, Y., and Resat, H. (2013). Model-Based Analysis of HER Activation in Cells Co-Expressing EGFR, HER2 and HER3. PLoS Comput. Biol. *9*.
Shaulian, E., and Karin, M. (2001). AP-1 in cell proliferation and survival. Oncogene *20*, 2390–2400.
Shreeram, S., Demidov, O.N., Hee, W.K., Yamaguchi, H., Onishi, N., Kek, C., Timofeev, O.N., Dudgeon, C., Fornace, A.J., Anderson, C.W., et al. (2006). Wip1 Phosphatase Modulates ATM-Dependent Signaling Pathways. Mol. Cell *23*, 757–764.
Smith, G.R., and Shanley, D.P. (2010). Modelling the response of FOXO transcription factors to multiple post-translational modifications made by ageing-related signalling pathways. PLoS One *5*.
Sonenberg, N., and Hinnebusch, A.G. (2009). Regulation of Translation Initiation in Eukaryotes: Mechanisms and Biological Targets. Cell *136*, 731–745.
Story, M.T., Hopp, K. a, Molter, M., and Meier, D. a (1994). Characteristics of FGF-receptors expressed by stromal and epithelial cells cultured from normal and hyperplastic prostates. Growth Factors *10*, 269–280.
Suter, D.M., Molina, N., Gatfield, D., Schneider, K., Schibler, U., and Naef, F. (2011). Mammalian Genes Are Transcribed with Widely Different Bursting Kinetics. Science (80-.). *332*, 472–474.
Takahashi, M., Ota, S., Nishimura, S., Ogura, K., Maeda, S., Toda, N., Hamada, E., Terano, A., and Omata, M. (1996). Keratinocyte growth factor is an endogenous stimulant of rabbit gastric epithelial cell proliferation and migration in primary culture. J. Gastroenterol. Hepatol. *11*, 1089–1096.
Tani, H., Mizutani, R., Salam, K.A., Tano, K., Ijiri, K., Wakamatsu, A., Isogai, T., Suzuki, Y., and Akimitsu, N. (2012). Genome-wide determination of RNA stability reveals hundreds of short-lived noncoding transcripts in mammals. Genome Res. *22*, 947–956.
Tawa, P., Hell, K., Giroux, a, Grimm, E., Han, Y., Nicholson, D.W., and Xanthoudakis, S. (2004). Catalytic activity of caspase-3 is required for its degradation: stabilization of the active complex by synthetic inhibitors. Cell Death Differ. *11*, 439–447.
Tewari, M., Quan, L.T., O'Rourke, K., Desnoyers, S., Zeng, Z., Beidler, D.R., Poirier, G.G., Salvesen, G.S., and Dixit, V.M. (1995). Yama/CPP32 beta, a mammalian homolog of CED-3, is a CrmA-inhibitable protease that cleaves the death substrate poly(ADP-ribose) polymerase. Cell *81*, 801–809.
Thattai, M., and van Oudenaarden, A. (2001). Intrinsic noise in gene regulatory networks. Proc. Natl. Acad. Sci. U. S. A. *98*, 8614–8619.
Tzivion, G., Dobson, M., and Ramakrishnan, G. (2011). FoxO transcription factors; Regulation by AKT and 14-3-3 proteins. Biochim. Biophys. Acta - Mol. Cell Res. *1813*, 1938–1945.
Wilhelm, M., Schlegl, J., Hahne, H., Gholami, A.M., Lieberenz, M., Savitski, M.M., Ziegler, E., Butzmann, L., Gessulat, S., Marx, H., et al. (2014). Mass-spectrometry-based draft of the human proteome. Nature *509*, 582–587.

Zhang, X., Tang, N., Hadden, T.J., and Rishi, A.K. (2011). Akt, FoxO and regulation of apoptosis. Biochim. Biophys. Acta - Mol. Cell Res. *1813*, 1978–1986.

Zhang, X.-Y., Birtwistle, M.R., and Gallo, J.M. (2014). A General Network Pharmacodynamic Model-Based Design Pipeline for Customized Cancer Therapy Applied to the VEGFR Pathway. CPT Pharmacometrics Syst. Pharmacol. *3*, e92.
